# Continuous subcutaneous insulin infusion versus multiple daily injection regimens in children and young people at diagnosis of type 1 diabetes: pragmatic randomised controlled trial and economic evaluation

**DOI:** 10.1136/bmj.l1226

**Published:** 2019-04-03

**Authors:** Joanne C Blair, Andrew McKay, Colin Ridyard, Keith Thornborough, Emma Bedson, Matthew Peak, Mohammed Didi, Francesca Annan, John W Gregory, Dyfrig A Hughes, Carrol Gamble

**Affiliations:** 1 Department of Endocrinology, Alder Hey Children’s NHS Foundation Trust, Liverpool L12 2AP, UK; 2 Clinical Trials Research Centre, University of Liverpool, Liverpool, UK; 3 Centre for Health Economics and Medicines Evaluation, Bangor University, Bangor, UK; 4 Department of Diabetes, Alder Hey Children’s NHS Foundation Trust, Liverpool, UK; 5 Department of Research, Alder Hey Children’s NHS Foundation Trust, Liverpool, UK; 6 Division of Population Medicine, School of Medicine, Cardiff University, Cardiff, UK

## Abstract

**Objective:**

To compare the efficacy, safety, and cost utility of continuous subcutaneous insulin infusion (CSII) with multiple daily injection (MDI) regimens during the first year following diagnosis of type 1 diabetes in children and young people.

**Design:**

Pragmatic, multicentre, open label, parallel group, randomised controlled trial and economic evaluation.

**Setting:**

15 paediatric National Health Service (NHS) diabetes services in England and Wales. The study opened to recruitment in May 2011 and closed in January 2017.

**Participants:**

Patients aged between 7 months and 15 years, with a new diagnosis of type 1 diabetes were eligible to participate. Patients who had a sibling with the disease, and those who took drug treatments or had additional diagnoses that could have affected glycaemic control were ineligible.

**Interventions:**

Participants were randomised, stratified by age and treating centre, to start treatment with CSII or MDI within 14 days of diagnosis. Starting doses of aspart (CSII and MDI) and glargine or detemir (MDI) were calculated according to weight and age, and titrated according to blood glucose measurements and according to local clinical practice.

**Main outcome measures:**

Primary outcome was glycaemic control (as measured by glycated haemoglobin; HbA1c) at 12 months. Secondary outcomes were percentage of patients in each treatment arm with HbA1c within the national target range, incidence of severe hypoglycaemia and diabetic ketoacidosis, change in height and body mass index (as measured by standard deviation scores), insulin requirements (units/kg/day), partial remission rate (insulin dose adjusted HbA1c <9), paediatric quality of life inventory score, and cost utility based on the incremental cost per quality adjusted life year (QALY) gained from an NHS costing perspective.

**Results:**

294 participants were randomised and 293 included in intention to treat analyses (CSI, n=144; MDI, n=149). At 12 months, mean HbA1c was comparable with clinically unimportant differences between CSII and MDI participants (60.9 mmol/mol *v* 58.5 mmol/mol, mean difference 2.4 mmol/mol (95% confidence interval −0.4 to 5.3), P=0.09). Achievement of HbA1c lower than 58 mmol/mol was low among the two groups (66/143 (46%) CSII participants *v* 78/142 (55%) MDI participants; relative risk 0.84 (95% confidence interval 0.67 to 1.06)). Incidence of severe hypoglycaemia and diabetic ketoacidosis were low in both groups. Fifty four non-serious and 14 serious adverse events were reported during CSII treatment, and 17 non-serious and eight serious adverse events during MDI treatment. Parents (but not children) reported superior PedsQL scores for those patients treated with CSII compared to those treated with MDI. CSII was more expensive than MDI by £1863 (€2179; $2474; 95% confidence interval £1620 to £2137) per patient, with no additional QALY gains (difference −0.006 (95% confidence interval −0.031 to 0.018)).

**Conclusion:**

During the first year following type 1 diabetes diagnosis, no clinical benefit of CSII over MDI was identified in children and young people in the UK setting, and treatment with either regimen was suboptimal in achieving HbA1c thresholds. CSII was not cost effective.

**Trial registration:**

Current Controlled Trials ISRCTN29255275; European Clinical Trials Database 2010-023792-25.

## Introduction

Type 1 diabetes is a common, chronic disease of childhood, affecting about 26 000 infants, children, and young people in the United Kingdom.^1^ Treatment requires administration of subcutaneous insulin in doses calculated according to carbohydrate consumption, physical activity, and blood glucose measurements. During childhood and adolescence, poor glycaemic control is associated with impaired memory,^2^ poorer cognitive outcomes,^3^ an increased risk of depression,^4^ and poor growth.^5^ In the longer term, vascular complications lead to blindness, renal failure, premature heart disease, stroke, and amputation.^6^ The risk of developing complications is related to glycaemic control, and is lower in patients treated with intensive regimens on insulin treatment: multiple daily injections (MDI) and continuous subcutaneous insulin infusion (CSII).^7^ There is no cure for type 1 diabetes, so optimal treatment is essential to enable the best possible quality of life and the effective use of healthcare resources while minimising the risk of complications.

A meta-analysis of six randomised controlled trials involving 165 children reported a modest benefit of CSII treatment on glycaemic control (as measured by glycated haemoglobin (HbA1c); −0.24%, 95% confidence interval −0.41% to −0.07%)^8^ albeit below the threshold associated with better clinical outcomes (0.5%),^7^ but no difference in the risk of severe hypoglycaemia or diabetic ketoacidosis.^8^ However, key limitations need to be considered when interpreting these results. 

Firstly, in five of the included randomised controlled trials, the observation period was insufficient (seven months or less) for the effects of treatment fatigue to be observed, and might not have been long enough for patients to become fully competent in the use of CSII. Secondly, isophane insulin use in the MDI arm of five included studies limits the generalisability of the results to modern regimens using longacting insulin analogues. Thirdly, in five of the six trials, patients with established type 1 diabetes treated with MDI were randomised to continue MDI treatment or change to CSII, which introduced selection bias. Patients in whom MDI treatment is satisfactory are less likely to be invited or to consent to participate than those patients in whom treatment is inadequate. In a more recent study, patients with established type 1 diabetes in whom MDI treatment was inadequate were randomised to either continue MDI treatment for six months or change to CSII; beneficial effects of CSII on quality of life were reported, but no effect on HbA1c was seen.^9^ In a small randomised trial comparing CSII with MDI in patients newly diagnosed with type 1 diabetes and observed for two years, researchers saw no difference in glycaemic control or adverse events between treatment arms.^10^ Finally, at the start of CSII treatment, there is a period of intense education and frequent contact with diabetes healthcare professionals, which could favourably affect glycaemic control independently of CSII treatment in those patients with established diabetes. In the REPOSE study, when adults with type 1 diabetes and poor glycaemic control were randomised to treatment with CSII or MDI and given equivalent education, no additional benefit from CSII was identified.^11^

Observational studies of national paediatric databases from the United States, UK, Germany, and Austria have reported an association between CSII treatment and superior glycaemic control^12^ ^13^ with only a modest effect. CSII use is lower in patients from ethnic minorities and those with greatest socioeconomic deprivation.^14^ ^15^ ^16^ Given that glycaemic control and severe hypoglycaemia are independently related to ethnicity and deprivation,^14^ ^15^ ^16^ there is a risk of bias, inherent to observational data, in estimates of the effect of CSII in these studies.

The cost of type 1 diabetes to the UK’s health service is significant, with estimates ranging from £1bn (€1.17bn; $1.33bn) to £1.8bn a year^17^ ^18^ and expected to be nearly 2% of total NHS expenditure over the next two decades.^17^ A cost effectiveness analysis from the REPOSE study concluded that routine use of CSII in adults, without an immediate clinical need, would not be cost effective.^19^ The economic evidence, which indicates more expensive treatment with CSII to be cost effective in paediatrics,^20^ ^21^ relies on data from limited trials and extensive modelling. Use of CSII in paediatric practice increased from 14% of patients in 2011 to 28% in 2015-16.^1^ The widespread adoption of CSII, with little evidence of treatment superiority compared with MDI, requires an adequately powered randomised controlled trial, designed to address areas of bias inherent in previous studies. We therefore conducted the SCIPI trial (subcutaneous insulin: pumps or injections?), in which we recruited children and young people newly diagnosed with of type 1 diabetes and compared outcomes after one year of treatment.

## Methods

### Trial design

The study protocol has been previously published.^22^ In brief, we conducted a pragmatic, multicentre, open label, parallel group, randomised controlled trial, randomising participants to CSII or MDI to compare the treatments’ efficacy, safety, and cost utility. The study was conducted in paediatric diabetes services experienced in the use of CSII, in nine university and six local hospitals within the NHS in England and Wales. The study opened to recruitment in May 2011 and was closed in January 2017. The study protocol (supplementary appendix) was approved by the Liverpool East research ethics committee, reference 10/H1002/80.

An internal pilot tested study feasibility, and the standard deviation used to inform the power calculation. A consent rate of at least 50%, with no differences likely to be of clinical significance in demographic criteria for age, ethnicity, sex, and deprivation score between those patients who consented and declined to participate was required to proceed to the full study. Study sites were selected on the basis of the availability of a core set of experienced staff who had completed a recognised course on insulin pump therapy and had their competencies assessed and authorised.

### Participants

Patients aged between 7 months and 15 years and with new diagnoses of type 1 diabetes were eligible to participate. Patients with the following characteristics were ineligible: previous treatment for type 1 diabetes, haemoglobinopathy, coexisting conditions or treatment likely to affect glycaemic control, psychological or psychiatric disorder, an allergy to a component of insulin aspart or determir (Novo Nordisk, Gatwick, UK) or insulin glargine (Sanofi, Guildford, UK), and a sibling with type 1 diabetes. Patients with thyroid disease or coeliac disease were eligible if thyroid hormone concentrations or coeliac antibodies demonstrated good adherence to treatment. Patients and carers were given written and age appropriate information about the study at diagnosis, supplemented by a video presented by participants and parents from February 2014. Written informed consent or, where appropriate, assent was obtained from carers and participants.

### Randomisation

Patients were randomised to treatment with CSII or MDI using 1:1, web based, block randomisation stratified by age (7 months to <5 years, 5 years to <12 years, ≥12 years) and by treating centre. Participants were recruited by members of their local diabetes service and research nurses trained in the recruitment of paediatric patients. Parents and carers, and, when appropriate, patients, were invited to share reasons for declining to participate.

### Procedures

We collected the following data at baseline, from the time of diagnosis of type 1 diabetes: blood pH, blood glucose, HbA1c, thyroid function tests, anti-islet cell and anti-glutamic acid decarboxylase antibodies, and tissue transglutaminase or other antibody test for coeliac disease measured per local practice before consent. These measurements were made prior to consent and did not form part of the study protocol. All participants completed a structured educational programme, which covered the syllabus outlined by the International Society for Paediatric and Adolescent Diabetes.^23^ This included the cause of type 1 diabetes, the use and administration of insulin, hyperglycaemia and correction doses of insulin, hypoglycaemia symptoms and treatment, exercise, sick day rules, carbohydrate counting, the benefits of maintaining optimal glycaemic control for long term health, and blood glucose monitoring. The number of education sessions was recorded to ensure parity across treatment arms. All participants received training on the use of MDI regimen and the Expert glucometer, with participants randomised to CSII receiving additional training in the use of CSII. All advanced pump features were taught, used, and regularly reviewed.

Randomised treatment started within 14 days of diagnosis of type 1 diabetes. Baseline height and weight were documented on the day randomised treatment began. Participants randomised to CSII were treated with insulin aspart, and those randomised to MDI with the shortacting insulin analogue insulin aspart and a longacting insulin analogue, either insulin glargine or detemir, according to local clinical practice. Insulin doses were calculated according to weight and age (see study protocol, web appendix 1), and titrated against blood glucose readings according to local protocols. Participants in both treatment arms used a glucometer that included a so-called bolus wizard, which calculated insulin doses according to blood glucose readings and carbohydrate consumption.

Study visits coincided with clinic appointments at three, six, nine, and 12 months. At each visit, the following data were collected: HbA1c, adverse events, height, weight, concomitant treatment and insulin use from prescriptions, glucometer and insulin pump downloads (CSII), and patient kept records (MDI). Participants and carers documented home episodes of severe hypoglycaemia^24^ and diabetic ketoacidosis in a diary. Treatment diaries and telephone logs were assessed at each study visit for any treatment related adverse events and related serious adverse events.^25^

In addition to self reporting, local hospital databases were interrogated at each clinical assessment to ascertain whether the participant had been treated for a related serious adverse event in the preceding three months. Adverse events were classified according to relation with the injection device, glucometer, insulin, errors in insulin administration, or incidental illness. The Health Utilities Index questionnaire^26^ was administered at baseline and each study visit, and the diabetes module of PedsQL (paediatric quality of life inventory)^27^was completed at six and 12 months.

We measured resource use by using questionnaires and accessing prescription records and electronic, patient linked, information costing systems. These data included the purchase of pumps or MDI injection devices and associated consumables; purchase of insulins; contact with healthcare services including family doctors and school nurses; and hospital inpatient, outpatient, and emergency department attendances.

Data were collected on paper based case report forms and questionnaires, and entered centrally at the clinical trials unit into MACRO (InferMed, London, UK), a compliant clinical data management system. Bespoke software was developed to receive data downloaded from glucometers and pumps.

### Outcome measures

The primary outcome measure was HbA1c 12 months following diagnosis of type 1 diabetes. Blood samples were analysed locally and centrally at Alder Hey Children’s Hospital, Liverpool, using the Siemens DCA Vantage HbA1c analyser. Within batch precision is less than 6% and between batch precision is less than 8%. A limits of agreement analysis was undertaken for measurements made in different laboratories and by “point of care” methods.^28^ Sensitivity analyses were performed using samples analysed centrally only, at point of care only, and central in preference to point of care if both were available. 

Secondary outcome measures were the percentage of patients in each treatment arm with HbA1c within the national target range^29^; incidence of severe hypoglycaemia (hypoglycaemia associated with altered consciousness) and diabetic ketoacidosis; change in standard deviation scores (SDS) of height and body mass index; insulin requirements (units/kg/day); partial remission rate, defined as insulin dose adjusted HbA1c less than 9 (HbA1c (%) + (4×insulin dose (units/kg/day)))^30^; PedsQL score; and cost utility based on the incremental cost per quality adjusted life year (QALY) gained.

### Statistical analysis

SCIPI was designed as a superiority trial assuming a null hypothesis of no difference. To detect a difference in HbA1c means of 0.5% (or 5.46 mmol/mol conversion),^31^ the minimum difference generally considered to be clinically meaningful,^7^ with common standard deviation of 1.50 using a two group *t* test with a 0.05 two sided significance level, 286 participants (143 per group) were required to achieve 80% power. To allow for 10% loss to follow-up, the recruitment target was increased to 316 participants (158 per group).

The sample size calculation was based on HbA1c 12 months following diagnosis.^32^ At the time of designing the trial, the association between HbA1c at diagnosis and longer term outcomes was speculative. Baseline HbA1c reflects blood glucose in the previous three months before insulin treatment. Two exploratory analyses were considered to include HbA1c measured at baseline as a continuous explanatory variable.

The intention-to-treat principle was used for the primary analysis such that all randomised participants in whom the outcome was observed were included in the group to which they were randomly allocated. We used a 0.05 level of statistical significance and 95% confidence intervals throughout. The statistical and health economic analysis plans^33^ were developed before analysis and are available as supplementary material. Analyses were conducted using SAS software (version 9.2; SAS Institute, Cary, NC, USA) or Stata (version 13; StataCorp, College Station, TX, USA). Independent statisticians from within the clinical trials unit produced and monitored the randomisation lists and statistical analysis plan,^33^ and undertook quality control via independent programming for the primary outcome and safety analyses. Blinding of the trial statistician was not possible, but inclusion of every participant within each analysis set was determined before use of allocation information.

For the primary outcome (HbA1c 12 months after randomisation), we used least squares regression adjusted for age category and centre as a random effect. Owing to the expected low incidence of events, secondary binary outcomes were presented as unadjusted relative risks. We undertook a per protocol analysis for the primary outcome to check the robustness of conclusions to major prespecified protocol deviations (table S2). Body mass index and height were standardised by use of WHO growth standards,^34^ ^35^ and analysed by analysis of covariance including respective baseline measures, age group, and treatment group as covariates in the model with centre fitted as a random effect. Insulin requirements (units/kg/day) were calculated to reflect insulin use over a four week period, and then analysed as per growth outcomes without baseline measure to reflect the absence of insulin use in this untreated population before randomisation. PedsQL overall score (0-100) at 12 months was calculated as per PedsQL guidelines according to the age specific questionnaires used, and then analysed as per growth outcomes. Partial remission rate at 12 months (defined as insulin dose adjusted HbA1c <9) was calculated using HbA1c, weight, and daily insulin dose, and then analysed as per binary outcomes. 

We conducted a safety analysis on adverse event data according to the method of insulin delivery at the time of the event. The incidence density rate was used to quantify the number of patients with at least one new case per population at risk in a given time period. The denominators were the sum of the person time in years for each treatment group (accounting for treatment switches) of the population at risk. For the cost utility analysis, we used UK Health Utilities Index Mark 2 tariffs^26^ to estimate utilities and used the trapezoidal rule to calculate QALYs. Resource use was costed from the perspective of the NHS using the national tariff and other national unit costs. We compared the ratio of the differences between intervention groups in costs and QALYs with the NHS cost effectiveness threshold of £20 000 per QALY. The joint uncertainty in costs and QALYs was considered through 10 000 Bootstrap replicates (bias corrected and accelerated). A lifetime modelled extrapolation was only planned if differences in HbA1c were apparent between intervention groups at 12 months.

### Patient and public involvement

Study design, delivery, and data interpretation were undertaken in close discussion with patients and their families. Young people were consulted on the design of the study including impact of participation, outcome measures, and study materials. Parents of children and young people with type 1 diabetes were members of the trial management committee and trial steering committee and advised on recruitment strategy. Study results and their significance to patients and their families were discussed in detail with parent contributors.

## Results

### Internal pilot

Recruitment data from the internal pilot study showed acceptable consent rates, showed no evidence of patient selection bias, and supported the parameters used in the sample size calculation. The oversight committees recommended progression to the full study. Data from patients recruited to the internal pilot study were included in the full study.

### Study participants

**Fig 1:**
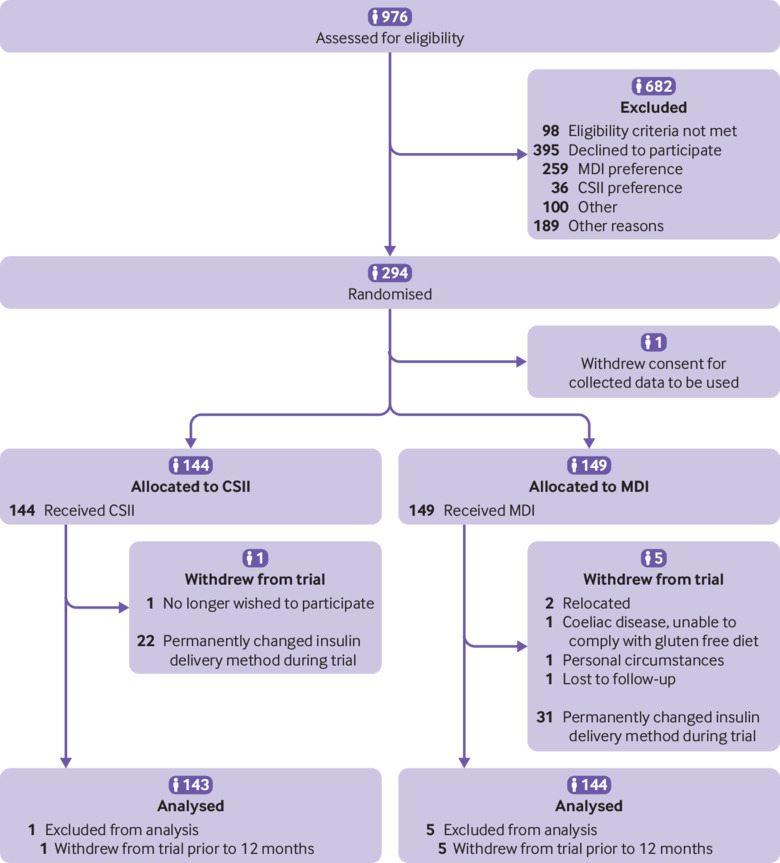
CONSORT diagram illustrating patient flow from diagnosis to completion of the study protocol. CSII=continuous subcutaneous insulin infusion; MDI=multiple daily injections

Between May 2011 and January 2016, 976 patients diagnosed as having type 1 diabetes were assessed for eligibility in 15 study centres. Of 689 patients who were eligible and approached for consent, 294 (43%, CSII=144, MDI=149) consented to participate. One patient withdrew before starting their randomised treatment. The sample size calculation was inflated to 316 participants to allow for 10% attrition, but the observed attrition was lower such that the trial was stopped after 294 randomisations to provide the numbers required to achieve 80% power (fig 1). Of patients invited to participate in SCIPI who declined to be randomised, 66% (259/395) stated that they (or their parent/carer) had a strong preference for MDI and 10% (39/395) stated that they had a strong preference for CSII.

Of those randomised to CSII, 80% (91/114) of participants and 92% (130/142) of parents/carers received their favoured preferred treatment, compared with 37% (41/112) of participants and 28% (42/148) of parents/carers randomised to MDI (P<0.001). Age, sex, ethnicity, and deprivation score did not differ between those who consented to participate and those who declined (table S1), or between treatment arms (table 1).

**Table 1:** Baseline characteristics of study participants

	Continuous subcutaneous insulin infusion (n=144)	Multiple daily injections (n=149)	Total (n=293)
**Age at randomisation**			
Median (interquartile range)	9.9 (5.7-12.2)	9.4 (5.8-12.5)	9.8 (5.7-12.3)
**Age group (No (%))**			
7 months to <5 years	33 (22.9)	32 (21.5)	65 (22.2)
5 to <12 years	71 (49.3)	76 (51)	147 (50.2)
12 to 15 years	40 (27.8	41 (27.5)	81 (27.6)
**Sex (No (%))**			
Female	71 (49.3)	69 (46.3)	140 (47.8)
Male	73 (50.7)	80 (53.7)	153 (52.2)
**Ethnicity (self reported)**			
Missing data (No)	1	3	4
Asian or Asian British (No (%))	3 (2)	3 (2)	6 (2)
Black or British black (No (%))	0	3 (2)	3 (1)
British white (No (%))	124 (87)	118 (81)	242 (84)
Indian (No (%))	2 (1)	2 (1)	4 (1)
Mixed (No (%))	4 (3)	6 (4)	10 (4)
Other white (No (%))	6 (4)	8 (6)	14 (5)
Other (No (%))	3 (2)	2 (1)	5 (2)
Pakistani (No (%))	1 (1)	4 (3)	5 (2)
Deprivation score*			
Missing data (No)	7	6	13
Median (interquartile range)	19.4 (8.9-37.9)	14.7 (7.8-31.8)	17 (8.4-35.8)
**Body mass index† SDS**			
Missing data (No)	20	17	37
Mean (standard deviation)	0.2 (1.3)	0.1 (1.4)	0.1 (1.3)
**Height SDS**			
Missing data (No)	20	17	37
Mean (standard deviation)	0.3 (1.1)	0.3 (1.1)	0.3 (1.1)
**HbA1c (mmol/mol)**			
Missing data (No)	12	18	30
Mean (standard deviation)	104.6 (24.4)	102.6 (26.7)	103.6 (25.5)
**Blood glucose (mmol/L)**			
Missing data (No)	3	3	6
Mean (standard deviation)	26.8 (9.2)	26.9 (10)	26.9 (9.6)
**Blood pH**			
Missing data (No)	17	16	33
Mean (standard deviation)	7.3 (0.2)	7.3 (0.1)	7.3 (0.2)

SDS=standard deviation score.

* Deprivation score ranges from 0 to 100, with 100 indicating greater deprivation.

† Body mass index=weight (kg) divided by the square of height (m).

### Adherence to the protocol

A CONSORT diagram illustrating the pathway of patients from diagnosis to study completion is given in figure 1. Retention and adherence data are reported in figure S1 and table S2. All participants received their allocated interventions other than one participant who withdrew consent immediately after randomisation. Of 144 participants initially assigned to CSII, 21 (15%) switched to MDI, and 30 (20%) of 149 participants switched from MDI to CSII. Primary outcome data were available for 97% (285/293) of participants. Primary and secondary outcome measures are provided in table 2 and table 3.

**Table 2:** Primary and secondary outcome measures (continuous)

Continuous outcomes	Total No of participants, adjusted mean (95% CI)		Adjusted mean difference (95% CI)	P
	CSII (n=144)	MDI (n=149)		
HbA1c (mmol/mol) at 12 months (primary outcome)				
Intention-to-treat analysis*†	143, 60.9 (58.5 to 63.3)	142, 58.5 (56.1 to 60.9)	2.4 (−0.4 to 5.3)	0.09
Per protocol†	87, 60.2 (56.4 to 63.9)	66, 59.3 (55.3 to 63.2)	0.9 (−3.2 to 5.0)	0.67
Change in body mass index SDS*‡	122, 0.6 (0.8)	122, 0.5 (0.8)	0.1 (0 to 0.3)	0.13
Change in height SDS*§	122, −0.1 (0.5)	122, 0 (0.4)	−0.1 (−0.2 to 0)	0.10
Insulin requirements (units/kg/day)*†	87, 0.7 (0.2)	64, 0.6 (0.3)	0.1 (0.0 to 0.2)	0.01

CSII=continuous subcutaneous insulin infusion; MDI=multiple daily injections; SDS=standard deviation score.

* Intention-to-treat analysis.

† Analysis adjusted for randomisation group (age category, fixed effects; centre, random effects).

‡ Analysis adjusted for randomisation group (age category, fixed effects; centre, random effects) and body mass index standard deviation score at baseline.

§ Analysis adjusted for randomisation group (age category, fixed effects; centre, random effects) and height standard deviation score at baseline.

**Table 3:** Secondary outcome measures (binary)

Binary outcomes	Total No, No (%) of participants		Relative risk (95% CI)	P
	CSII (n=144)	MDI (n=149)		
HbA1c <58 mmol/mol*†	143, 66 (46.2)	142, 78 (54.9)	0.84 (0.67 to 1.06)	0.16
HbA1c <48 mmol/mol*†	143, 22 (15.4)	142, 29 (20.4)	0.75 (0.46 to 1.25)	0.28
Incidence of severe hypoglycaemia*	144, 6 (4.2)	149, 2 (1.3)	3.1 (0.6 to 15.1)	0.17
Incidence of diabetic ketoacidosis*	144, 2 (1.4)	149, 0	5.2 (0.3 to 106.8)	0.24
Partial remission (IDAAC <9)*	86, 21 (24.4)	64, 21 (32.8)	0.74 (0.45 to 1.24)	0.28

CSII=continuous subcutaneous insulin infusion; MDI=multiple daily injections; IDAAC=insulin dose adjusted glycated haemoglobin (HbA1c).

* Intention-to-treat analysis.

† 58 mmol/mol was the national target up to August 2015; 48 mmol/mol was the target set in August 2015.

### Primary outcome

The treatment arms were comparable for HbA1c at 12 months, with differences between the treatment arms being small and unimportant in the intention-to-treat analysis (CSII, n=143, MDI, n=142; table 2; table S3 shows full primary outcome results split by age group) and per protocol analysis. Sensitivity analyses demonstrated robustness of results to the measurement of HbA1c at central laboratory and point of care (table S4). Figure S2 shows details of HbA1c by age group and at each time point. The study was not powered to detect differences in glycaemic control between age groups. However, the observed HbA1c values were generally lower for the youngest and oldest age groups during MDI treatment, although there is a lot of uncertainty when comparing across groups. A forest plot showed the stability of treatment effect over time (figure S3), despite changes in NHS diabetes care during the SCIPI trial. Forest plots of the primary outcome split by subgroup (figure S4) also showed consistency of treatment effect across age groups and SCIPI centres.

The trial was powered to detect a difference in HbA1c between groups at 12 months unadjusted for baseline HbA1c values. The prognostic value of significance of HbA1c at diagnosis of type 1 diabetes was not well recognised at the time SCIPI opened to recruitment. Two exploratory analyses were considered to include HbA1c measured at baseline as a continuous explanatory variable (table S5). These results did not alter the SCIPI study conclusions for CSII compared with MDI at 12 months (adjusted mean difference 2.9 mmol/mol, 95% confidence interval −0.02 to 5.9), but did suggest the importance of early baseline values for 12 month measurements (HbA1c level baseline coefficient estimate 0.07; standard error 0.03, 95% confidence interval 0.01 to 0.13).

A second exploratory analysis considered the effect of deprivation (table S7). Although we saw an association between higher HbA1c values at baseline and at 12 months with greater deprivation, the conclusions remained unaltered (adjusted mean differences in HbA1c at 12 months between treatment groups (CSII-MDI) were 2.9 mmol/mol (95% confidence interval −0.02 to 5.9) and 2.2 mmol/mol (−0.7 to 5.0), respectively).

### Secondary outcomes

Secondary outcomes were analysed as per intention to treat (table 2 and table 3). We saw no difference in the number of participants achieving HbA1c targets (<58 mmol/mol, the national target until August 2015; and <48 mmol/mol, the target set in August 2015^28^). Change in body mass index and height SDS were similar between treatment arms. 

Insulin dose data were available for 52% of participants (CSII, n=87/144, MDI, n=64/149). Insulin requirements were higher in those treated with CSII (adjusted mean difference 0.1 units/kg/day, 95% confidence interval 0.0 to 0.2, P=0.01), primarily in the oldest participants. The basal bolus ratio for patients treated with CSII, across the lifetime of the study, was 0.8. The ratio started from 0.73 at one month fluctuating up and down throughout the course of the trial, ending at 0.67 at 12 months (figure S5 and table S6). Similar data for MDI were not as robust because they depended on patient reporting.

Eight episodes of severe hypoglycaemia (CSII=6, MDI=2) and two episodes of diabetic ketoacidosis (CSII=2, MDI=0) were reported. Safety dataset reports events were categorised according to the treatment the participant received at the time of the adverse event, and took into account temporary or permanent switches in the method of insulin delivery. The total number of events experienced and the number of participants experiencing at least one event were provided, along with the incidence density rate (defined as the number of patients with at least one new adverse event per population at risk in a given time period).

Of 54 non-serious adverse events reported in 36 participants treated with CSII at the time of the adverse event, 29 were related to the insulin pump, with an incidence density rate of 25.0 participants and at least one event per 100 person years. Of 17 non-serious adverse events reported in 16 participants (incidence density rate 10.5 participants) treated with MDI at the time of the adverse event, two were related to the injection device. Fourteen serious adverse events were reported in nine participants (incidence density rate 6.2 participants) treated with CSII at the time of the serious adverse event, and eight serious adverse events in eight participants (incidence density rate 5.3 participants) treated with MDI at the time of the serious adverse event. Adverse event data are summarised in table 4. Patients randomised to CSII had twice as many related emergency department visits and inpatient stays related to type 1 diabetes (122 visits made by 35/144 patients), compared with those randomised to MDI (60 visits made by 25/149 patients; mean difference 0.4 per patient, 95% confidence interval 0.1 to 0.9). Reasons for those adverse events recorded as serious are provided in table 4.

**Table 4:** Information regarding serious adverse events and non-serious adverse events recorded during trial period

Category and description of adverse event	Non-serious adverse events						Serious adverse events				
	CSII (144.1 total person years, n=144)			MDI (151.9 total person years, n=149)			CSII (144.1 total person years, n=144)			MDI (151.9 total person years, n=149)	
	No of events	Patients (IDR)		No of events	Patients (IDR)		No of events	Patients (IDR)		No of events	Patients (IDR)
**All**											
Diabetic ketoacidosis	2	2 (1.4)		0	0 (0)		2	2 (1.4)		0	0 (0)
Insulin administration error	2	2 (1.4)		5	5 (3.3)		2	2 (1.4)		3	3 (2)
Pump failure	4	3 (2.1)		0	0 (0)		0	0 (0)		0	0 (0)
Severe hypoglycaemia	6	6 (4.2)		2	2 (1.3)		1	1 (0.7)		0	0 (0)
Site infections	8	7 (4.9)		0	0 (0)		1	1 (0.7)		0	0 (0)
Other - to specify	32	22 (15.3)		10	10 (6.6)		8	6 (4.2)		5	5 (3.3)
**Device**											
Diabetic ketoacidosis	1	1 (0.7)		0	0 (0)		1	1 (0.7)		0	0 (0)
Pump failure	4	3 (2.1)		0	0 (0)		0	0 (0)		0	0 (0)
Severe hypoglycaemia	2	2 (1.4)		0	0 (0)		0	0 (0)		0	0 (0)
Site infections	8	7 (4.9)		0	0 (0)		1	1 (0.7)		0	0 (0)
Other - to specify	14	11 (7.6)		3	3 (2)		0	0 (0)		0	0 (0)
**Carer error**											
Insulin administration error	1	1 (0.7)		4	4 (2.6)		0	0 (0)		0	0 (0)
Other - to specify	5	2 (1.4)		0	0 (0)		1	1 (0.7)		0	0 (0)
**Meter error**											
Other - to specify	3	3 (2.1)		1	1 (0.7)		5	4 (2.8)		2	2 (1.3)
**Incidental illness**											
Insulin administration error	1	1 (0.7)		0	0 (0)		1	1 (0.7)		0	0 (0)
Other - to specify	5	5 (3.5)		3	3 (2)		0	0 (0)		1	1 (0.7)
**Other**											
Diabetic ketoacidosis	1	1 (0.7)		0	0 (0)		1	1 (0.7)		0	0 (0)
Insulin administration error	0	0 (0)		1	1 (0.7)		3	2 (1.4)		3	3 (2)
Severe hypoglycaemia	4	4 (2.8)		2	2 (1.3)		1	1 (0.7)		2	2 (1.3)
Other - to specify	5	4 (2.8)		3	3 (2)		0	0 (0)		0	0 (0)

CSII=continuous subcutaneous insulin infusion; MDI=multiple daily injections; IDR=incidence density rate.

Child reported PedsQL scores (diabetes module) at 12 months were available for 71% of individuals (CSII, n=104; MDI, n=104), but 26 children in each treatment group were too young to complete the questionnaire. An adjusted mean difference at 12 months of 3.1 (95% confidence interval −0.6 to 6.8) favoured CSII, but the result was not statistically significant. Parents of participants (CSII, n=128/144, MDI, n=123/149) reported a statistically significantly higher score with CSII that with MDI (adjusted mean difference 4.1 (95% confidence interval 0.6 to 7.6)), but this result should be interpreted against meaningful differences being five points or more.^36^

We found 4.3 (95% confidence interval 0.6 to 8.0) more contacts with healthcare professionals per participant treated with CSII (21.2) than MDI (16.9), using texts, emails, and phone calls. Mean total costs were higher by £1863 (95% confidence interval £1620 to £2137) for CSII than for MDI; with the most of this difference (£1177) from the additional cost of consumables and device (undiscounted annual cost of £600 for CSII versus £80 for MDI; table 5). We saw no significant difference in QALYs between CSII (0.910) and MDI (0.916; mean difference −0.006, 95% confidence interval −0.031 to 0.018). The probability of CSII being more expensive and less effective than MDI was 0.69, with no likelihood of CSII being cost effective at a threshold of £20 000 per QALY.

**Table 5:** Resource use in participants treated with CSII versus MDI

Items of resource use	Cost (£; mean (95% CI))		
	CSII	MDI	Difference
Device (pump* with four year lifespan or two pen devices)	600 (596 to 606)	80 (80 to 80)	520 (516 to 526)
Consumables* (eg, needles, infusion sets, reservoirs)	1841 (1826 to 1861)	664 (664 to 664)	1177 (1162 to 1197)
Insulin (prescribed)	422 (364 to 486)	482 (426 to 541)	−60 (−142 to 24)
Healthcare professional contacts (telephone calls, faxes, texts, or emails)	138 (117 to 162)	108 (92 to 124)	30 (3 to 59)
Scheduled outpatients visits	434 (434 to 434)	434 (434 to 434)	0 (0 to 0)
Unscheduled outpatient visits	309 (272 to 346)	328 (292 to 366)	−19 (−71 to 33)
Inpatient stays costed from healthcare resource groups	387 (245 to 553)	219 (142 to 306)	168 (5 to 352)
Emergency department visits	26 (16 to 39)	13 (8 to 19)	13 (2 to 27)
Other hospital related items (eg, ward visits)	3 (1 to 7)	3 (1 to 5)	1 (−3 to5)
Family doctor visits	71 (56 to 88)	57 (45 to 69)	15 (−5 to 35)
Home visits	106 (80 to 138)	83 (66 to 100)	23 (−9 to 59)
School visits	53 (43 to 64)	56 (44 to 69)	−3 (−19 to 13)
Concomitant treatments	12 (8 to 17)	15 (8 to 23)	−2 (−12 to 6)
Total cost	4404 (4197 to 4642)	2541 (2412 to 2672)	1863 (1620 to 2137)

£1=€1.16; $1.32. CSII=continuous subcutaneous insulin infusion; MDI=multiple daily injections;

* 25% discount not included.

## Discussion

### Principal findings

In this randomised controlled trial of paediatric patients with a new diagnosis of type 1 diabetes, CSII treatment was neither more clinically effective nor more cost-effective than MDI, by the standards of the NHS. This non-superiority was consistent across centres and strengthens the lack of evidence to support CSII. There is evidence that glycaemic control in the first year of diagnosis is predictive of longer term outcomes,^37^ ^38^ and this is likely to be a critical period of care. Partial recovery of insulin production during the first year of diagnosis could substantially alter the treatment paradigm compared with the later stages in the course of type 1 diabetes, and our findings should not be applied beyond the first year of diagnosis.

### Strengths and limitations of study

Our data are strengthened by a high retention rate and consistency of age, sex, ethnicity, and deprivation between treatment arms. Furthermore, age, sex, ethnicity, and deprivation did not differ between those patients who consented and those who declined to participate. Participants were recruited at diagnosis of type 1 diabetes, and core diabetes education and contact with healthcare professionals was balanced across treatment arms.

Our recruitment rate was lower than predicted and was strongly influenced by early treatment preference. The diagnosis of type 1 diabetes has been associated with symptoms of posttraumatic stress disorder in parents,^39^ and for some families it may not have been possible to contemplate randomisation to a new treatment so soon after diagnosis. Had we deferred recruitment, we might have achieved higher recruitment rates. At the point of randomisation, those patients randomised to treatment with CSII were significantly more likely to have received their preferred treatment and it is likely that we recruited a population of patients favouring CSII, which could explain the higher numbers of patients switching from MDI to CSII during follow-up. Examination of glycaemic control at the time of switching did not indicate poorer control. Future studies should examine how preference and disappointment might influence utilisation of randomised treatments.

The intention-to-treat analysis included all participants in the group that they were randomised to while the per protocol analysis excluded participants with major protocol deviations, which included switching method of insulin delivery. This difference allows some consideration of the effect, and although both analyses were not significant, the conclusions are robust. In addition, to account for participants who switched method of insulin delivery, the safety population analysed participants in the group to the method of insulin delivery at the time of the safety event.

The National Paediatric Diabetes Audit (NPDA) reports improvements in glycaemic control from 2010-11 (72 mmol/mol in England and 70 mmol/mol in Wales) to 2016-17 (64 mmol/mol, both in England and Wales).^1^ During this period, several national initiatives have been undertaken that are likely to have contributed to this sustained improvement. However, only 15% of patients treated with CSII and 20% of patients treated with MDI achieved an HbA1c within the target range at the end of the first year of treatment. Glycaemic control is poorer in the UK than other European and North America country, where CSII is used more commonly,^11^ leading to speculation that increased use of CSII could improve glycaemic control. The relative inexperience of NHS practitioners in CSII treatment could have obscured the potential benefits of this treatment. However, study sites were selected on the availability of a core set of trained and experienced staff. We saw no evidence of a treatment effect over time, and block randomisation ensured balance between treatment arms.

The development, validation, documentation, and monitoring of an education package and treatment protocols would have strengthened the study, but this would have incurred significant additional cost and delays, in the absence of robust evidence to inform the development of these protocols. Standardisation of educational packages is ideal, but it is important that these can be individualised to meet the needs of patients and their families. The pace at which education can be delivered to an unselected cohort of patients with newly diagnosed diabetes will be more measured than education of selected patients experienced in the treatment of type 1 diabetes, and it might be unrealistic to expect all families to achieve a high level of sophistication in CSII use. Additional education in CSII use could have reduced the prevalence of adverse events in this arm and improved glycaemic control, although this should be set in the context of the adult study, INPUT, which reported no effect of a structure education programme on glycaemic control in patients treated with CSII.^40^

Many adverse events were reported in the study cohort, which is consistent with the background population of patients with childhood type 1 diabetes treated with CSII. The NPDA reports that CSII treatment increased the risk of being admitted to hospital for diabetic ketoacidosis by 23%, and of being admitted to hospital for reasons other than diabetic ketoacidosis or hypoglycaemia by 27%. CSII treatment did not confer benefit or increased risk from admission with hypoglycaemia.^41^

The speed of technological developments outpaces the time required to deliver a clinical trial. It could be argued that the findings of the SCIPI trial are outdated: technology has advanced, clinical teams have greater experience of CSII, and improved education programmes and psychological support equips patients and their families to manage this treatmemt more successfully with fewer adverse events. However, observational data from the most mature CSII services report benefits in HbA1c below thresholds thought to be clinically meaningful,^9^ ^13^ taking no account of the effect of deprivation or ethnicity on clinical outcomes. Enhanced education and psychological support also has the potential to improve quality of life and clinical outcomes in patients treated with MDI. To improve the timeframes required to deliver the evidence, development of a clinical trial platform should be considered.

### Comparison with other studies

Our findings are consistent with those reported in a smaller randomised controlled trial of patients with newly diagnosed type 1 diabetes,^9^ and previous studies of patients with the established disease.^10^ ^42^ ^43^ ^44^ Authors of a systematic review of adult and paediatric patients concluded that CSII enabled superior glycaemic control and quality of life than MDI with fewer episodes of hypoglycaemia, but cautioned that the inclusion of observational data could have introduced bias.^45^

The reported effect of CSII on quality of life and treatment satisfaction varies.^10^ ^40^ ^41^ ^42^ In our study, parents of participants treated with CSII reported a small, but significantly higher PedsQL score for the quality of life of their children. A qualitative approach could detect differences in quality of life that were not identified in our questionnaire based approach. No adjustments for multiplicity were applied to secondary outcomes, and SCIPI was not powered to detect differences within these outcomes. Consequently, the results should not be judged solely on the presence or absence of statistical significance.

Tools for recording insulin use were less robust in those treated with MDI than CSII, and difficulties with data downloads from glucometers and pumps, and missing data in handheld records resulted in a large amount of missing data. In contrast with previous studies,^13^ ^18^ ^20^ insulin requirements were higher in patients treated with CSII than MDI, which could reflect a reduction in the intensity of MDI treatment as older participants gain independence, or under-reporting. Recognising this uncertainty, our data relating to partial remission should be interpreted with caution.

### Conclusions and policy implications

Many patient advocacy groups and healthcare professionals are of the strong opinion that treatment with CSII is beneficial and further research should focus on determining what these perceived benefits are and on developing validated tools to measure them. Individual patients are likely to experience benefits from this treatment that are not directly associated with the outcomes measured in this study. For example, preschool children who consume carbohydrates and exercise erratically could benefit from a treatment with fewer injections and a basal insulin profile that can be modified readily. For the SCIPI cohort, longer term observation is required to assess how treatment in the first year has influenced the trajectory of glycaemic control in future years.

Evolving technology that automates glucose monitoring and insulin dose adjustment has the potential to reduce the burden of CSII treatment on patients and families, and enable superior glycaemic control to that reported in SCIPI participants. These technologies are likely to be considerably more expensive than those evaluated in this study and evidence should be sought to support their use.

In considering the outcomes of the SCIPI study it is important to recognise three points. Firstly, glycaemic control was suboptimal in both treatment arms. Secondly, patients recruited to the study were newly diagnosed, and more favourable results could be achieved with CSII in patients more experienced in the treatment of type 1 diabetes. Thirdly, advances in technology could reduce the burden of CSII treatment, and facilitate superior control in time.

In resource limited settings, the introduction of novel, expensive treatment should be informed by robust clinical data demonstrating superiority. Data from the SCIPI study indicate that the use of CSII was neither clinically beneficial nor cost effective in the first year of type 1 diabetes, and resources could be more effectively invested in other measures to improve glycaemic control.

**What is already known on this topic**

Intensive insulin regimens, multiple daily injections (MDI) and continuous subcutaneous insulin infusion (CSII), are associated with superior glycaemic control in patients with type 1 diabetes
Meta-analyses and economic evaluations reporting CSII to be a cost effective treatment are based on small randomised controlled trials and observational data, and are subject to considerable modelling
Results of a cluster randomised trial in adults with type 1 diabetes (REPOSE) did not support a policy of providing insulin pumps over multiple daily injections

**What this study adds**

In this randomised controlled trial and economic evaluation of infants, children, and young people in the first year of type 1 diabetes, glycaemic control was suboptimal in both treatment arms
CSII treatment was not more clinically effective than treatment with MDI; furthermore, CSII as a standalone treatment was not cost effective
Parents of children treated with CSII, but not the children themselves, reported superior quality of life for their children compared with parents of children treated with MDI, below thresholds thought to be clinically significant

## Web extra

Extra material supplied by authors

**Web appendix 1:** Study protocol

**Web appendix 2:** Supplementary material

## Notes

Contributors:****JCB developed the study protocol in collaboration with coinvestigators, oversaw the delivery of the study, prepared study update reports, oversaw clinical aspects of the statistical analysis plan, clinical interpretation of study data, led the preparation of the final report, and is the guarantor. CG contributed to the study design, protocol development, data capture methods, and the preparation of the manuscript (drafting, reviewing, editing); and led the involvement of the clinical trials unit. AM undertook the final statistical analyses under the supervision of CG, prepared data for reports throughout the study, prepared data for the tables and figures for the manuscript, and contributed to the preparation of the manuscript (drafting, reviewing, editing); DAH contributed to protocol development, oversaw the health economic aspects of the study and the final analyses, and contributed to the manuscript (drafting, reviewing, editing); CR undertook health economics analysis, managed all aspects of health economics elements of the study, and contributed to the manuscript (drafting, reviewing, editing); MP contributed to protocol development, guidance on study delivery, supported interpretation of clinical data, and contributed to manuscript (drafting and reviewing); JWG contributed to protocol development, guidance on study delivery, supported interpretation of clinical data, and contributed to the manuscript (reviewing and editing); MD contributed to protocol development, guidance on study delivery, supported interpretation of clinical data, and contributed to the manuscript (reviewing); FA contributed to the study design, prepared educational materials for study sites, and contributed to the manuscript (reviewing); EB gave guidance and support on all aspects of governance and study delivery, supported the preparation of progress reports, and contributed to the manuscript (reviewing); KT recruited patients, provided guidance and expertise to research nurses in study sites, led research nurse teleconferences and face-to-face meetings, supported interpretation of clinical study data, and contributed to the manuscript (reviewing). The corresponding author attests that all listed authors meet authorship criteria and that no others meeting the criteria have been omitted.

Ethical approval: The study protocol (supplementary appendix) was approved by the Liverpool East research ethics committee, UK, reference 10/H1002/80. Written informed consent and (where appropriate) assent were obtained from carers and participants.

Data sharing: We are committed to sharing anonymised individual patient data for the purpose of research for the benefit of patients with bonefide researchers. Requests for data should be sent to the Clinical Trials Research Centre, University of Liverpool.

The lead author (the manuscript’s guarantor) affirms that the manuscript is an honest, accurate, and transparent account of the study being reported; that no important aspects of the study have been omitted; and that any discrepancies from the study as planned and registered have been explained.
