# Supplementary material for: Continuous subcutaneous insulin infusion versus multiple daily injection regimens in children and young people at diagnosis of type 1 diabetes: pragmatic randomised controlled trial and economic evaluation
Source: BMJ. 2019 Apr 3;365:l1226. doi: 10.1136/bmj.l1226 (PMC6446076; doi:10.1136/bmj.l1226)
Supplement: Supplementary file 1 — Web appendix 1: Study protocol [file blaj046029.ww1.pdf]

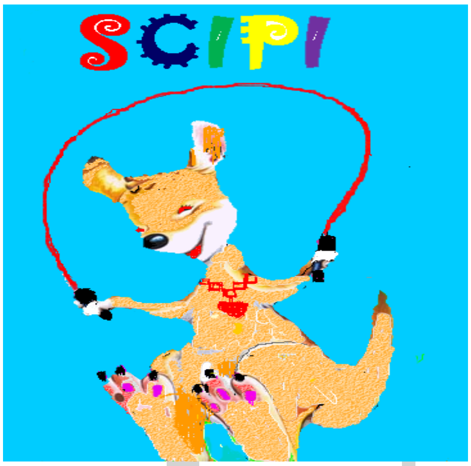

SubCutaneous Insulin: Pumps or Injections

Randomised controlled trial of continuous subcutaneous insulin infusion compared to multiple daily injection regimens in children and young people at diagnosis of type I diabetes mellitus.

Version 7.0 (01/08//2016)

Study Sponsor:
Alder Hey Children's
NHS Foundation Trust
Eaton Road
Liverpool
L12 2AP

EudraCT number : 2010-023792-25
ISRCTN number : ISRCTN29255275
MREC reference : 10/H1002/80
CTA number : 21362/0002/001-0001
Funder reference : HTA 08/14/39
Website: www.scipitrial.org.uk

CTRC
Clinical Trials Research Centre

HTA
NIHR Health Technology Assessment programme funded project

Protocol Approval

Authorised by Chief Investigator:

Signature: ______________________ **Date:** 08/09/2016

Dr Joanne Blair
Consultant Endocrinologist and Honorary Senior Lecturer
Lead, NIHR Children's Research Network, North West Coast
Alder Hey Children's NHS Foundation Trust
Eaton Rd
Liverpool
L12 2AP
Telephone Number: 0151 252 5281
Fax Number: 0151 252 4606
Email : Jo.Blair@alderhey.nhs.uk

Authorised on behalf of the Sponsor:

Signature: L M Cooper ______________________ **Date:** 08/09/2016

Lucy Cooper
Research Governance and Quality Assurance Lead
Clinical Research Business Unit
2nd Floor, Institute in the Park
Alder Hey Children's NHS Foundation Trust
Eaton Rd
Liverpool
L12 2AP
Telephone Number: 0151 252 5570
E-mail: Lucy.Cooper@alderhey.nhs.uk

Authorised By Trial Statistician:

Signature: ______________________ **Date:** 24/08/2016

Carrol Gamble
Trial Statistician
University of Liverpool
Institute of Child Health
Alder Hey Children's NHS Foundation Trust
Eaton Rd
Liverpool
L12 2AP
Telephone Number: 0151 282 4724
E-mail: c.gamble@liverpool.ac.uk

General Information
This document describes the SCIPI trial and provides information about procedures for entering patients into it. The protocol should not be used as an aide-memoir or guide for the treatment of other patients; every care was taken in its drafting, but corrections or amendments may be necessary. These will be circulated to the registered investigators in the trial, but centres entering patients for the first time are advised to contact the Medicines for Children Research Network Clinical Trials Unit to confirm they have the most up to date version. Clinical problems relating to this trial should be referred to the relevant Chief Investigator via the Medicines for Children - Clinical Trial Unit (MC-CTU).

This protocol defines the participant characteristics required for study entry and the schedule of treatment and follow-up. Participant recruitment will be undertaken in compliance with this document and applicable regulatory and governance requirements. Waivers to authorise non-compliance are not permitted.

Incidence of protocol non-compliance, whether reported prospectively (e.g. where a treatment cannot be administered on a scheduled date as a result of public holidays) or retrospectively noted (e.g. as a result of central monitoring) are recorded as protocol deviations, the incidence of which are monitored and reported to trial oversight committees.

Statement of Compliance
This trial will be carried out in accordance with the World Medical Association Declaration of Helsinki (1964) and the Tokyo (1975), Venice (1983), Hong Kong (1989) and South Africa (1996) amendments and will be conducted in compliance with the protocol, Clinical Trials Research Centre (CTRC) Standard Operating Procedures (SOPs) and EU Directive 2001/20/EC, transposed into UK law as the UK Statutory Instrument 2004 No 1031: Medicines for Human Use (Clinical Trials) Regulations 2004 as amended.

The trial is comparing alternative methods of insulin delivery via CE-marked medical devices employed for their intended purpose. Therefore, this trial is not considered to be a clinical investigation under the Medical Devices Regulations 2002.

Relationship Statements
The UK Clinical Research Collaboration (UKCRC; www.ukcrc.org) is a partnership organisation working to establish the UK as a world leader in clinical research. Following a review by an international panel, the CTRC at the University of Liverpool has been assessed as reaching the highest quality standard required by the UKCRC and achieved full UKCRC registration.

The CTRC encompasses clinical trials activity in areas including medicines for children MC-CTU, cancer (The Liverpool Cancer Trials Unit; LCTU) and developing portfolios, (http://www.ctrc.org.uk/). All CTRC activities are underpinned by methodological rigour, a modern data management system, similar technical requirements and a common set of SOPs.

The NIHR Medicines for Children Research Network, Diabetes Research Network and National Cancer Research Network are part of the National Institute for Health Research Clinical Research Network.

Contact Details: Institutions

Sponsor:	Trial Management, Monitoring and Analysis:	Clinical Laboratory:
Alder Hey Children's NHS Foundation Trust<br>Eaton Rd<br>Liverpool L12 2AP<br>Tel: 0151 252 5570<br>email: Lucy.Cooper@alderhey.nhs.uk	Medicines for Children<br>Clinical Trials Unit<br>University of Liverpool<br>Institute of Child Health<br>Alder Hey Children's NHS Foundation Trust<br>Liverpool<br>L12 2AP<br>Tel: 0151 794 9764<br>Fax: 0151 282 4721<br>email: scipi@liverpool.ac.uk	Paul Newland<br>Alder Hey Children's NHS Foundation Trust<br>Eaton Rd<br>Liverpool L12 2AP<br>Tel: 0151 252 5488<br>Fax: 0151 293 3617<br><br>e-mail:<br>Paul.Newland@alderhey.nhs.uk

Contact Details: Individuals

Individual Authorised to Sign the Protocol and Protocol Amendments on behalf of the Sponsor:	Chief Investigator (CI):	Medical Expert who will Advise on Protocol Related Clinical Queries (If other than CI):
Lucy Cooper<br>Alder Hey Children's NHS Foundation Trust<br>Eaton Rd Liverpool L12 2AP<br>Tel.: 0151 252 5570<br>email:<br>Lucy.Cooper@alderhey.nhs.uk	Dr Joanne Blair, MB;ChB, MRCPCH, MRCP, MD<br>Alder Hey Children's NHS Foundation Trust<br>Eaton Rd<br>Liverpool L12 2AP<br>Tel.: 0151 252 5281<br>Fax: 0151 252 4606<br>email: Jo.Blair@alderhey.nhs.uk	Dr Mohammed Didi<br>MB;BS,MRCP<br>Alder Hey Children's NHS Foundation Trust<br>Eaton Rd<br>Liverpool L12 2AP<br>Tel.: 0151 252 5281<br>Fax : 0151 282 4606<br>email:mohammed.didi@alderhey.nhs.uk
**Medical Expert who will Evaluate SAE Reports (If other than CI):**		
Dr Mohammed Didi<br>MB;BS,MRCPAlder Hey Children's NHS Foundation Trust<br>Eaton Rd<br>Liverpool L12 2AP<br>Tel.: 0151 252 5281<br>Fax : 0151 282 4606<br>email:<br>mohammed.didi@alderhey.nhs.uk		

The contact details of other individuals involved in the trial are detailed in the Trial Oversight Committee Membership document.

Table of Contents

Glossary 9

1 Protocol Summary 11

2 Background Information 13
- 2.1 Introduction 13
- 2.2 Rationale 16
- 2.3 Objectives 17
- 2.4 Potential Risks and Benefits 18
  - 2.4.1 Potential Risks 18
  - 2.4.2 Known Potential Benefits 18

3 Selection of Centres/Clinicians 19
- 3.1 Centre/Clinician Inclusion Criteria 19
- 3.2 Centre/Clinician Exclusion Criteria 19

4 Trial design 20
- 4.1 Pilot Study 20
- 4.2 Full Trial 20

5 Study Population 21
- 5.1 Inclusion Criteria 21
- 5.2 Exclusion Criteria 21
- 5.3 Participant Transfer and Withdrawal 21
  - 5.3.1 Participant Transfers 21
  - 5.3.2 Withdrawal from Trial Intervention 22
  - 5.3.3 Withdrawal from Trial Completely 22

6 Enrolment and Randomisation 23
- 6.1 Screening 23
- 6.2 Baseline 23
- 6.3 Randomisation 23

7 Trial Treatment/s 25
- 7.1 Introduction 25
- 7.2 Education for both arms 25
- 7.3 Formulation, Packaging, Labelling, Storage and Stability 26
- 7.4 Preparation, Dosage and Administration of Study Treatment/s 27

Starting Dose Calculations: 27
- 7.5 Dose Modifications 28
- 7.6 Accountability Procedures for Study Treatment/s 28
- 7.7 Assessment of Compliance with Study Treatment/s 28
- 7.8 Concomitant Medications/Treatments 29
- 7.9 Co-enrolment Guidelines 29

8 Assessments and Procedures 30
- 8.1 Schedule for Follow-up 30

Table 1: Trial Assessments 31
- 8.2 Procedures for assessing Efficacy 32
  - 8.2.1 HbA1c 32
  - 8.2.2 Insulin requirements 33
  - 8.2.3 Blood glucose measurements 33
- 8.3 Procedures for Assessing Safety 33
  - 8.3.1 Adverse events: 33
  - 8.3.2 Body Mass Index (BMI): 33
- 8.4 Other Assessments 35
  - 8.4.1 Quality of Life 35

8.4.2 Health Economics analysis: 36
8.5 Loss to Follow-up 36
8.6 Trial Closure 37

9 Statistical Considerations 38
9.1 Introduction 38
9.2 Method of Randomisation 38
9.3 Outcome Measures 38
9.4 Sample Size 38
9.5 Interim Monitoring and Analyses 38
9.6 Analysis Plan 39
9.7 Economic analysis 39

10 Pharmacovigilance 41
10.1 Terms and Definitions 41
10.1.1 Medicines for Human Use (Clinical Trials) Regulations 2004 (SI 2004/1031) Definitions 41
10.1.2 National Research Ethics Service (NRES) Definitions 42
10.1.3 Medicines and Healthcare products Regulatory Agency (MHRA) Adverse Incident Centre (AIC) Definitions 42
10.2 Severity / Grading of Adverse Events 43
10.3 Relationship to disease under study 43
10.4 Relationship to Trial Treatment 43

Table 2: Definitions of Causality 43
10.5 Expectedness 44
10.6 Reporting Procedures 44
10.6.1 Reporting of Pregnancy 46
10.7 Follow-up After Adverse Events 46
10.8 Quarantine, Labelling & Storage of Devices Involved in an Adverse Incident (i.e. Related Unexpected AE/SAE) 46
10.9 Responsibilities – Investigator 47
10.10 Responsibilities – MC-CTU 48
10.11 Safety reports 49

11 Ethical Considerations 50
11.1 Ethical Considerations 50
11.2 Ethical Approval 50
11.3 Informed Consent Process 51
11.4 Study Discontinuation 52

12 Regulatory Approval 53

13 Trial Monitoring 54
13.1 Source Documents 54
13.2 Data Capture Methods 55
13.3 Data Monitoring at MC-CTU 55
13.4 Clinical Site Monitoring and Direct Access to Data 56
13.5 Central Monitoring 56
13.6 Confidentiality 56
13.7 Quality Assurance and Control 57
13.8 Records Retention 57

14 Indemnity 59

15 Financial Arrangements 60
15.1 Financial Support for Collaborating Sites 60
15.1.1 Staffing 60
15.1.2 Intervention Supplies from Roche 60

16 Trial Committees 61
16.1 Trial Management Group (TMG) 61
16.2 Trial Steering Committee (TSC) 61
16.3 Independent Data and Safety Monitoring Committee (IDSMC) 61
17 Publication 62
18 Protocol Amendments 63
18.1 Version 2.0 (30/MAR/2011) 63
18.2 Version 3.0 (01/JULY/2011) 63
18.3 Version 4.0 (17/August/2012) 64
18.4 Version 5.0 (21/JAN/2015) 66
18.5 Version 6.0 (16/FEB/2016) 67
18.6 Version 7.0 (01/AUG/2016) 67
19 References 69

Glossary

AE	Adverse Event
AI	Adverse Incident
AIC	Adverse Incident Centre
AR	Adverse Reaction
BMI	Body Mass Index
CCRN	Comprehensive Clinical Research Network
CE	Conformance mark
CI	Chief Investigator
CORE	Centre for Outcomes Research
CRF	Case Report Form
CSII	Continuous Subcutaneous Insulin Infusion
CTIMP	Clinical Trial Investigational Medicinal Product
CTRC	Clinical Trials Research Centre
CTU	Clinical Trials Unit
DCCRG	Diabetes Control and Complications Research Group
Degree C	Degree Celsius
DKA	Diabetic ketoacidosis
DRN	Diabetes Research Network
Eudract	European Union Drug Regulating Authorities Clinical Trials
GM	gram
GP	General Practitioner
$HbA1_c$	Glycosylated haemoglobin
HUI2/3	Health Utilities Index Mark 2 and 3
ICER	The Incremental Cost-Effectiveness Ratio
IDSMC	Independent Data and Safety and Monitoring Committee
IEC	Independent Ethical Committee
IMP	Investigational Medicinal Product
ISRCTN	International Standard Randomised Controlled Trial Number
kg	kilogram
LCTU	Liverpool Cancer Trials Unit
IREC	Local Research Ethics Committee
MC CTU	Medicines for Children Clinical Trials Unit
MDI	Multiple Daily Injections (4 or more)
MHRA	Medicines and Healthcare products Regulatory Agency
MREC	Multi-centre Research Ethics Committee
NDA	National Diabetes Audit
NHS	National Health Service
NICE	National Institute for Clinical Excellence
NIHR CRN	National Institute for Health Research Clinical Research Network
NIHR HTA	National Institute for Health Research Health Technology Assessment Programme
PCT	Primary Care Trust
PedsQL	Paediatric Quality Of Life Inventory
PI	Principal Investigator
QALY	Quality-Adjusted Life Year
QoL	Quality of Life
RCT	Randomised Controlled Trial
REC	Research Ethics Committee
RN	Research Nurse<br>When RN is referred to in this protocol it means either the diabetes research nurse or member of the research team who has been delegated that duty

R&D	Research and Development
RUSAE	Related and Unexpected Serious Adverse Event
SAE	Serious Adverse Event
SAP	Statistical Analysis Plan
SAR	Serious Adverse Reaction
SCIPI	Subcutaneous infusion: Pumps or Injections
SDS	Standard Deviation Score
SDV	Source Data Verification
SOP	Standard Operating Procedure
SPC	Summary of Product Characteristics
SUSAR	Suspected Unexpected Serious Adverse Reaction
TIDM	Type 1 Diabetes Mellitus
TM	Trial Management
TMG	Trial Management Group
TSC	Trial Steering Committee
U	Unit
UAR	Unexpected Adverse Reaction
VAT	Value Added Tax

1 PROTOCOL SUMMARY

Title : SCIPI (Subcutaneous Insulin: Pumps or Injections)
Open label 2-arm randomised controlled trial with an inbuilt pilot of continuous subcutaneous insulin infusion (CSII) compared to multiple daily injection (MDI) regimens in children and young people at diagnosis of type I diabetes mellitus (TIDM).

Phase : III

Population: 316 patients for full study including 30 patients for pilot study.
Inclusion Criteria: Newly diagnosed patients with TIDM aged between 7 months and 15 years (inclusive); Parent/legal representative of the patient are able to comply with the treatment regimen and study visits and are willing to give consent for the study; Patient aged 8 years and over are able to comply with the treatment regimen and study visits.
Exclusion Criteria: Patients who have been previously treated for TIDM; have a sibling with existing TIDM; are suffering from haemoglobinopathy; co-existing pathology (e.g. cystic fibrosis); receipt of medication (e.g. systemic or high dose topical corticosteroid or growth hormone therapy) likely to affect glycaemic control; psychological or psychiatric disorders (e.g. eating disorders); allergy to a component of insulin aspart or insulin glargine; known thyroid condition and in a non euthyroid state; and known Coeliac disease unable to maintain gluten free diet.

Study Centres: UK centres (hospitals) with expertise in the management of children and young people with diabetes.

Study Duration: Participant follow-up is three monthly until one year from diagnosis.

Description of Agent/ Intervention:
MDI Treatment: Participants will be treated with Lantus 100 Units/ml solution for injection (insulin glargine) once or twice daily according to their need and boluses of NovoRapid 100 U/ml solution for injection (insulin aspart) when 10 gm or more of carbohydrate are consumed using Insulin Pen Injection Device.
CSII Treatment: Participants will be treated with NovoRapid 100 U/ml solution for injection (insulin aspart) using basal insulin infusion by CSII Insulin Pumps with bolus doses of insulin aspart when 5gm or more carbohydrate is consumed.

Primary Objective/s:
Inbuilt pilot study: To acquire an understanding of the acceptability of randomisation to MDI or CSII at diagnosis of TIDM in children and young people.

Full study: To compare the glycaemic control assessed by $HbA1_c$ at 12 months after diagnosis in children and adolescents receiving CSII with those receiving MDI.

Secondary Objective/s:
Inbuilt pilot study: To define the characteristics of patients who consent and those who do not consent to randomisation. To use the data as an internal pilot to check the size of the standard deviation used in the sample size calculation of the full study.

Full study: To compare the two intervention groups for percentage of participants with $HbA1_c$ less than 6.5 % (48 mmol/mol), incidence of severe hypoglycaemia, incidence of diabetic

ketoacidosis, change in BMI and in height, insulin requirements (units / kg /day), quality of life and cost effectiveness (incremental cost per QALY gained, 12 months, life time analysis).

Schematic Representation of Study Design

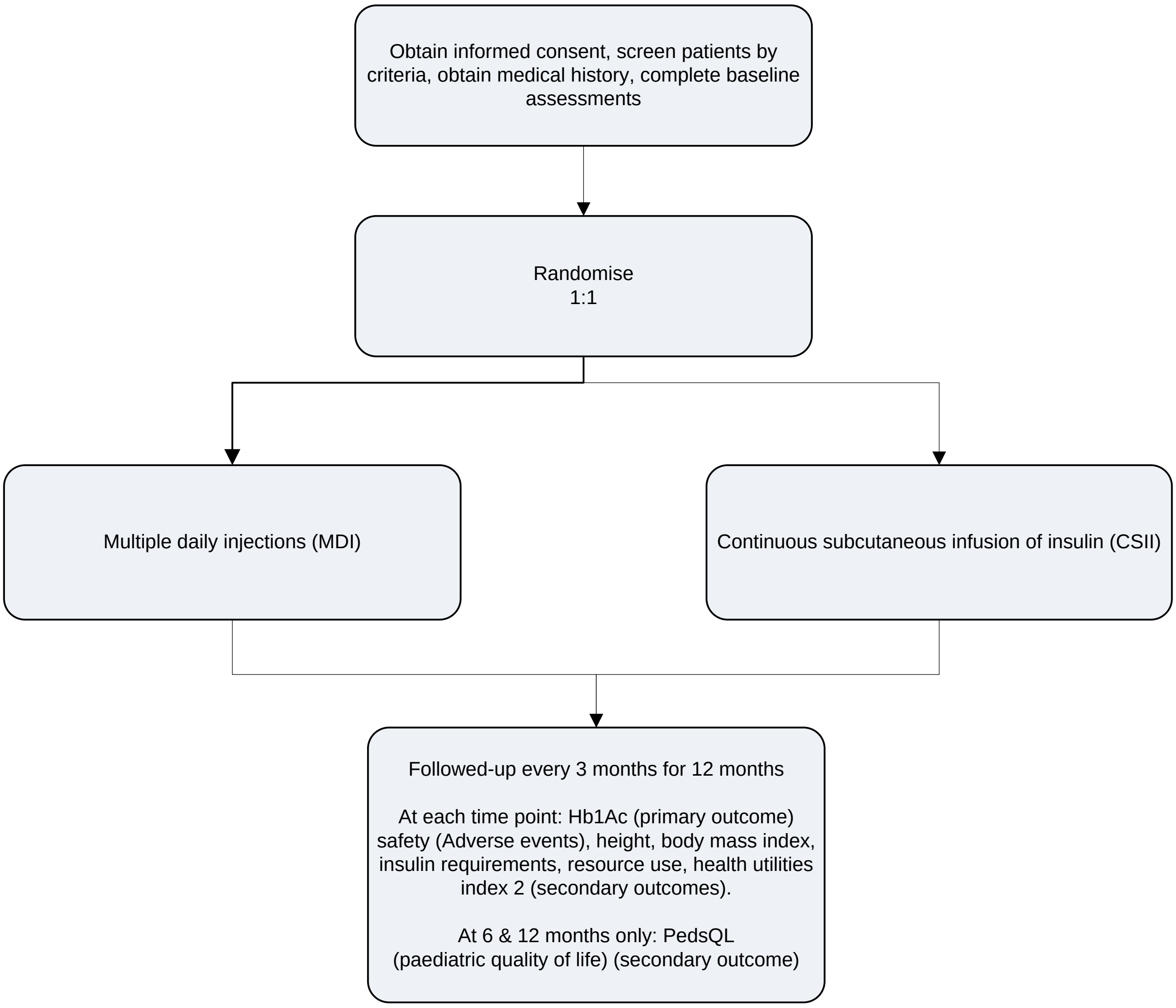

2 BACKGROUND INFORMATION

2.1 Introduction

Type I diabetes mellitus (TIDM) is a common disease of childhood. Data submitted to the National Diabetes Audit (NDA)(1) (2) for the period 2006-7 indicated that 24,000 children aged 16 years or less were affected in the UK. A sustained increase in the incidence of TIDM in childhood in Europe has been observed for more than 50 years (3,4) and the increase is most pronounced in children aged less than 5 years(5). The daily management of TIDM is burdensome requiring two or more daily injections of insulin, blood glucose monitoring and dietary manipulation. However it is the long term vascular complications of the disease which have greatest impact on morbidity, mortality and well-being. Compared to the general population adults with diabetes are three times more likely to suffer a myocardial infarction, four times more likely to develop renal failure, 11 times more likely to undergo a minor amputation and seven times more likely to undergo a major amputation(1).

The risk of acquiring these complications of TIDM is directly related to glycaemic control, duration of diabetes, insulin sensitivity and weight. By the time patients diagnosed with diabetes during childhood enter adult services many will have lived with diabetes for 10 years or more; this includes a period of pubertal growth and development during which the changes in hormonal milieu and the psychological changes that accompany them make glycaemic control particularly challenging. The aim of this study is to investigate two methods of insulin delivery during childhood and adolescence to identify which facilitates superior glycaemic control, and to examine the impact of treatment modalities on other predictors of vascular complications of TIDM, adverse events and quality of life.

Glycaemic control, intensive insulin therapy and risk of microvascular disease

In 1993 The Diabetes Control and Complications Research Group (DCCRG) (6) published conclusive evidence that both the degree and duration of hyperglycaemia are critical determinants of the risk of retinopathy and nephropathy. A strong exponential relationship between glycaemic control (measured by $HbA1_c$) and both retinopathy and nephropathy was reported. The reported median levels of $HbA1_c$ were: 7.3% in subjects treated intensively with multiple daily injections of insulin (MDI) or continuous subcutaneous insulin infusion (CSII); 9.1% in those treated using conventional insulin regimes. During an observation period of 6.5 years, in subjects treated intensively compared with those using conventional insulin regimen the risk of acquiring retinopathy or nephropathy was reduced by 76% and 39% respectively. Glycaemic control exerted greatest influence on acquisition or progression of microvascular complications however a significant benefit persisted in intensively treated patients following correction for $HbA1_c$.

Patients from both groups benefited from improved glycaemic control. Those patients who reduced their $HbA1_c$ by 10% (for example 9.0% to 8.1%) reduced their risk of acquiring retinopathy and nephropathy by 39% and 25% respectively. On the basis of these data the **National Institute of Clinical Excellence (NICE)**(7) **recommended that $HbA1_c$ be maintained below 7.5%,** a target achieved by only 18% of children and young people reported in the NDA. In contrast 30% of children and young people have very poor glycaemic control: $HbA1_c$ greater than 9.5%. In order to facilitate optimal control NICE also

recommend that children aged 12 years and above who cannot achieve HbA1c levels below 8.5% without disabling hypoglycaemia despite a high level of care should be offered a trial of CSII. **Children aged less than 12 years of age should be offered CSII therapy from diagnosis of TIDM where MDI is considered impractical or inappropriate** (8). However, there are currently no robust data demonstrating that the use of CSII facilitates improved glycaemic control in this group of patients. There is a real risk that whole sale adoption of CSII in line with the NICE guideline will deny health care professionals the opportunity to objectively assess the benefits and effectiveness of this therapy. This is of increased importance given the recent changes in NICE guidelines which now recommend a tightening in glycaemic control such that $HbA1_c$ should be maintained below 6.5% (48 mmol/mol). Although there is an abence of paediatric studies to support this recommendation the guidance was based upon other NICE guidance in conjunction with clinical and patient experience (9).

Intensive insulin therapy in childhood and adolescence

The wide scale introduction of CSII is likely to have significant economic implications for the NHS. If data submitted to the NDA are extrapolated to the rest of the British population it can be estimated that 6,400 young people aged 12 or more qualify for MDI or CSII therapy according to the recommendations of NICE(8). At present, an insulin infusion pump costs £2,500 + VAT, and consumables an additional £1,322 a year. However, the cost effectiveness of CSII in managing TIDM in childhood or in adolescents has not been determined; the annual cost of consumables required to treat a patient on MDI receiving four injections a day is £227.

A large number of observational studies have been published reporting the effect of CSII on a range of clinical parameters and have generated conflicting data. In some studies glycaemic control improves (10-14) while in others there is no change in $HbA1_c$ or no difference in glycaemic excursions (15-18). The effect of CSII on Body Mass Index (BMI) has been reported as unchanged (11,14,17,19), increased (10,17) or reduced (12,20).It is often reported anecdotally that the principle indication for CSII therapy is improved quality of life (QoL) however, even in this domain data are equivocal (12,20).

A recent meta-analysis (21) of randomised controlled studies investigating the outcomes of children with TIDM treated with CSII compared to those treated with MDI found six studies suitable for inclusion of which three included only preschool children. In total 165 children and young people were included in these studies of which 78 children were aged more than 5 years. The largest number of children included in a single study was 32. In general the period of observation was brief: seven months or less in five studies. In studies reporting data from children aged more than 5 years the period of observation ranged from four to seven months. Two of the three studies reporting children in this age group came from the same centre in Israel (15,22) and the third study came from Yale University School of Medicine(19).

Four studies reported data on change in $HbA1_c$ from baseline to the end of the study. A statistically significant difference in $HbA1_c$ between children treated with CSII and MDI was demonstrated at three months. Meta-analysis of data beyond three months was not possible due to study heterogeneity. Individual data at study completion were available in five studies of which there was no significant difference in glycaemic control between treatment groups

in three. In one study $HbA1_c$ was significantly lower in children treated with CSII, while children treated with MDI achieved a significantly lower $HbA1_c$ in another (15).

A number of other outcomes were reported in this meta-analysis. Analysis of insulin requirement was included in three studies and lower doses of insulin were reported in children treated with CSII. Two studies reported Body Mass Index (BMI) of which one reported no significant difference in BMI percentile, while another reported significantly higher BMI Standard Deviation Score (SDS) in those treated with MDI. Quality of life measures were included in four studies including 98 patients. Two of these four studies included children aged 5 years and above (n=55) and significantly better QoL was reported in children treated with CSII in one study, while another reported no difference between treatment groups.

A more recent study, not included in this meta-analysis, has recently been published (23) . In this study thirty eight children aged four to sixteen years were recruited. After a three month run in period of MDI children were randomised to either continue on MDI or to change to CSII and data were collected for a further 3.5 months. Between 3.5 and 14 months all children were treated with CSII. Glycaemic control and QoL significantly improved during the run in period. During the randomisation period there was no significant difference in QoL or $HbA1_c$ between treatment groups.

These studies demonstrate that that CSII is a safe and effective treatment in children and adolescents with TIDM, however there are two common flaws in each of them: the patient populations are small and the periods of observation are short. The introduction of CSII is preceded by a period of intensive education followed by a period of close support from healthcare professionals. The design of these studies does not allow one to discriminate whether the primary influence on the outcome measures is an increase in education and support or a direct effect of the method of insulin delivery. If the primary influence on glycaemic control is unrelated to the mode of insulin delivery it may be more appropriate to invest in diabetes healthcare professionals with the potential of influencing the long term outcomes of a much larger number of patients. However, if CSII does improve glycaemic control or other measures of health and well-being the additional cost of this therapy is likely to be offset by a reduction in the complications of diabetes.

In summary, there is an urgent need to critically appraise the benefit of CSII therapy to ensure that resources invested to reduce the risk of complications of TIDM are appropriately targeted. The increasing prevalence of TIDM in childhood coupled with poor glycaemic control, will present a major economic burden to the NHS in the near future and poses a significant threat to the QoL of the individual.

2.2 Rationale

This study is designed to provide an evidence base to inform future NHS investment in health care services for children and young people with TIDM. The role of intensive insulin therapy in optimizing glycaemic control and thereby reducing the risk of vascular complications of TIDM is unquestioned however the optimal way in which to achieve this and the cost effectiveness of the tools currently available is unknown. This study will compare two methods of insulin delivery during childhood and adolescence to identify which facilitates superior glycaemic control, and examine the impact of treatment modalities on other predictors of vascular complications of TIDM, adverse events and QoL.

The recruitment of children to a randomised controlled trial of two insulin delivery systems is likely to be unsuccessful in children and young people with established diabetes in whom preference is likely to exclude them from randomisation or strongly influence the successful use of the therapy. For this reason this study will recruit patients at the time of diagnosis.

However, we are aware of the potential difficulties recruitment at the time of diagnosis poses. For this reason we propose an inbuilt pilot study to test the feasibility of recruiting a cohort of patients who are representative of the background population of children and young people with diabetes.

2.3 Objectives

Inbuilt pilot study

Primary Objective:
To acquire an understanding of the acceptability of randomisation to MDI or CSII at diagnosis of TIDM in children and young people.

Secondary Objectives:
To define the characteristics of patients who consent and those who do not consent to randomisation.

To use the data as an internal pilot to check the size of the standard deviation used in the sample size calculation of the full study.

Full study

Primary Objective:
To compare the glycaemic control assessed by $HbA1_c$ at 12 months after diagnosis in children and adolescents receiving CSII with those receiving MDI.

Secondary Objectives:

To compare the following:
Clinical effectiveness of children and adolescents receiving CSII with those receiving MDI.

The safety of children and adolescents receiving CSII with those receiving MDI.

Growth of children and adolescents receiving CSII with those receiving MDI

The quality of life of children and adolescents receiving CSII with those receiving MDI

Cost effectiveness of providing insulin to children and adolescents by CSII compared to MDI.

2.4 Potential Risks and Benefits

2.4.1 Potential Risks

When potentially eligible children and young people are identified, their parent or person with parental responsibility will be approached by the investigator, or a designated member of the investigating team, during which an opportunity will be given to understand the objectives, risks and inconveniences of the trial. Although insulin glargine is unlicensed for use in children aged less than 2 years, the insulin analogues and devices under investigation are those routinely used in children with TIDM. We do not anticipate any increase in adverse events associated with the use of either delivery method over and above the complications normally seen in routine clinical care.

2.4.2 Known Potential Benefits

The NICE guideline recognises that intensive therapy (MDI or CSII) offers greater potential to optimise glycaemic control and minimise long term complications than conventional insulin regimes of twice daily pre-mixed insulin. For this reason, NICE recommends that all children and young people should be offered intensive therapy from the time of diagnosis in the form of MDI. Children aged 12 and above should have access to CSII where $HbA1_c$ levels below 8.5% cannot be achieved without disabling hypoglycaemia despite high levels of care and children under 12 years should have access to CSII from diagnosis where MDI is considered impractical or inappropriate. Participants recruited to this study will be randomly allocated one of these two intensive therapies (CSII or MDI). Children recruited to the study aged 12 years or more will have access to CSII at diagnosis, a treatment currently not recommended at diagnosis by NICE. However, in participants aged less than 12 years of age participation will eliminate patient and family choice of insulin delivery system.

There are currently no data from which patients, parents or health care professionals can draw to inform their selection of insulin delivery device. Although the element of choice will be removed from participants recruited to this study, we believe this is acceptable given that there is no evidence demonstrating the superiority of either treatment. All data generated by this study will be closely monitored by the Independent Data and Safety Monitoring Committee (IDSMC). All patients and clinical teams will be advised of the findings and a decision will be made locally whether or not to offer the superior treatment on the basis of the individual's glycaemic control.

3 SELECTION OF CENTRES/CLINICIANS

Centres meeting the following criteria will be selected for the study. The SCIPI trial will take place in paediatric diabetes outpatient clinics/ ward or A&E unit (centres) in the UK. The study will be coordinated through the Medicines for Children Clinical Trial Unit (MC-CTU), the Diabetes Research Network (DRN) and the Comprehensive Clinical Research Network (CCRN). Study centres will be initiated once all local R&D approval and study-specific conditions (e.g. training requirements) have been met, and all necessary documents have been returned to MC-CTU. Initiation meetings will cover the requirements outlined in CTRC SOPs TM017 and TM018.

3.1 Centre/Clinician Inclusion Criteria

Any paediatric diabetes outpatient clinic/ ward or A&E unit based within NHS can participate in the trial provided the following criteria's are met:

a. Experience in managing 10 or more patients treated with CSII and currently offer MDI regimen;
b. Multidisciplinary team including paediatric consultant, dietician, diabetes nurse and other medical staff all trained in the care of children with diabetes;
c. Local R&D approval;
d. Signed non-commercial agreement between the centre and the sponsor;
e. Completion of the 'Signature and Delegation Log';
f. Receipt of evidence of completion of (c), (d)and (e) by the MC-CTU.

3.2 Centre/Clinician Exclusion Criteria

a. Not meeting the inclusion criteria listed above

4 TRIAL DESIGN

This is an open labelled 2-arm multi-centre randomised controlled trial comparing continuous subcutaneous insulin infusion (CSII) with multiple daily injections (MDI) of insulin in 316 children and young people aged 7 months to 15 years who have been newly diagnosed with type I diabetes mellitus. Patients should be approached and consented as soon after diagnosis as possible. Participants will be randomised in a 1:1 ratio to either of the two treatments (MDI or CSII pumps). Training will be given to all the participants and their parents regarding use of the intervention(s) and randomised treatment will be started within 14 days of diagnosis. The study includes an internal pilot with a sample size of 30 participants to estimate the rate of consent to randomisation and to define the characteristics of recruited participants.

4.1 Pilot Study

An inbuilt pilot study (n=30) will test the feasibility of recruitment to this study protocol. The full study (n=316) will open if the following criteria are met:

a. Fifty percent or more patients who are eligible and are invited to participate in the pilot study are successfully recruited;
b. Demographic characteristics like age, ethnicity, gender and deprivation score are not considered to be significantly different in the group of patients who are recruited compared to those who decline. These characteristics are significantly associated with glycaemic control (24-26).

4.2 Full Trial

Primary Endpoint
Glycaemic control ($HbA1_c$) 12 months after diagnosis.

Secondary Endpoint(s)

a. Percentage of participants in each group with $HbA1_c$ <6.5%
b. Incidence of severe hypoglycaemia
c. Incidence of diabetic ketoacidosis
d. Change in BMI SDS
e. Height
f. Insulin requirements (units / kg /day)
g. PedsQL
h. Incremental cost per QALY gained
i. Partial remission

On or near completion of the 12 month study permission will be sought from participants and their carers for ongoing submission of routine clinical data to the research team for a further nine years to enable long term outcomes to be reported. This will include:

a. $HbA1_{c;}$
b. Insulin treatment regimen;
c. BMI;
d. Insulin dose (units/kg/day);
e. Serious adverse events;
f. Complications of TIDM.

5 STUDY POPULATION

5.1 Inclusion Criteria

Patients with the following characteristics will be eligible for inclusion in the trial:

a. Newly diagnosed TIDM using standard diagnostic practice (27);
b. Age 7 months – 15 years (inclusive);
c. Parent/legal representative of the patient are willing to give consent for the study;
d. Parent/legal representative of the patient are able to comply with the treatment regimen and study visits.

5.2 Exclusion Criteria

Patients with the following characteristics will be excluded from the trial:

a. Treated previously for diabetes;
b. Haemoglobinopathy;
c. Co-existing pathology conditions likely to affect glycaemic control, e.g. cystic fibrosis;
d. Psychological or psychiatric disorders, e.g. eating disorder;
e. Receipt of medication likely to affect glycaemic control, e.g. systemic or high dose topical corticosteroid or growth hormone therapy;
f. Allergy to a component of insulin aspart or insulin glargine;
g. Have a sibling with existing TIDM
h. Known thyroid condition in a non euthyroid state and;
i. Known Coeliac disease unable to maintain a gluten free diet.

5.3 Participant Transfer and Withdrawal

In consenting to the trial, participants are consenting to trial treatment, follow-up and data collection. If voluntary withdrawal occurs, the participant (or parent/legal representative) should be asked to allow continuation of scheduled evaluations, complete an end-of-study evaluation, and be given appropriate care under medical supervision until the symptoms of any adverse event resolve or the participant's condition becomes stable. Follow-up of these participants will be continued through the research nurse and the lead investigator at each centre and, where these are unsuccessful, through the child's local paediatric diabetic service or via their GP.

The research team plans to work with the DRN and the CCRN to enable ongoing observation of participants as they move from paediatric to adult health care services. This ongoing observation will be the focus of a separate application for funding.

5.3.1 Participant Transfers

For the participants moving from the area, every effort should be made for the participant to be followed-up at another participating trial centre and for this trial centre to take over responsibility for the participant or for follow-up at the local paediatric diabetic service or via their GP.

A copy of the participant case report forms (CRFs) and other study related documents should be provided to the new centre. The participant (or parent/legal representative) will have to sign a new consent form at the new site, and until this occurs, the participant

remains the responsibility of the original centre. The MC-CTU should be notified in writing of participant transfers.

5.3.2 Withdrawal from Trial Intervention

Participants may be withdrawn from treatment for any of the following reasons:

a. Parent/ legal representative (or, where applicable, the participant) withdraws consent.
b. Intercurrent illness preventing further treatment.
c. Any change in the participant's condition that justifies the discontinuation of treatment in the clinician's opinion.

If a participant wishes to withdraw from trial treatment, centres should nevertheless explain the importance of remaining on trial follow-up, or failing this, of allowing routine follow-up data to be used for trial purposes. Generally, follow-up will continue unless the participant explicitly also withdraws consent for follow-up (see section 5.3.3).

5.3.3 Withdrawal from Trial Completely

Participants are free to withdraw consent at any time without providing a reason. Although a participant or parent/legal representative is not obliged to give his/her reason(s) for withdrawing prematurely from a trial, the investigator should make a reasonable effort to ascertain the reason(s), while fully respecting the participant's rights. Participants who wish to withdraw consent for the trial will have anonymised data collected up to the point of that withdrawal of consent included in the analyses. The participant will not contribute further data to the study and the MCRN should be informed in writing by the responsible physician and a withdrawal CRF should be completed. Data up to the time of withdrawal will be included in the analyses unless the participant explicitly states that this is not their wish.

If the participant explicitly states that they wish to withdraw from the trial completely and that they do not want their data to be included in the final analyses, please contact the SCIPI trial coordinator to request a Participant Data Withdrawal CRF.

6 ENROLMENT AND RANDOMISATION

6.1 Screening

All patients who are newly diagnosed with TIDM aged 7 months -15 years (inclusive) at the study centres will be screened to identify potentially eligible participants for the study.

Potentially eligible participants (i.e. those that meet the eligibility criteria listed in section 5) and their parent or legally acceptable representative will be invited to participate in the study and provided with the patient information sheet and consent form. The family will be allowed sufficient time to discuss the trial and decide whether to consent to trial entry (see section 11.3 for consent procedures). Providing information, discussing the study and obtaining informed consent should occur as close to the time of diagnosis as possible, ideally between the time of diagnosis (Day 0) and Day 5. Once consented, baseline data should be collected, the participant should be randomised (see section 6.2 and 6.3) and the randomised treatment should be started within 14 days of diagnosis (see section 7).

A ‘Screening log’ will be maintained of all the patients who undergo screening regardless of whether they decide to participate in the study. Reasons for not being eligible and timelines for providing information and approaching the patient for consent will be recorded. Reasons for declining to participate will be asked routinely but it will be made clear that patients, parents or carers do not have to provide a reason unless happy to do so.

6.2 Baseline

Once consent has been obtained from the parent or legally acceptable representative, and assent by the child where appropriate, the delegated member of the research team e.g. Research Nurse (RN[1])/consultant should complete the randomisation and baseline CRFs. For a summary of CRF content see table 1 in section 8.1

6.3 Randomisation

Participants will be randomised to receive either MDI or CSII (in a 1:1 ratio) once:

a. Eligibility criteria has been fulfilled;
b. Fully informed written proxy consent has been obtained;
c. Baseline assessments have been completed.

Participants will be randomised using a secure (24-hour) web based randomisation programme controlled centrally by the MC-CTU. Personal login username and password, provided by the MC-CTU, will be required to access the randomisation system. When consent eligibility and participant age have been confirmed the participant treatment allocation and a unique study number (randomisation number) will be displayed on a secure webpage and an automated email confirmation will be sent to the authorised randomiser, Principal Investigator (PI) and trial coordinator. It is the responsibility of the PI or delegated research staff to inform the pharmacy department at their centre prior to randomisation to ensure there is sufficient supply of the study treatments. For further details on randomisation see section 9.2.

---

[1] When RN is referred to in this protocol it means either the diabetes research nurse or member of the research team who has been delegated that duty.

Randomisation: web access http://www.scipitrial.org.uk/rand

If there are any problems with randomisation please contact the MC-CTU

(from Monday to Friday between 9:00 to 17:00 excluding bank holidays)
helpdesk on: 0845 68 00 951 or contact the trial coordinator
Or via email on helpdesk@mcrnctu.org.uk or scipi@liverpool.ac.uk

Randomisation backup envelopes will be used in case of failure of randomisation system outside MC-CTU working hours

Research staff will be trained to use the web based randomisation system during the initiation process. *After research staff are trained they will be issued with personal login and password details.*

Centres will be provided with emergency back-up randomisation envelopes to be used in the event of a failure occurs outside MC-CTU office hours or the problem cannot be resolved in a reasonable timeframe. In the event that emergency back-up envelopes are required, the randomising person will select the next sequentially numbered, opaque, pressure-sealed envelope that will give the randomisation allocation. The envelope will be similar to those used for pay slips, which cannot be viewed without fully opening and their construction is resistant to accidental damage or tampering. Page 1 of the randomisation envelope containing information on the allocation should be returned to the MC-CTU in a pre-paid envelope, and pages 2 & 3 of the randomisation envelope can be inserted into the patient's medical records.

The RN will check to ensure that the correct number of randomisation envelopes are present, that they are intact and that the sequential numbering system is maintained. Any discrepancies should be immediately reported to the MC-CTU.

7 TRIAL TREATMENT/S

7.1 Introduction

This RCT compares two methods of insulin delivery via CE-marked devices used for their intended purpose (CSII pumps versus MDI using appropriate insulin pen devices) in children from 7 months to 15 years of age. The insulin glargine and insulin aspart are the most commonly used insulin's in British paediatric practice

Insulin aspart is an insulin analogue licensed for the treatment of TIDM in adults, adolescents and children aged 2 to 17 years but should only be used in children under 2 years under careful supervision; however the rapid onset and offset of action of this insulin make it particularly attractive in the management of young children. For this reason it is widely used in young children with TIDM.

The safety and efficacy of insulin glargine (Lantus) have been established in adolescents and children aged 2 years and older. Insulin glargine has not been studied in children below the age of 2 years; however, the use of insulin glargine in this age group has been associated with a reduction in hypoglycaemia and improved metabolic control (28). For these reasons it is widely used in intensive treatment regimens in British paediatric practice.

After randomisation, participants will be treated either with MDI - or CSII treatment. Randomised treatment should begin within 14 days of diagnosis. The clinician should ensure that the duration between obtaining consent, performing baseline assessments, randomisation and the start of trial treatment does not impact on the well-being of the participant. Any medication given between diagnosis and the start of the randomised treatment to stabilise the participant should be recorded in the baseline CRF. Assessments that should be carried out prior to the start of the randomised treatment are listed in table 1 in section 8.1.

7.2 Education for both arms

At entry to the study, all participants will complete a structured educational program delivered to participants and their families in accordance with the standards of the International Society for Paediatric and Adolescent Diabetes (29).
Participants and their families will be educated in:

a. Type I diabetes
b. The use and administration of Insulin
c. Hyperglycaemia and correction doses
d. Hypoglycaemia symptoms and treatment
e. Exercise
f. Sick Day rules
g. Carbohydrate counting
h. The benefits of maintaining optimal glycaemic control for long term health.
i. Blood glucose monitoring

All the participants will be trained on the use of MDI regimen and use of Expert glucometer; whilst participants undergoing CSII treatment will also be trained in the use of CSII pumps.

Dietetic Education
Diabetic education will be organised to suit individual needs of the participant and family. The dietician will meet the participant and their family to assess their diet and educate them in carbohydrate counting.

7.3 Formulation, Packaging, Labelling, Storage and Stability

Treatment	Insulin Analogues(with trade names) and Devices		Supplier/ Manufacturer	Route of Administration
MDI Treatment	Insulin Analogues	Insulin Glargine (Lantus 100 units/ml solution for injection)	Sanofi Aventis	Subcutaneous Injection
		Insulin Aspart (Novorapid 100 U/ml solution for injection)	Novo Nordisk	
		Insulin Detimir. Levemir Cartridge 100 units/ml - Penfill, Levemir Pre-filled Pen 100 units/ml - FlexPen and InnoLet	Novo Nordisk	
	Devices	Insulin Pen Injection Device	Novo Nordisk or supplier as per local policy	
		Expert Glucometer	Roche Pharmaceuticals via MC-CTU	
CSII Treatment	Insulin Analogues	Novorapid 100 U/ml solution for injection (Insulin Aspart)	Novo Nordisk	Subcutaneous Infusion
	Devices	CSII Insulin Pumps	Roche Pharmaceutical via MC-CTU or, in exceptional circumstances supplier as per local policy e.g. medtronic pumps	
		Glucometer (synchronised combo with pump)	Roche Expert meter	

Participating centres should notify the MC-CTU of the manufacturer and model of MDI, CSII pumps and glucometers to be used in the trial at their site prior to opening to recruitment.

All Insulin analogues should be stored as per the Summary of Product Characteristics (SPCs), whilst insulin devices should be stored and handled as per the manufacturer's instructions for use. Storage should also be in line with local practice. Please refer to the reference SPCs and manufacturer instructions for use provided as separate documents to this protocol.

SCIPI is a pragmatic trial that uses market authorised products (insulin analogues). For this trial, there is an exemption from clinical trial labelling in accordance with regulation 46 of SI 2004/1031. Therefore a pharmacy label is sufficient when the product is dispensed against a prescription.

IMPs will be dispensed by hospital and community pharmacies as they would normally in clinical practice. It is the responsibility of the PI to ensure that the GP prescribes the remainder of any trial treatment not dispensed by the hospital pharmacy.

7.4 Preparation, Dosage and Administration of Study Treatment/s

The insulin will be administered using the following insulin delivery systems. All variations allowed in the trial design are CE-marked medical devices used in accordance with the manufacturer's instructions for their intended purposes.

For MDI treatment: Insulin aspart and insulin glargine will be delivered subcutaneously using an insulin pen injection device in accordance with manufacturer instructions for use. Participants will be given insulin glargine (long acting analog) once or twice daily according to their needs and boluses of insulin aspart (short acting analog) when 10g or more of carbohydrate are consumed.

Pens/consumables for participants allocated to multiple daily injections will be supplied in line with normal clinical practice.

Within this clinical trial the use of glargine is preferred for MDI administration of insulin. However, if local site policy pertains use of the insulin Detemir then the MC-CTU should be informed of each occurrence.

CSII treatment: Insulin aspart will be administered using CSII insulin pumps (Refer to Insulin Pump Manual Guide). Participants will be given insulin aspart using basal insulin infusion with bolus doses of insulin aspart when 5g or more carbohydrate is consumed.

For Roche supplies, the pump, consumables and glucometers will be supplied at the point of randomisation from the existing stock (supplied during setup) held at each investigator site. For participants allocated Roche pumps only, the RN will register participants with Roche following the guidance specified in the SCIPI Intervention Trial supplies Flowchart. This will ensure that consumables are supplied directly to participants when needed, via the Roche careline process.

Medtronic / Omnipod pumps and consumables will be supplied in line with normal clinical practice.

Starting Dose Calculations:
Total daily starting dose of insulin will be calculated from body weight. In pre pubertal subjects 0.5units/kg body weight/day with 50% of calculated dose given either as insulin glargine injection into the anterior-lateral aspect of the thigh, arm, abdomen or the upper outer quadrant of the buttocks (MDI) or as a continuous 24hour infusion (0.5x kg body weight, ÷ 2 ÷ 24 = hourly rate) (CSII). The remaining 50% will be given as 3 divided pre-prandial doses at meal times. If the doses are not equal more insulin will be given before breakfast and the evening meal than at lunch time to account for diurnal variation in insulin

sensitivity. In pubertal participants the initial dose calculation will be based on 0.7units/kg body weight /day.

It is recommended that blood glucose readings should be undertaken at least four times a day, pre breakfast, pre midday meal, pre evening meal and pre supper/bed.

7.5 Dose Modifications

Correction dose will be calculated according to the ‘100’ rule (30). Participants will be managed as per local treatment protocols. Insulin doses will be titrated according to home blood glucose readings, as per local routine clinical advice. The diabetes clinical team will be in contact with families to support insulin dose titration adjustment according to participant need. Telephone contact will also be an opportunity for the diabetes research nurse to provide support and education regarding the management of TIDM. The frequency of telephone contact with their local clinical service will be logged. All participants and their parents will also have 24 hour telephone access for support and advice throughout the study as is standard practice. Participants on CSII treatment will also have 24*7 access to the pump manufacturer’s helpline for assistance for technical problems relating to the pump.

7.6 Accountability Procedures for Study Treatment/s

The centre should have systems in place to ensure the following and will report any problems to the MC-CTU:

a. A record of deliveries and administration of insulin analogues and devices;
b. A system in place that allows for the retrieval of defective products;
c. That there are enough insulin analogues and devices within their shelf life assigned to be used in the study and that they are given to the participants free of charge;
d. That the insulin analogues and devices are stored where they are readily accessible to the designated study staff responsible for randomisation and administration of treatment;
e. Insulin analogues are stored and used in compliance with the SPC and protocol requirements;
f. The CSII pumps and the insulin pen injection device are stored and used in compliance with the device manual guide, leaflet instructions and protocol requirements;
g. The disposal of treatments when the shelf life expires according to local policy and arrange resupply where appropriate;

Once the trial has closed at a centre all used CSII pumps will remain the property of the recruiting centre. Any used CSII pumps purchased via the sponsor/MC-CTU will be returned to the sponsor/MC-CTU.

7.7 Assessment of Compliance with Study Treatment/s

In order to assess compliance with the trial protocol; insulin usage data will be collected from the participants as described in section 8.2.2.

7.8 Concomitant Medications/Treatments

Treatment is contraindicated for patients who are hypersensitive to the insulin analogues or to any of the excipients. Patients on medications most likely to affect glycaemic control e.g. systemic or high dose topical corticosteroid or growth hormone therapy will be excluded from the trial. The dose and name of all prescribed concomitant medications should be documented on the Concomitant Medication CRF. The PI or delegated research member should reassess concomitant medications at each trial visit. Please refer to the reference SPC for a list of drugs that may affect treatment.

7.9 Co-enrolment Guidelines

To avoid potentially confounding issues, ideally patients should not be recruited into other trials. Where recruitment into another trial is considered to be appropriate and without having any detrimental effect on the SCIPI trial this must first be discussed with the coordinating MC-CTU who will contact the Chief Investigator, Dr Joanne Blair.

8 ASSESSMENTS AND PROCEDURES

Participating centres will be expected to maintain a file of essential trial documentation (Site File), which will be provided by the MC-CTU, and keep copies of all completed case report forms (CRFs), participant completed questionnaire and the treatment diaries for the trial. Data collection will use a combination of paper CRFs (with no carbon copies) and electronic data collected retrospectively from the CSII pumps and glucometers (see section 13.2 for details on the data capture methods).

All paper CRFs should be completed as described in section 13.2 by personnel named on the delegation log as authorised to do so, usually the RN, and returned to the MC-CTU within 7 days of contact with patient for follow up, unless stated otherwise.

Participant details including initials, date of birth, NHS number, postcode and randomisation number will be reported on the consent form, separate to clinical data. Once written informed consent has been obtained from the parent or legally acceptable representative, the RN will collect baseline characteristics using the baseline CRF and the patient will be randomised and followed-up in the trial. For screening and randomisation procedures refer to section 6. For details of procedures associated with trial treatments refer to section 0. For a summary of CRF content see table 1 in section 8.1.

8.1 Schedule for Follow-up

The study duration of each participant (including follow-up period) is one year from the date of randomisation. All participants will be reviewed at the diabetes outpatient clinic every three months as per standard clinical practice and will have four follow up visits at 3-, 6-, 9- and 12- months from randomisation. Participants will have a flexible window of ± 15 days to report for each follow up visit.

Table 1: Trial Assessments

Procedures		Baseline		Follow Up<br>Scheduled three monthly clinic visit from time of randomisation			
		Diagnosis	Prior to start of treatment				
		**T0 = Randomisation** (randomisation will be made within 14 days of diagnosis)		**(T0+3 months)**	**(T0+6 months)**	**(T0+9 months)**	**(T0+12 months)**
**Assessment of Eligibility Criteria**		X					
**Signed Consent Form**		X[1]					
**Randomisation**		X					
**Review of Concomitant Medications**		X		X	X	X	X
**Review of insulin use (Insulin Requirements)**	**Treatment Diaries**			X	X	X	X
	**Prescriptions**			X	X	X	X
	**CSII pumps download**			X	X	X	X
**Blood Glucose measurement**		X[2]		X	X	X	X
**Blood pH measurement**		X					
**Demographics**		X					
**Study Intervention**			X[3]				
**Physical Exam**	**Height**	X		X	X	X	X
	**Weight**	X		X	X	X	X
	**Injection Sites**			X	X	X	X
	**Symptom-Directed**			(X)	(X)	(X)	(X)
**Assessment of Related Adverse Events**	**Incidence of severe hypoglycaemia**			(X)	(X)	(X)	(X)
	**Incidence of ketoacidosis**			(X)	(X)	(X)	(X)
	**Other Adverse Events**			(X)	(X)	(X)	X)
	**Related to diabetes**			(X)	(X)	(X)	X)
**Clinical Laboratory**	**$HbA1_c$ analysis[4]**	X		X	X	X	X
	**Other routine tests[5] (e.g. Chemistry, Haematology, Urinalysis)**	(X)		(X)	(X)	(X)	(X)
**Questionnaires**	**Participant completed PedsQL**				(X)		(X)
	**Parent completed PedsQL**				X		X

Procedures		Baseline		Follow Up<br>Scheduled three monthly clinic visit from time of randomisation			
		Diagnosis	Prior to start of treatment				
		**T0 = Randomisation (randomisation will be made within 14 days of diagnosis)**		**(T0+3 months)**	**(T0+6 months)**	**(T0+9 months)**	**(T0+12 months)**
	**Participant completed Health Utilities Index Mark 2 and 3**		(X)	(X)	(X)	(X)	(X)
	**Parent completed Health Utilities Index Mark 2 and 3**		X	X	X	X	X
**Resource Use**	**RN completed CRF**		X	X	X	X	X

(X) – As indicated/appropriate.

1- Providing information, discussing the study and obtaining informed consent should occur as close to the time of diagnosis as possible, ideally between the time of diagnosis and Day 5 (diagnosis date +5).

2 - Measurement of blood glucose will be done by a glucose test at the time of diagnosis and measured as per local policy and the results recorded from patient medical records. It is measured by glucometer at remaining time points (refer section 8.2.3).

3 - Study Intervention –Randomised treatment should be commenced within 14 days of diagnosis

4– Hb1Ac will be collected and analysed according to local clinical practice and a sample sent for central analysis.

5 - Routine clinical tests will be conducted as part of routine clinical management and where appropriate results recorded from patient medical records.

8.2 Procedures for assessing Efficacy

Efficacy of the study treatments will be measured throughout the period of the study using both objective and subjective measures.

8.2.1 HbA1c

A glycosylated haemoglobin (HbA1c) level indicates the mean concentration of glucose in the participant's body over the preceding three month period. A blood sample (finger prick; approximately 3 drops) from all the participants will be collected at each site by the diabetes clinical care team for the analysis of HbA1c (as primary end point of the trial) prior to start of the randomised treatment then at 3, 6, 9 and 12 months.

The blood sample will be collected and analysed in line with local hospital laboratory standards.

Additionally, a blood sample will be collected in a tube identified by initials, study unique number and date/time of sample. The sample will be sent securely to a central clinical pathology laboratory at Alder Hey Children's NHS Foundation Trust for analysis to check standardisation of results from local hospital laboratories.

8.2.2 Insulin requirements

Participants receiving CSII
Insulin usage data will be downloaded from the CSII pumps by the RN or the delegated person at 3-, 6-, 9- and 12- month study visit and forwarded electronically to the MC-CTU (guidance detailing the process will be provided to centres in the investigator site files. All the participants will record the insulin doses in the treatment held diaries used as part of routine practice.

Participants receiving MDI
The RN will review the treatment diaries at each follow up visit and transcribe information from the diaries to the insulin usage CRF, which will be forwarded to the MC-CTU. At follow up visit the RN shall also contact the participant's GP to acquire the quantity of insulin prescribed for the participant. This will be compared to the quantities recorded by participants to guard against significant under-reporting. Any circumstances of non-compliance should be recorded on the participant's insulin usage CRF.

8.2.3 Blood glucose measurements

At the time of diagnosis, blood glucose levels of all the participants will be measured according to local policy. At the remaining time points blood glucose will be measured using a glucometer (see section 7.3 for glucometer supply details). Data from the glucometers will be downloaded by the diabetes research nurse or the delegated person at 3-, 6-, 9- and 12-month study visit and forwarded electronically to the MC-CTU (guidance detailing the process will be provided to centres in the investigator site files).

8.3 Procedures for Assessing Safety

8.3.1 Adverse events:

Participants will be monitored for treatment related adverse events (AEs) and treatment related serious adverse events (SAEs) (see section 10 for details). They will be issued with a diary to document home episodes of severe hypoglycaemia and diabetic ketoacidosis. Treatment diaries and telephone logs will be assessed at each study visit for any treatment related AEs and related SAEs.

For this study we will also monitor adverse events related to the disease under study.

In addition to self-reporting, local hospital databases will be interrogated at each clinical assessment to ascertain whether the subject has been treated for a related SAE in the preceding three months. Related AEs (including incidences of severe hypoglycaemia and diabetic ketoacidosis) and related SAEs will be reported as described in section 10.6.

8.3.2 Body Mass Index (BMI):

BMI of all the participants will be assessed at diagnosis, prior to start of treatment, then at 3-, 6-, 9- and 12- month study visit. Height will be measured using a Harpenden stadiometer and weight will be measured using electronic scales (as per standard practice).

BMI will be derived from the formula:-

BMI = weight (kg) / height (m^2)

Standard deviation scores (SDS) for BMI will be derived from the UK 1990 standards (31).

8.4 Other Assessments

Quality of Life (PedsQL; see section 8.4.1) and Health Status instrument (HUI2/3; see section 8.4.2) have been combined into age appropriate questionnaire booklets for ease of administration.

Time Points	Children Aged	Questionnaire booklet administration completed by	
		Parent/carer	Child
Baseline,	3 years – 11 years	Booklet 3 –15 years (HUI2/3)	-
	12 years -15 years	Booklet 3-15 years (HUI2/3)	Booklet 12-15 years (HUI2/3)
3 and 9 months	3 years – 11 years	Booklet 3 –16 years (HUI2/3)	-
	12 years -16 years	Booklet 3-16 years (HUI2/3)	Booklet 12-16 years (HUI2/3)
6 and 12 months	2 years	Booklet 2 years (PedsQL)	-
	3 years-4 years	Booklet 3-4 years (PedsQL and HUI2/3)	-
	5 years – 7 years	Booklet 5-7 years (PedsQL and HUI2/3)	Booklet 5-7 years (PedsQL)
	8 years – 11 years	Booklet 8-12 years (PedsQL and HUI2/3)	Booklet 8-11 years (PedsQL)
	12 years	Booklet 8-12 years (PedsQL and HUI2/3)	Booklet 12 years (PedsQL and HUI2/3)
	13 years -16 years	Booklet 13-16 years (PedsQL and HUI2/3)	Booklet 13-16 years (PedsQL and HUI2/3)

8.4.1 Quality of Life

Quality of Life (QoL) will be assessed using validated diabetes specific QoL (PedsQL) questionnaire instruments (32) at 6- and 12-months after diagnosis. Children and young people aged 5-7 years, 8-12 years and 13-16 years will use developmentally appropriate versions and the parental version will be used for all age groups (32). The instrument has been reported as offering the optimal clinical utility when compared to other diabetes-specific instruments in this age group (33).

8.4.2 Health Economics analysis:

The economic analysis will adopt the perspective of the NHS by measuring healthcare resource use and costs including those associated with both technologies, CSII (purchase, maintenance and use of disposables) and MDI; those associated with adverse events, any necessary investigations, procedures, treatments and hospitalisations; insulin and other medicines; and contact with health professionals.

Data collection

- Resource use will be collected prospectively within participants' CRFs. Participants' use of other services will be collected by means of a health resource use questionnaire collected by the RN in interview with the participant prior to start of randomised treatment (for the previous 3-months) then at 3-, 6-, -9 and 12-months study visits.
- Data from Patient Administration Systems will be requested. The trial health economist will contact the Finance departments, Information Technology and Patient Administration Departments of each recruiting centre to inform them of SCIPI, and that a future request will be made for data on trial participants' healthcare resource use. 12-months after randomisation of the last patient at each recruiting centre, the trial health economist will contact the Finance departments of each centre, and submit a request (via the MC-CTU, to maintain patient anonymity) for: Ward name; ward speciality (e.g. paediatric, paediatric ICU, etc.); the average cost per bed day on the ward; and the financial year the costs refer to. The health economist will also contact the Information Technology or Patient Administration Departments of each centre and submit a request (via the MC-CTU, to maintain patient anonymity), for: ward name; ward speciality (if possible); start date on the ward; end date on the ward; number of occupied bed days on the ward.
- Unit cost data will be obtained from a variety of sources, including routine hospital data (NHS reference costs) and nationally published data (34).
- Health utilities will be assessed prior to start of the treatment then at 3-, 6-, -9 and 12-months study visits. This will be accomplished by administering the Health Utilities Index Mark 2 and 3 -HUI23P1En.15Q questionnaire to the parents/carers of all the participants and HUI23S1EN.15Q questionnaire to the participants of 12 years and over. The HUI2/3 has been validated for use in children (35) and is the preferred instrument for this purpose (36). The six attributes of the HUI2/3 (sensation, mobility, emotion, cognition, self-care, and pain) will be summarized into a single UK-derived preference-based utility score (37).

8.5 Loss to Follow-up

If any of the trial participants are lost to follow up, contact will initially be attempted through the PI or delegated research staff at each centre. If the lead investigator at the trial centre is not the participant's usual clinician responsible for their specialist care then follow up will also be attempted through this latter clinician. Where these attempts are unsuccessful, the participants GP or the child's local diabetic service will be asked to contact the participant or the participant's family to provide follow up information to the recruiting centre. Consent will be sought to contact the participants GP. Wherever possible, information on the reason for loss to follow up will be recorded.

8.6 Trial Closure

The end of the trial is defined to be the date on which data for all participants is frozen and data entry privileges are withdrawn from the trial database. However, the trial may be closed prematurely by the Trial Steering Committee (TSC), on the recommendation of the Independent Data and Safety Monitoring Committee (ISDMC).

9 STATISTICAL CONSIDERATIONS

9.1 Introduction

A separate and full Statistical Analysis Plan (SAP) will be developed prior to the final analysis of the trial. The SAP will be agreed by the Trial Steering Committee (TSC). The main features of these planned statistical analyses are included here in the protocol.

9.2 Method of Randomisation

Participants will be remotely randomised in a 1:1 ratio using a web service. The randomisation code list will be generated by a statistician using block randomisation with random variable block size method at the MC-CTU. Participants will be randomised to either MDI or CSII treatment in a ratio of 1:1 and will be stratified by centre and age.

9.3 Outcome Measures

The primary and secondary outcomes are provided in section 4.2. Outcomes may be presented as mmol or the equivalent %.

9.4 Sample Size

Internal pilot

Thirty subjects will be recruited. 30 patients provides 80% power to detect a drop in the consent rate to 25% from the assumed 50%, at the 5% significance level.

Full Trial

A sample size of 143 in each group will have 80% power to detect a difference in means of 0.50 assuming that the common standard deviation is 1.50 using a two group t-test with a 0.05 two-sided significance level. Allowing for 10% loss to follow up means we would need a total of 316 participants (158 per group). The estimate used for the standard deviation in the sample size calculation was taken from an audit at Alder Hey Children's NHS Foundation Trust based on children matching the inclusion criteria for this proposed study. A difference in $HbA1_c$ of 0.5% is widely recognized as the threshold used by the Food and Drug Administration (FDA) and pharmaceutical industry to determine effectiveness of any new oral hypoglycaemic agents. Current national studies investigating therapeutic interventions in children with diabetes were powered using this effect size. An improvement of 0.61% was detected in adults in the meta-analysis of studies included in the 2004 HTA report suggesting that in addition to this estimate being the minimum clinically important it is also a realistic difference to detect.

9.5 Interim Monitoring and Analyses

Interim analyses of the accumulating data will be performed at regular intervals (at least annually) for review by the IDSMC. The IDSMC members will comply with a trial-specific IDSMC charter according to International Conference on Harmonisation Good Clinical Practice (ICH GCP) guidelines. These analyses will be performed at the MC-CTU by the trial statistician in accordance with the Standard Operating Procedures of the CTRC. The trial statistician at the MC-CTU will prepare the report for the IDSMC, the contents of which will be agreed by the IDSMC.

The data collected during the pilot phase will be used to check the estimate of the common standard deviation used in the sample size calculation of the full trial. The estimate obtained may be used to increase but not decrease the trial sample size. No formal comparisons of the trial primary outcome are planned at any interim analyses. If requested by the IDSMC the comparison will be made and the Peto-Haybittle stopping rule used to inform discussions.

The IDSMC will be asked to give advice on whether the accumulated data from the trial, together with results from other relevant trials, justifies continuing recruitment of further patients or further follow-up.

The full trial will open if the criteria listed in section 4.1 are met. A decision to discontinue recruitment, in all participants or in selected subgroups will be made only if the result is likely to convince a broad range of clinicians including participants in the trial and the general clinical community. If a decision is made to continue, the IDSMC will advise on the frequency of future reviews of the data on the basis of accrual and event rates. The IDSMC will make recommendations to the TSC (TSC, see section 16) as to the continuation of the trial.

9.6 Analysis Plan

The trial will be analysed and reported using the 'Consolidated Standard of Reporting Trials' (CONSORT) and the International Conference on Harmonisation E9 guidelines. A full and detailed statistical analysis plan will be developed prior any comparative analysis of the trial data. The main features of the statistical analysis plan are included here.

A p-value of 0.05 or less will be used to declare statistical significance for all analyses. Rather than adjust for multiplicity relevant results from other studies already reported in the literature will be taken into account in the interpretation of results.

Primary analysis will be the intention to treat principle, a secondary analysis will be conducted using the per protocol approach. The purpose of the per protocol approach is to consider the robustness of the conclusions reached from the analysis using the intention to treat principle to protocol deviations.

The primary outcome HbA1c will be compared between the trial groups using a two group t-test. Difference in means with 95% confidence intervals will be presented. Analysis of covariance will be used to adjust for baseline values (excluding HbA1c measured at baseline) and important prognostic factors.
Missing data will be monitored and strategies developed to minimise its occurrence, however as much data as possible will be collected about the reasons for missing data and this will be used to inform the handling of missing data.

9.7 Economic analysis

The primary economic analysis will be based on an assessment of the incremental costs per QALY gained. Additionally, a cost-consequences analysis will be performed based on the primary and secondary outcome measures. Costs and benefits will be discounted at an annual rate of 3.5% (36).

ICERs will be estimated, with uncertainty in parameter estimates being addressed through the application of bootstrapping and the estimation of cost-effectiveness acceptability curves (38). A regression analysis of cost and QALYs, with age, baseline $HbA1_c$, utility (39) and other covariates as deemed appropriate, will be conducted to conserve any correlation between costs and benefits, and minimise bias in the cost utility estimate. Estimates of ICERs will be compared with the £20,000 to £30,000 per QALY threshold of cost-effectiveness set by NICE (1), and a range of univariate and probabilistic sensitivity analyses will be conducted to assess the robustness of the analysis. Additionally, trial results will be extrapolated to estimate lifetime costs and benefits to avoid time horizon selection bias, and to capture long-term micro- and macrovascular complications. We will approach IMS Health for permission to use the CORE diabetes model (40) and parameterise with estimates derived from the trial.

10 PHARMACOVIGILANCE

10.1 Terms and Definitions

10.1.1 Medicines for Human Use (Clinical Trials) Regulations 2004 (SI 2004/1031) Definitions

As the insulin analogues are classed as investigational medicinal products (IMPs) in this trial, pharmacovigilance for SCIPI is subject to the following Medicines for Human Use (Clinical Trials) Regulations 2004 (SI 2004/1031) definitions:

Adverse Event (AE)
Any untoward medical occurrence in a subject to whom a medicinal product has been administered, including occurrences which are not necessarily caused by or related to that product.

Adverse Reaction (AR)
Any untoward and unintended response in a subject to an investigational medicinal product which is related to any dose administered to that subject.

Unexpected Adverse Reaction (UAR)
An adverse reaction the nature and severity of which is not consistent with the information about the medicinal product in question set out in:

- In the case of a product with a marketing authorization, in the summary of product characteristics for that product
- In the case of any other investigational medicinal product, in the investigator's brochure relating to the trial in question.

Serious Adverse Event (SAE), Serious Adverse Reaction (SAR) or Suspected Unexpected Serious Adverse Reaction (SUSAR)
Any adverse event, adverse reaction or unexpected adverse reaction, respectively, that:

- results in death
- is life-threatening* (subject at immediate risk of death)
- requires in-patient hospitalisation or prolongation of existing hospitalisation**
- results in persistent or significant disability or incapacity, or
- consists of a congenital anomaly or birth defect
- Other important medical events

*'life-threatening' in the definition of 'serious' refers to an event in which the patient was at risk of death at the time of the event; it does not refer to an event which hypothetically might have caused death if it were more severe.
**Hospitalisation is defined as an inpatient admission, regardless of length of stay, even if the hospitalisation is a precautionary measure for continued observation. Hospitalisations for a pre-existing condition, including elective procedures that have not worsened, do not constitute an SAE.
***Other important medical events that may not result in death, be life-threatening, or require hospitalisation may be considered a serious adverse event/experience when, based upon appropriate medical judgment, they may jeopardise the subject and may require medical or surgical intervention to prevent one of the outcomes listed in this definition.

10.1.2 National Research Ethics Service (NRES) Definitions

As SCIPI involves medical devices, safety reporting for the trial is subject to the following NRES safety reporting definitions for related and unexpected SAEs:

- **'related'** – that is, it resulted from administration of the medical device or any of the research procedures;
- **'unexpected'** – that is, the type of event is not listed in the protocol as an expected occurrence.

NRES require that a SAE occurring to a research participant, where in the opinion of the Chief Investigator the event is related and unexpected (RUSAE), is be reported to the main Research Ethics Committee (REC).

10.1.3 Medicines and Healthcare products Regulatory Agency (MHRA) Adverse Incident Centre (AIC) Definitions

As the trial involves the use of CE-marked medical devices employed for their intended purpose, adverse incidents are also reportable to the Medicines and Healthcare products Regulatory Agency (MHRA) Adverse Incident Centre (AIC) under the User Devices Vigilance requirement.

The MHRA AIC define an **Adverse Incident (AI)** as:

- An event that causes, or has the potential to cause, **unexpected or unwanted effects** involving the safety of device users (including participants) or other persons.

Causes of AIs involving devices may include:

- Design or manufacturing problems;
- Inadequate servicing and maintenance;
- Inappropriate local modifications;
- Unsuitable storage and use conditions;
- Selection of the incorrect device for the purpose;
- Inappropriate management procedures;
- Poor user instructions or training (which may result in incorrect user practice).

Conditions of use e.g. environmental conditions or location may also give rise to adverse incidents.

Any adverse incident involving a device or its instructions for use should be reported to the MHRA AIC, especially if the incident has led to or, were it to occur again, could lead to all occurrences listed under SAEs in section 10.1.1 above, as well as:

- Unreliable test results and associated risk of mis-diagnosis or inappropriate treatment;
- Ongoing faults that successive service/maintenance visits have failed to rectify.

The MHRA AIC also request that minor safety or quality problems with the device should also be reported as these can help demonstrate trends or highlight inadequate manufacturing or supply systems. Reports of adverse incidents that appear to be caused by human error should also be reported because:

- The error may be partly (or wholly) due to deficiencies in the design of the device or instructions for use;
- They may prompt promulgation of advice or device design improvements that will help prevent repetition of mistakes.

By these definitions AIs are the same as related and unexpected AEs and related and unexpected SAEs.

10.2 Severity / Grading of Adverse Events

The assignment of the severity/grading should be made by the investigator responsible for the care of the participant using the definitions below.
Regardless of the classification of an AE as serious or not, its severity must be assessed according to medical criteria alone using the following categories:

Mild: does not interfere with routine activities
Moderate: interferes with routine activities
Severe: impossible to perform routine activities

A distinction is drawn between serious and severe AEs. Severity is a measure of intensity (see above) whereas seriousness is defined using the criteria in section10.1.1, hence, a severe AE need not necessarily be a Serious AE.

10.3 Relationship to disease under study

Participants for this study will be monitored for adverse events and serious adverse events related to the disease under study. The assignment of causality should be made by the investigator responsible for the care of the participant. These events should be recorded on the related adverse events form and related serious event forms.

10.4 Relationship to Trial Treatment

Participants for this study will be monitored for adverse events and serious adverse events related to the trial treatment. The assignment of the causality should be made by the investigator responsible for the care of the participant using the definitions in table 2.
If any doubt about the causality exists the local investigator should inform the MC-CTU who will notify the Chief Investigator. In the case of discrepant views on causality between the investigators, the MHRA, NRES and MHRA AIC will be informed of both points of view.

Table 2: Definitions of Causality

Relationship	Description
**Unrelated**	There is no evidence of any causal relationship.<br>There is an alternative cause for the AE.
**Unlikely**	There is little evidence to suggest there is a causal relationship (e.g. the event did not occur within a reasonable time after administration of the trial intervention). There is another reasonable explanation for the event (e.g. the participant's clinical condition, other concomitant treatment).
**Possibly**	There is some evidence to suggest a causal relationship (e.g. because the event occurs within a reasonable time after administration of the trial intervention). However, the influence of other factors may have contributed to the event (e.g. the participant's clinical condition, other concomitant treatments).
**Probably**	There is evidence to suggest a causal relationship and the influence of other factors is unlikely.
**Almost certainly**	There is clear evidence to suggest a causal relationship and other possible contributing factors can be ruled out.

*Possibly, probably, or almost certainly related will be referred to throughout the protocol as 'related'.

10.5 Expectedness

Insulin analogue:
An AE whose causal relationship to the insulin is assessed by the investigator as “possible”, “probable”, or “definite” is an Adverse Drug Reaction. Refer to the relevant SPC for a list of expected adverse reactions. All events judged by the investigator to be possibly, probably, or almost certainly related to the insulin analogues, graded as serious (see section 10.1.1) and **unexpected** should be reported as a SUSAR.

Device:
An AE whose causal relationship to the device is assessed by the investigator as “possible”, “probable”, or “definite” is a Related Adverse Event. Refer to the relevant manufacturer’s instructions for use for list of expected related adverse events. All events judged by the investigator to be possibly, probably, or almost certainly related to the devices, graded as serious (see section 10.1.1) and **unexpected** should be reported as a Related Unexpected SAE.

10.6 Reporting Procedures

Non serious ARs/AEs
ARs/AEs will only be reported for participants where consent has been obtained and the causal relationship has been assessed and judged by the investigator to be related to the trial treatment (see section 10.4) and / or disease under study (type 1 diabetes), which occurs from the start of randomised treatment until final follow-up visit at 12 months post-diagnosis. The events should be recorded on a Related Adverse Event Form, which should be transmitted to the MC-CTU within seven days of the clinical research team becoming aware of the event.

Include the following only if they are related to the trial intervention.

a. An exacerbation of a pre-existing illness
b. An increase in frequency or intensity of a pre-existing episodic event/condition
c. A condition (even though it may have been present prior to the start of the trial) detected after administration of the trial treatment.
d. Continuous persistent disease or symptoms present at baseline that worsens following the administration of the trial treatment
e. Laboratory abnormalities that require clinical intervention or further investigation (unless they are associated with an already reported clinical event).
f. Abnormalities in physiological testing or physical examination that require further investigation or clinical intervention
g. Injury or accidents

Do Not Include

a. Any AEs whose causal relationship is assessed and judged by the investigator to be unrelated or unlikely to be related to the trial treatment and / or disease under study.
b. Medical or surgical procedures- the condition which leads to the procedure is the adverse event
c. Pre-existing disease or conditions present before treatment that do not worsen
d. Situations where an untoward medical occurrence has occurred e.g. cosmetic elective surgery
e. Overdose of medication without signs or symptoms

Serious ARs/AEs/SUSARs/RUSAEs
SARs, SAEs, SUSARs and RUSAEs will only be reported for participants where consent has been obtained and the causal relationship has been assessed and judged by the investigator to be related to the trial treatment (see section 10.4) and / or disease under study (see section 10.3), which occurs from the start of randomised treatment until final follow-up visit at 12 months post-diagnosis. They should be reported to the MC-CTU using the related SAE form **within 24 hours** of the local site becoming aware of the event.

The related SAE form asks for the nature of event, date of onset, severity, corrective therapies given, outcome and causality. The responsible investigator should sign the causality of the event. Additional information should be sent within 5 days if the reaction/event has not resolved at the time of reporting.

Refer to section 10.9 and 10.10 for further details on the reporting procedures and the responsibilities of the investigators and the MC-CTU respectively. Any questions concerning adverse event reporting should be directed to the MC-CTU in the first instance. A flowchart is given below to aid in determining reporting requirements.

All hospital admissions will be reported as part of the routine baseline and follow-up CRFs and using electronic databases. Only report hospital admissions using the related SAE CRF if they occur in the specified time frame and are judged to be related to the trial treatment.

All deaths that occur between the time of consent and the 12 month follow-up should be reported to the MC-CTU using the death CRF **within 7 days** of the clinical research team becoming aware of the event. If a patient's death has been assessed and judged by the investigator to be related to the trial treatment (see section 10.4), a related SAE CRF should also be completed.

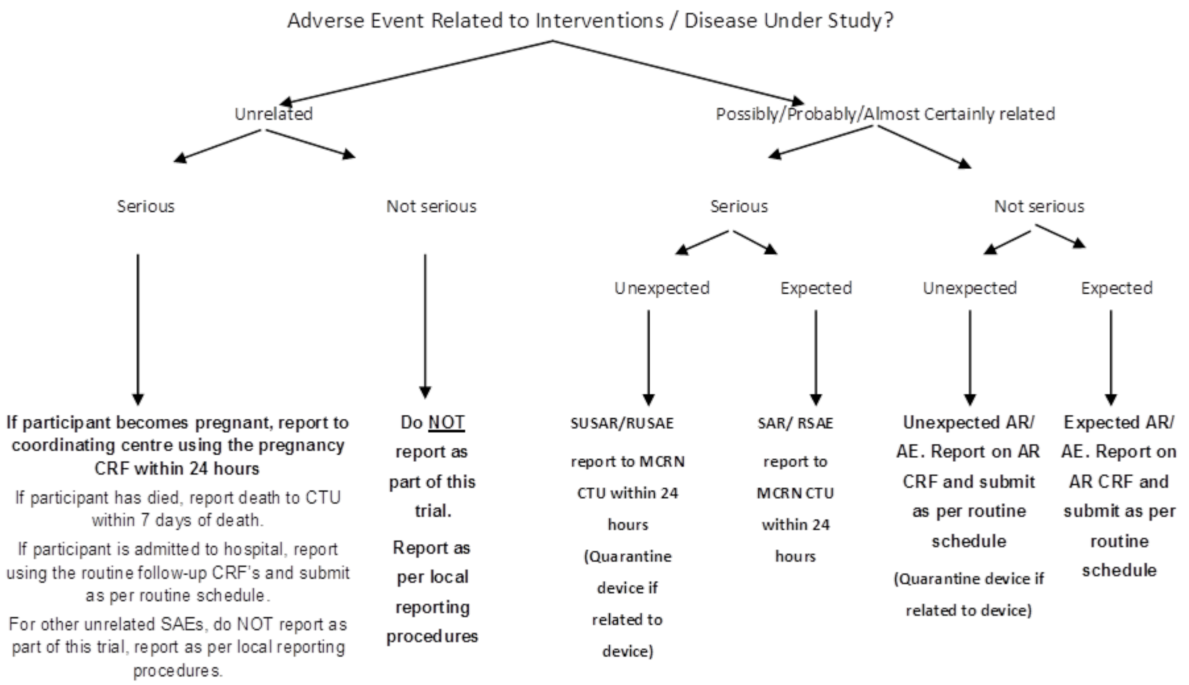

10.6.1 Reporting of Pregnancy

Study participants will not routinely be tested for pregnancy as part of the trial screening process. Any pregnancy which does occur during the course of the study should be reported to the MC-CTU immediately. It is at the investigator's discretion to decide whether the individual should be instructed to stop taking study drugs. All pregnancies that occur during trial treatment, or within seven days of finishing treatment, need to be followed up until completion and reported separately. The clinician should review insulin delivery.

10.7 Follow-up After Adverse Events

All related SARs/SAEs/ARs/AEs should be followed until satisfactory resolution (clinical recovery is complete and/or laboratory results have returned to normal) or until the investigator responsible for the care of the participant deems the event to be chronic or the patient to be stable. Follow-up may continue after removal of the study treatment if necessary.

Follow-up information for a related AE should be noted on the related AE form under the heading marked 'outcome'. Follow-up information for a related SAE should be noted on another related SAE form by ticking the box marked 'follow-up' and faxing to the MC-CTU as information becomes available. Extra, annotated information and/or copies of test results may be provided separately.

When reporting related AEs/SAEs the investigator responsible for the care of the participant should apply the following criteria to provide information relating to event outcomes: resolved; resolved with sequelae (specifying with additional narrative); not resolved/ongoing; ongoing at final follow-up; fatal or unknown.

10.8 Quarantine, Labelling & Storage of Devices Involved in an Adverse Incident (i.e. Related Unexpected AE/SAE)

Medical devices that have been involved in an adverse incident (i.e. related and unexpected AE), whether serious or not, should be quarantined.

Until the MHRA has been given the opportunity to carry out an investigation, they should not be discarded, repaired or returned to the manufacturer. All material evidence, i.e. devices/parts removed, replaced or withdrawn from use following an incident, instructions for use, records of use, repair and maintenance records, packaging materials, or other means of batch identification must be:

- Clearly identified and labelled;
- Stored securely.

Evidence should not be interfered with in any way except for safety reasons or to prevent its loss. Where appropriate, a record should be made of all readings, settings and positions, together with any photographic evidence and eyewitness reports.

If it is thought that an urgent examination of the device (and/or related items) may be required, upon notification of the incident an MHRA device specialist will decide whether to inspect the item urgently on site (or at other appropriate facilities), or may request that the device is sent to the MHRA. If required, the MHRA will contact the manufacturer and, if accompanied by an appropriate person, they may be allowed to inspect the items. To facilitate an investigation, it may be possible to provide the manufacturer with a sample of unused stock from a large batch. However, until advised to the contrary by the MHRA, the manufacturer must not be allowed to exchange, interfere with, or remove any part of the

product implicated in the incident as this might prejudice MHRA investigations, or those of other official bodies.

10.9 Responsibilities – Investigator

The Investigator is responsible for reporting all related SAEs and related AEs/ARs that are observed or reported during the study. All related SAEs must be reported immediately by the investigator to the MC-CTU on an SAE form. All other related AEs/ARs should be reported on the related AE form as part of routine follow-up.

Minimum information required for reporting:

- Study identifier
- Study centre
- Participant number
- A description of the event
- Date of onset
- Current status
- Whether study treatment was discontinued
- The reason why the event is classified as serious
- Investigator assessment of the association between the event and study treatment

i. The related SAE form should be completed by a designated investigator, a clinician named on the 'signature list and delegation of responsibilities log' as responsible for reporting SAEs and making trial related medical decisions. The investigator should assess the SAE for the likelihood that it is a response to the investigational medicinal product and/or the medical device. In the absence of the designated investigator the form should be completed and signed by an alternative member of the research site trial team and submitted to the MC-CTU. As soon as possible thereafter the responsible investigator should check the SAE form, make amendments as appropriate, sign and re-send to the MC-CTU. The initial report shall be followed by detailed reports as appropriate.

ii. When submitting an SAE to the MC-CTU research sites should also telephone the appropriate trial manager/data manager to inform that an SAE report has been submitted. (The MC-CTU trial team should ensure that the number to be used to report SAEs in this way is manned during office hours, and is notified to research site personnel during the site initiation process.)

Send the SAE form by fax (within 24 hours or next working day) to the MC-CTU

iii. The responsible investigator must **notify** their R&D department of the event (as per standard local governance procedures).

iv. In the case of a related SAE the participant must be followed-up until clinical recovery is complete and laboratory results have returned to normal, or until the event has stabilised. Follow-up may continue after completion of protocol treatment if necessary.

v. Follow-up information is noted on another SAE form by ticking the box marked 'follow-up' and faxing to the MC-CTU as information becomes available. Extra, annotated information and/or copies of test results may be provided separately.

vi. The participant **must** be identified by trial number and initials only. The participant's name **should not** be used on any correspondence.

vii. For medical devices that have been involved in an adverse incident (related unexpected AE), whether classed as serious or not, ensure that they have been quarantined as described in section 10.8

viii. The responsible investigator must notify their R&D department and medical device liaison officer of the event as per standard local governance procedures.

ix. As soon as you become aware of a related, unexpected adverse event (whether serious or not) involving a medical device, complete the Medical Device Adverse Incident (AI) CRF and return to the MC-CTU within 24 hours.

Participant safety incidents that take place in the course of research should be reported to the National Patient Safety Agency (NPSA) by each participating NHS Trust in accordance with local reporting procedures.

10.10 Responsibilities – MC-CTU

The MC-CTU is undertaking duties delegated by the trial sponsor Alder Hey Children's NHS Foundation Trust, and is responsible for the reporting of AEs/ARs to the MHRA, the main REC and MHRA AIC as follows:

- SUSARs (Insulin analogue related and unexpected) which are fatal or life-threatening must be reported to the MHRA and Main REC not later than 7 days after the MC-CTU is first aware of the reaction. Any additional relevant information must be reported within a further 8 days.
- SUSARs (Insulin analogue related and unexpected) that are not fatal or life-threatening must be reported to the MHRA and Main REC within 15 days of the MC-CTU first becoming aware of the reaction.
- A list of all suspected SARs (Insulin analogue related, expected and unexpected) must be reported annually to the MHRA and Main REC.
- RUSAEs (device related and unexpected) should be reported to the Main REC within 15 days of the MC-CTU first becoming aware of the event.
- A list of all device related SAEs (expected and unexpected) should be reported annually to the Main REC.
- All device-related unexpected SAEs and AEs (Adverse Incidents) will be reported to the MHRA AIC as part of user device vigilance reporting.
- Copies of the reports will be sent to the Principal Investigator at all institutions participating in the trial

It is recommended that the following safety issues should also be reported in an expedited fashion

- An increase in the rate of occurrence or a qualitative change of an expected serious adverse reaction, which is judged to be clinically important;
- Post-study SUSARs that occur after the participant has completed a clinical trial and are notified by the investigator to the sponsor;
- New events related to the conduct of the trial or the development of the insulin analogues and likely to affect the safety of the subjects, such as:
    a. A SAE which could be associated with the trial procedures and which could modify the conduct of the trial;
    b. A significant hazard to the subject population, such as lack of efficacy of an insulin analogue used for the treatment of a life-threatening disease;

c. A major safety finding from a newly completed animal study (such as carcinogenicity).
d. Any anticipated end or temporary halt of a trial for safety reasons and conducted with the same insulin analogue in another country by the same sponsor;

- Recommendations of the Independent Data and Safety Monitoring Committee, if any, where relevant for the safety of the participants.

Staff at the MC-CTU will liaise with the Chief Investigator (or designated other specified in the protocol) who will evaluate all SAEs received for seriousness, expectedness and causality. The causality assessment given by the Local Investigator at the hospital cannot be overruled and in the case of disagreement, both opinions will be provided with the report.

10.11 Safety reports

Safety reports will be generated during the course of the trial which allows for monitoring of AE and AR reporting rates across sites. The MC-CTU will send annual safety reports containing a list of all SARs/SAEs to regulatory authorities and MREC as detailed in Section 10.9. Any concerns raised by the IDSMC or inconsistencies noted at a given site may prompt additional training at sites, with the potential for the MC-CTU to carry out site visits if there is suspicion of unreported AEs in patient case notes. Additional training will also be provided if unacceptable delay in safety reporting timelines.

11 ETHICAL CONSIDERATIONS

11.1 Ethical Considerations

The study will abide by the principles of the World Medical Association Declaration of Helsinki (1964) and the Tokyo (1975), Venice (1983), Hong Kong (1989) and South Africa (1996).

The specific ethical issues are:

Informed consent in a paediatric population: The parent or legal representative of the child will have an interview with the investigator, or a delegated member of the investigating team, during which opportunity will be given to understand the objectives, risks and inconveniences of the trial and the conditions under which it is to be conducted. They will be provided with written information and contact details of the diabetes study nurse, from whom further information about the trial may be obtained, and will be made aware of their right to withdraw the child from the trial at any time without the child or family being subject to any detriment in the child's treatment. Children will receive information, according to their capacity of understanding, about the trial and its risks and benefits and their assent will be obtained, where appropriate.

Use of insulin analogues in younger population:
Insulin aspart is an insulin analogue licensed for the treatment of TIDM in adults, adolescents and children aged 2 to 17 years and should only be used in children under 2 years under careful supervision; however the rapid onset and offset of action of this insulin make it particularly attractive in the management of young children. For this reason it is widely used in young children with TIDM. The safety and efficacy of insulin glargine (Lantus) have been established in adolescents and children aged 2 years and older. Insulin glargine has not been studied in children below the age of 2 years; however, the use of insulin glargine in this age group has been associated with a reduction in hypoglycaemia and improved metabolic control (28). For these reasons it is widely used in intensive treatment regimens in British paediatric practice.

11.2 Ethical Approval

The trial protocol, including the Parent/Patient Information Sheets and Consent/Assent forms and all other relevant study documentation has received the favourable opinion of the North West 3 REC - Liverpool East Multi-centre Research Ethics Committee (MREC). All participating centres must be granted NHS permission by their Local Research & Development (R&D) department prior to commencing recruitment. A copy of local R & D approval and the Parent/Patient Information and Consent/Assent form on local headed paper should be forwarded to MC-CTU before the site is initiated and patients recruited.

Proxy consent from the parent or legally acceptable representative should be obtained prior to each patient participating in the trial, after a full explanation has been given of the treatment options, including the conventional and generally accepted methods of treatment. Age and stage-of development specific Patient Information and Consent Forms (PISC) should also be implemented and patient assent obtained where appropriate. The right of the parent/ legal representative to refuse consent for the minor to participate in the trial without

giving reasons must be respected. After the patient has entered the trial, the clinician must remain free to give alternative treatment to that specified in the protocol, at any stage, if he/she feels it to be in the best interest of the patient. However, the reason for doing so should be recorded and the participant will remain within the trial for the purpose of follow-up and data analysis. Similarly, the parent/legal representative of the participant remains free to withdraw the participant at any time from the protocol treatment and trial follow-up without giving reasons and without prejudicing the further treatment of the minor.

11.3 Informed Consent Process

Informed consent is a process initiated prior to an individual agreeing to participate in a trial and continues throughout the individual's participation. Informed consent is required for all participants participating in MC-CTU coordinated trials. In obtaining and documenting informed consent, the investigator should comply with applicable regulatory requirements and should adhere to GCP and to the ethical principles that have their origin in the Declaration of Helsinki.

Discussion of objectives, risks and inconveniences of the trial and the conditions under which it is to be conducted are to be provided to patients/ parents by staff with experience in obtaining informed consent. Age-and-stage-of-development appropriate Patient Information and Consent forms, describing in detail the trial interventions, trial procedures and risks will be approved by an independent ethical committee (IEC) and the patient and their parent/legal representative will be asked to read and review the document. Upon reviewing the document, the investigator will explain the research study to the patient and their parent/legal representative. This information will emphasise that participation in the trial is voluntary and that the participant may withdraw from the trial at any time and for any reason. All participants will be given opportunity to ask any questions that may arise, should have the opportunity to discuss the study with their surrogates and as long as they need to consider the information prior to agreeing to participate. A contact point where further information about the trial may be obtained will be provided.

Both the person taking consent and the parent/legal representative must personally sign and date the consent form. A copy of the informed consent document will be given to the patient and their legally acceptable representative for their records. The original copy will be filed in the participant's notes and a further copy of the signed consent form will be given to the participant. One final copy of the consent form should be sent to the MC- CTU.

Assent in minors

If capable, and under appropriate circumstances, minors should be approached to provide assent by a member of the research team with experience with minors. Age-and-state-of-development IEC-approved Patient information Sheet and Assent forms, describing (in simplified terms) the details of the trial intervention, trial procedures and risks should be used. The minor should personally write their name and date the assent form, which is then signed by the parent/legal representative and the researcher.

Assent forms do not substitute for the consent form signed by the patient's legally acceptable representative. Assent should be taken where appropriate and documented in the patient notes, however the absence of assent does not exclude the patient provided consent has been obtained from the parent/legal representative.

11.4 Study Discontinuation

In the event that the study is discontinued, participants will be treated according to usual standard clinical care. The process for participants who withdraw early from trial treatment or from the trial completely is described in section 5.3

12 REGULATORY APPROVAL

This trial is comparing alternative methods of insulin delivery via CE-marked medical devices employed for their intended purpose, therefore, this trial is not considered to be a clinical investigation under the Medical Devices Regulations 2002.

The trial does, however, fall within the remit of the EU Directive 2001/20/EC, transposed into UK law as the UK Statutory Instrument 2004 No 1031: Medicines for Human Use (Clinical Trials) Regulations 2004 as amended. Therefore, this trial has been registered with the MHRA and has been granted a Clinical Trial Authorisation (CTA). The CTA reference is 2010-023792-25.

13 TRIAL MONITORING

Trial monitoring is carried out to ensure that the rights and well-being of human participants are protected during the course of a clinical trial and that the data are credible and accurate. A risk assessment is performed for each trial coordinated by the MC-CTU to determine the level and type of monitoring required for specific hazards. The nature and extent of monitoring will be specific to the individual trial. Monitoring can take the form of on-site visits or central monitoring.

Monitoring of the SCIPI trial will be informed by the SCIPI risk assessment and will be conducted as per a detailed monitoring plan, which will describe who will conduct the monitoring, at what frequency monitoring will be done, and what level of detail monitoring will be conducted.

Trial Oversight Committees related to the monitoring of the trial are detailed in section 16.

13.1 Source Documents

Source data*: All information in original records and certified copies of original records of clinical findings, observations, or other activities in a clinical trial necessary for the reconstruction and evaluation of the trial. Source data are contained in source documents (original records or certified copies) (ICH E6, 1.51).*

Source documents*: Original documents, data, and records (e.g., hospital records, clinical and office charts, laboratory notes, memoranda, subjects diaries or evaluation checklists, pharmacy dispensing records, recorded data from automated instruments, copies or transcriptions certified after verification as being accurate copies, microfiches, photographic negatives, microfilm or magnetic media, x-rays, subject files, and records kept at the pharmacy, at the laboratories and at medico-technical departments involved in the clinical trial)(ICH E6, 1.52).*

In order to resolve possible discrepancies between information appearing in the CRF and any other participant related documents, it is important to know what constitutes the source document and therefore the source data for all information in the CRF. The data recorded in the CRF should be consistent and verifiable with source data in source documents other than the CRF (e.g. medical record, laboratory reports and nurses' notes).

Each participating site should maintain appropriate medical and research records for this trial, in compliance with International Conference on Harmonisation – E6- Good Clinical Practice guidelines Section 4.9 and regulatory and institutional requirements for the protection of confidentiality of participants.

Source data will be identified and documented in the SCIPI Trial Monitoring Plan.

13.2 Data Capture Methods

Case Report Forms
The study case report form (CRF) is the primary data collection instrument for the study. All data requested on the CRF must be recorded. All missing data must be explained. If a space on the CRF is left blank because the procedure was not done or the question was not asked, write "N/D". If the item is not applicable to the individual case, write "N/A". Or if the data item is un-known, write "NK". If a data item has not been recorded on source data then write 'NR'. All entries should be printed legibly in black ink. If any entry error has been made, to correct such an error, draw a single straight line through the incorrect entry and enter the correct data above it. All such changes must be initialled and dated. DO NOT ERASE OR WHITE OUT ERRORS. For clarification of illegible or uncertain entries, print the clarification above the item, then initial and date it.

Patient completed data
Participants/Parents complete the following questionnaires at specified time points throughout the trial:

- Quality of Life (PedsQL)
- Health Status (HUI2/3)

The participant initials and randomisation number should be clearly labelled on all documents. The "centre use only" section on the front cover of the questionnaire and diaries should also be completed. This records the dates given/completed and the type of visit (if applicable). Diaries and questionnaires should be returned to the CTU alongside the relevant CRF pages where possible (e.g. 3 month questionnaire booklet forwarded with the 3 month follow-up CRF pages). For further details on the administration of the questionnaires refer to section 8.4.

For details on treatment diary completion and use refer to section 8.2.2.

Electronic Data Download from CSII pumps and Glucometers
Guidance detailing the process of download and transfer of electronic data will be provided to centres in the investigator site files. Also refer to section 8.2.2 and 8.2.3.

Data from electronic routine administrative databases
Data from hospital Patient Administration Systems will be obtained as described in section 8.4.2. Collection of these data will follow a standard procedure. Any transfer of data (requests for data and the return of the full dataset) will be transferred securely (encrypted). Data will be stored at the MC-CTU as described in section 13.6. Consent to data linkage will be sought.

13.3 Data Monitoring at MC-CTU

Data stored at MC-CTU will be checked for missing or unusual values (range checks) and checked for consistency within participants over time. Any suspect data will be returned to the site in the form of data queries. Data query forms will be produced at the MCCTU from the trial database and sent either electronically or through the post to a named individual (as listed on the site delegation log). Sites will respond to the queries providing an explanation/resolution to the discrepancies and return the data query forms to MC-CTU. The forms will then be filed along with the appropriate CRFs and the appropriate corrections made on the database. There are a number of monitoring features in place at the MC-CTU

to ensure reliability and validity of the trial data, which will be detailed in the trial monitoring plan.

13.4 Clinical Site Monitoring and Direct Access to Data

In order to perform their role effectively, the trial coordinator (or monitor) and persons involved in Quality Assurance, Audit and Inspection may need direct access to primary data, e.g. participant records, laboratory reports, appointment books, etc. Since this affects the participant's confidentiality, this fact is included on the Parent Information Sheet and Informed Consent Form.

13.5 Central Monitoring

Central monitoring will be conducted as described in the SCIPI trial monitoring plan.

13.6 Confidentiality

All individual participant information obtained as a result of this study is considered confidential, and will be handled, stored and destroyed in accordance with the Data Protection Act 1998. No names will be used in any publications or reports.

Case report forms will be labelled with the participant's initials and unique trial screening and/or randomisation number. Medical information may be given to the participant's medical team and all appropriate medical personnel responsible for the participant's welfare.

The MC-CTU will be undertaking activities requiring the transfer of identifiable data: Verification that appropriate informed consent is obtained will be enabled by the provision of copies of participant's signed informed consent/assent forms being supplied to the MC- MC-CTU by recruiting centres. This requires that name, participant initials, NHS number, postcode and date of birth data will be transferred on the consent form to the MC-CTU, which is disclosed in the information sheet and consent form. The assent forms will also contain name data.

Trial data collected on paper will be sent to the MC-CTU and filed in locked filing cabinets. Paper copies of the consent/assent form will be kept separately to the clinical data. Name data will not be stored electronically. The following personal data will be stored electronically on the MC-CTU servers: NHS Number, postcode, date of birth, participant initials and participant trial randomisation number. The NHS number, postcode, date of birth and trial randomisation number will be stored in a separate encrypted database with controlled access. The participant's initials, date of birth and trial number will be stored in the unencrypted MACRO database. Transfer of NHS Number, date of birth and postcode to obtain resource use data from electronic routine administrative databases will be encrypted. The MC-CTU servers are located in an access controlled server room and are connected to the main university network, located behind a firewall. Physical access to these servers is limited to members of the Universities computing services department; MC-CTU Information Systems staff have access to the server consoles. Trial data will be stored in a SQL server database with access limited to MC-CTU staff with permission to access the trial data held on the MACRO (Infermed) system and MC-CTU Information Systems staff with database access privileges. MC-CTU staff accounts on the MACRO system have different credentials to that required by the University computing systems (which must be accessed prior to logging into MACRO). Access to MACRO is limited to staff using the Universities network. The SQL Server database can only be accessed by computers with a University IP address.

Members of the research team outside the MC-CTU will have access to data generated by the trial, which is relevant to their role, but this will be anonymised.

The MC-CTU will preserve the confidentiality of participants taking part in the study and The University of Liverpool is registered as a Data Controller with the Information Commissioners Office.

13.7 Quality Assurance and Control

QA includes all the planned and systematic actions established to ensure the trial is performed and data generated, documented/recorded and reported in compliance with applicable regulatory requirements. QC includes the operational techniques and activities done within the QA system to verify that the requirements for quality of the trial-related activities are fulfilled. In accordance with the monitoring plan, site visits will be conducted and source data verification performed if indicated to be required as a result of central monitoring processes. To this end:

- The Principal Investigator and RN from each centre will attend the trial launch meeting, coordinated by MC-CTU in conjunction with the Chief investigator, Dr Joanne Blair, which will incorporate elements of trial- specific training necessary to fulfil the requirements of the protocol;

- The Trial Coordinator is to verify appropriate approvals are in place prior to initiation of a site and the relevant personnel have attended trial specific training;

- The Trial Coordinator is to check safety reporting rates between centres;

- The Trial Coordinator is to monitor screening, recruitment and drop-out rates between centres;

- The Trial Coordinator is to conduct data entry consistency checks and follow-up data queries;

- Independent oversight of the trial will be provided by the Data and Safety Monitoring Committee and independent members of the Trial Steering Committee.

13.8 Records Retention

The investigator at each investigational centre must make arrangements to store the essential trial documents, (as defined in Essential Documents for the Conduct of a Clinical Trial (ICH E6, Guideline for Good Clinical Practice) including the Investigator Site File, until the MC-CTU informs the investigator that the documents are no longer to be retained, or for a maximum period of 15 years (whichever is soonest).

In addition, the investigator is responsible for archiving of all relevant source documents so that the trial data can be compared against source data after completion of the trial (e.g. in case of inspection from authorities).

The investigator is required to ensure the continued storage of the documents, even if the investigator, for example, leaves the clinic/practice or retires before the end of required storage period. Delegation must be documented in writing.

The MCCTU undertakes to store originally completed CRFs and separate copies of the above documents for the same period, except for source documents pertaining to the individual investigational centre, which are kept by the investigator only. The MC-CTU will

archive the documents in compliance with ICH GCP utilising the Records Management Service of the University of Liverpool. All electronic CRFs and trial data will be archived onto an appropriate media for long term accessible storage. Hard copies of data will be boxed and transferred to specially renovated, secure, premises where unique reference numbers are applied to enable confidentiality, tracking and retrieval.

14 INDEMNITY

SCIPI is sponsored by the Alder Hey Children‟s NHS Foundation Trust, and co-ordinated by the MC-CTU in the University of Liverpool. As this is an investigator-initiated study, The Association of the British Pharmaceutical Industry (ABPI) guidelines for patient compensation by the pharmaceutical industry do not apply.

Alder Hey Children‟s NHS Foundation Trust shall provide an indemnity in respect of Clinical Negligence to the extent that such an indemnity is permitted by the NHS Litigation Authority‟ s Clinical Negligence Scheme for Trusts.

For the purposes of the study Clinical Negligence is defined as:-
“A breach of duty of care by members of the health care professions employed by NHS bodies or by others consequent on decisions or judgments made by members of those professions acting in their professional capacity in the course of their employment, and which are admitted as negligent by the employer or are determined as such through the legal process” (NHS Indemnity: Arrangements for Clinical Negligence Claims in the NHS October 1996).

15 FINANCIAL ARRANGEMENTS

The research cost of the study is funded by the Department of Health, National Institute for Health Research Health Technology Assessment programme (NIHR HTA). Contractual agreements will be in place between sponsor and collaborating sites that will incorporate financial arrangements.

Participants and their parents / guardians will not be paid to participate in the trial. The schedule of the study will be in line with routine standard care where possible.

15.1 Financial Support for Collaborating Sites

15.1.1 Staffing

The HTA has provided funding for six specialist RNs (2xFTE grade 7, 39 months; 2xFTE grade 6, 39 months; 2xFTE grade 6, 33 months), which will be employed at and allocated to the participating centres to support the identification, recruitment and management of participants for the SCIPI trial.

As the study is funded by the NIHR HTA, it will be automatically adopted onto the NIHR portfolio, which will allow trusts to apply to their comprehensive local research network for service support costs if required.

15.1.2 Intervention Supplies from Roche

The Department of Health NIHR HTA, have advised that the funding for the CSII pumps and pump consumables are not research costs but should be treated as excess treatment costs. However, Department of Health (DoH) England and Research and Development Office for the Health and Personal Social Services in Northern Ireland have agreed to provide subvention funding to cover the costs of the CSII pumps and 50 % of pumps consumables for the trial centres in England and Belfast respectively. Whilst Wales Office of Research and Development for Health and Social Care, Welsh Assembly Government in Cardiff have agreed to provide 100 % funding for the CSII pumps and pumps consumables at Cardiff centre. All remaining excess treatment costs will be met by the NHS through local commissioning arrangements.

Roche will supply the CSII pumps and consumables for use in the SCIPI trial. They have guaranteed the cost of the CSII pumps at a special concessional rate throughout the trial period for trial participants. The consumables include a 25% discount on the current list price and will be subject to 4% inflation in April each year. Roche has also agreed to supply free Expert glucometers for the participants on MDI treatment.

16 TRIAL COMMITTEES

16.1 Trial Management Group (TMG)

A Trial Management Group (TMG) will be formed comprising the Chief Investigator, other lead investigators (clinical and non-clinical) and members of the MC-CTU. The TMG will be responsible for the day-to-day running and management of the trial and will meet initially every month during trial set up and subsequently every 3 months once the trial is in recruitment.

16.2 Trial Steering Committee (TSC)

The Trial Steering Committee will consist of an independent chairperson, Dr Peter E Clayton, two independent experts in the field of Diabetes and Endocrinology, Dr Christine P Burren and Dr Ian Craigie, an expert in medical statistics Dr Gordon D Murray, a parent representative Mrs Christina McRoe and up to seven Principal Investigators. The role of the TSC is to provide overall supervision for the trial and provide advice through its independent Chairman. The ultimate decision for the continuation of the trial lies with the TSC.

16.3 Independent Data and Safety Monitoring Committee (IDSMC)

The independent Data and Safety Monitoring Committee (IDSMC) consists of an independent chairperson, Professor Stephen Greene plus 2 independent members: Professor John Wilding who is an expert in the field of Endocrinology and Dr Arne Ring, an expert in medical statistics.

The IDSMC will be responsible for reviewing and assessing recruitment, interim monitoring of safety and effectiveness, trial conduct and external data. The IDSMC will first convene prior to trial initiation and will then define frequency of subsequent meetings (at least annually).

Details of the interim analysis and monitoring are provided in section 9.5.
The IDSMC will provide a recommendation to the Trial Steering Committee concerning the continuation of the trial.

17 PUBLICATION

The results from different centres will be analysed together and published as soon as possible. Individual Clinicians must undertake not to submit any part of their individual data for publication without the prior consent of the Trial Management Group.

The Trial Management Group will form the basis of the Writing Committee and advice on the nature of publications. The Uniform Requirements for Manuscripts Submitted to Biomedical Journals (http://www.icmje.org/) will be respected. All publications shall include a list of participants, and if there are named authors, these should include the trial's Chief Investigator(s), Statistician(s) and Trial Manager(s) involved at least. If there are no named authors (i.e. group authorship) then a writing committee will be identified that would usually include these people, at least. The ISRCTN allocated to this trial should be attached to any publications resulting from this trial.
The members of the TSC and IDSMC should be listed with their affiliations in the acknowledgements/ Appendix of the main publication.

18 PROTOCOL AMENDMENTS

18.1 Version 2.0 (30/MAR/2011)

Amended Protocol Section

Section	Amendments
1. Protocol Summary	Inclusion Criteria (Refer Section 5) and secondary endpoint (Refer Section 2.3)
2.3 Objectives	In the secondary objective, prevalence of adverse events changed to incidence of severe hypoglycaemia and diabetic ketoacidosis.
4.2 Trial Design	Refer Section 2.3
5. Inclusion Criteria	Inclusion of patients and parents able to complete study material
6.6 Randomisation	Randomisation envelopes to be used as a back-up to the telephone randomisation system
7.3 Formulation, packing, labelling, storage and stability of trial treatment	Insulin pumps, Insulin pen injectors and glucometers suppliers will be as per local policy rather than specified manufacturers (still used within CE-marking)
8 Assessment and Procedures	• HbA1c samples will be collected, analysed and destroyed according to local clinical practice rather than analysis at a central laboratory<br>• Adverse events See section 2.3 (objective change) and section 10 (Pharmacovigilance)<br>• New version of Health Utilities Index Questionnaire; (HUInc. 2002). HUI2/3 completed by children 12 years of age and above is included.<br>• NHS hospital admission will be collected from electronic routine NHS health care records (patient administration systems). Participant's NHS number and post code will be collected to facilitate this.
10 Pharmacovigilance	Only related SAEs and related AEs will be reported for this trial. RUSAEs related to medical devices will be reported as per user vigilance reporting.
13.2 Data capture Methods	Refer to section 8: Assessment and Procedures
13.6 Confidentiality	Refer to section 8: Assessment and Procedures
16 Trial Committees	Membership of IDMSC and TSC

18.2 Version 3.0 (01/JULY/2011)

Amended Protocol Section

Section	Amendments
4.2 Trial Design<br>6.1 Screening	The time period to start the randomised treatment was changed from within 3-5 days to within 10 days. The study information will be provided and the consent should occur as close to the time of diagnosis as possible, ideally between the time of diagnosis (Day 0) and Day 5.<br><br>Timelines for providing information and approaching the patient for consent will be recorded on the screening log.
6.3 Randomisation	Web randomisation system to be used, with randomisation envelopes as a back-up.

8 Assessment and Procedures<br><br>Schematic Representation of Study Design in Protocol Summary	Trial assessment table was modified to clarify the parameters.<br><br>PedsQL (Quality of life) questionnaires booklets were removed from baseline and will only be administered at 6 and 12 month study visit. Flowchart also updated to reflect this.
Contact details<br>16 Trial Committees	Membership of IDSMC changed and contact details updated.

18.3 Version 4.0 (17/August/2012)

Amended Protocol Section

Section	Amendments
Contact details section	Removal of all study team contacts except Sponsor, CI and Medical Expert.
1. Protocol Summary	Revision to the inclusion criteria, change to patients and parents able to comply with the treatment regimen and study visits.
	Addition to exclusion criteria list:<br>g. Known thyroid condition in a non Euthyroid state and;<br>h. Known Coeliac disease unable to maintain a gluten free diet.<br>See also changes to section 5.2
	Revision to the exclusion criteria change to:<br>a. have a sibling with existing TIDM<br>See also changes section 5.2
3.1 Selection of centres	Centre inclusion criteria, addition of the requirement for centres to be already be offering MDI regimen.
4.0. Trial design	Additional guidance and change to recruitment window period to 14 days and further guidance on patients being_approached and consented as soon after diagnosis as possible.<br>See also section 6.1,7.1 and 8.1
4.1 Trial Design: Pilot study	Further demographics characteristics to be analyses specified; ethnicity.
4.2 Trial Design: Full Trial	Collection of long term outcome data for nine years instead of four.
7.1 Trial Treatments	Correction to the caution for use of Insulin Aspart as; analogue licensed for the treatment of TIDM in adults, adolescents and children aged 2 to 17 years but should only be used in children under 2 years under careful supervision;<br>Refer to section 11.1
7.3 Formulation, packing, labelling, storage and	Clarification on existing devices to be used and clarification of existing process for Insulin storage

stability of trial treatment	requirements.<br><br>Revised wording of statement of compliance for labelling waiver for clarity.
7.4 Preparation, Dosage and Administration of Study Treatment/s:	Additional information around existing consumable supplies process added.
7.5 Dose Modifications	Revision to guidance on contacts between diabetes clinical team and with families to be according to participant need.
8.0 Assessments and Procedures	Further clarification to existing process for returning CRFs within 7 days to the MCRN.
8.1 Schedule for Follow up	Correction to the participant follow up period to be one year from the date of randomisation not diagnosis.
	Changes to reflect follow up from randomisation:<br>- Assessment of Related Adverse Events<br>- Addition of : Assessment of AE's related to diabetes<br>- Addition of: 'and a sample to be sent for central analysis' to footnote 4.
8.2.1 Procedures for Assessing efficacy: HbA1c	HbA1c samples to be collected, analysed and destroyed at a central laboratory.
8.3.1 Procedures for assessing safety: adverse events (paragraph 1 and 2)	Clarification on the existing process to collect treatment related serious adverse events. Additional statement added to confirm that adverse events related to the disease under study will also be monitored.
8.6 Trial Closure	Definition of end of the trial changed to be the date on which data entry privileges are withdrawn from the trial database.
9.6 Analysis Plan	Clarification that analysis of covariance will be used to adjust for baseline values (excluding HbA1c measured at baseline) and important prognostic factors.
10.5 Reporting procedures (flow diagram)	Additional guidance:<br>• Assessment for AE disease under study or trial interventions<br>• Guidance on: If participant becomes pregnant, report to coordinating centre using the pregnancy CRF within 24 hours<br>• If participant is admitted to hospital, report using the routine follow-up CRF's and submit as per

	routine schedule.<br>• For other unrelated SAEs, do NOT report as part of this trial, report as per local reporting procedures.
10.5.1 Reporting of pregnancy	Additional guidance on reporting and following up pregnancies added.

18.4 Version 5.0 (21/JAN/2015)

Section	Amendments
Contact details section	Update of contact members and their details
Throughout protocol	Change in name of the Medicines for Children Research Network (MCRN) to Medicines for Children Clinical Trials Unit (MC-CTU)
4.2	Change to text to specify that consent for follow-up study should be taken on or near the 12 month follow-up visit.
5.2	Removal of the following form the eligibility criteria: Patient aged 8 years and over are able to comply with the treatment regimen and study visits .
5.3.3	Addition of the following text: If the participant explicitly states that they wish to withdraw from the trial completely and that they do not want their data to be included in the final analyses, please contact the SCIPI trial coordinator to request a Participant Data Withdrawal CRF
7.1	Addition of the following text based on new SmPC information: the safety and efficacy of insulin glargine (Lantus) have been established in adolescents and children aged 2 years and older. Insulin glargine has not been studied in children below the age of 2 years;
7.3 Formulation, Packaging, Labelling, Storage & Stability	Addition of information regarding Detemir (Levemir)
7.4 Preparation, Dosage and Administration of Study Treatment/s	Addition of text permitting use of the insulin Detemir if the MC-CTU have been notified.
7.4 Preparation, Dosage & Administration of Study Treatment/s	Addition of Omnipod as a pump that can be supplied in line with normal clinical practice
8.2.1 HbA1c	Process for collecting and processing of Hba1c blood

	samples made clearer
8.4 Other Assessments	Baseline questionnaire information present on a separate line from the 3 & 9 month information
8.6 Trial Closure	Change to definition of end of trial
10.4 Relationship to Trial Treatment	Addition of text to clarify that AE's and SAE's in related to trial treatment and disease under study should be reported
10.6 Reporting Procedures	Change to text clarifying that AE's and SAE's in related to trial treatment and disease under study should be reported
10.7 Follow-Up After Adverse Events	Change to text clarifying that AE's and SAE's in related to trial treatment and disease under study should be reported
10.9 Responsibilities - Investigators	Addition of text clarify procedures for handling and reporting serious and non-serious AI's
11.1 Ethical Considerations	Update to glargine (Lantus) information derived from the updated SmPC (2012)

18.5 Version 6.0 (16/FEB/2016)

**Section**	**Amendments**
Contact details section	Update of contact members and their details

18.6 Version 7.0 (01/AUG/2016)

**Section**	**Amendments**
Signature page	Addition of Trial Statistician's signature
1. Protocol Summary	Change to the Secondary Objective. HbA1c reduced from 7.5% to 6.5% and HbA1c also provided in mmol.
2.1 Introduction	Updated the HbA1c recommendations in line with the recent NICE guidance.
2.3 Objectives	This section has been amended to reflect the secondary objectives not the endpoints. The endpoints are listed in section 4.2.
4.2 Full trial	Change to secondary endpoint - Percentage of participants in each group with HbA1c reduced from <7.5% to <6.5%. Added partial remission and height as endpoints
9.3 Outcome measures	Added outcomes may be presented as mml or

	equivalent %
19. References	Addition of NICE 2015 Diabetes (type 1 and type 2) in children and young people: Type I Diabetes: Diagnosis and management

19 REFERENCES

(1) National Diabetes Audit: Key Findings about the quality of care for people with diabetes living in England incorporating registrations from Wales. 2006/2007.

(2) Alemzadeh R, Ellis JN, Holzum MK, Parton EA, Wyatt DT. Beneficial effects of continuous subcutaneous insulin infusion and flexible multiple daily insulin regimen using insulin glargine in type 1 diabetes. Pediatrics 2004;114(1):e91-95.

(3) Gale EAM. The rise of childhood type 1 diabetes in the 20th century. Diabetes 2002;51(12):3353-3361.

(4) Karvonen M. Incidence and trends of childhood Type 1 diabetes worldwide 1990-1999. Diabetic Med. 2006;23(8):857-866.

(5) Gardner SG, Bingley PJ, Sawtell PA, Weeks S, Gale EAM. Rising incidence of insulin dependent diabetes in children aged under 5 years in the Oxford region: Time trend analysis. Br.Med.J. 1997;315(7110):713-717.

(6) Shamoon H, Duffy H, Fleischer N, Engel S, Saenger P, Strelzyn M, et al. The effect of intensive treatment of diabetes on the development and progression of long-term complications in insulin-dependent diabetes mellitus. N.Engl.J.Med. 1993;329(14):977-986.

(7) National Institute for Clinical Excellence. Type I Diabetes: Diagnosis and management in children and young people. 2004.

(8) National Institute for Clinical Excellence. Continuous subcutaneous insulin infusion for the treatment of diabetes mellitus. 2008.

(9) National Institute for Clinical Excellence. Diabetes (type 1 and type 2) in children and young people: diagnosis and management. 2015.

(10) Juuliiusson PB, Graue M, Wentzel-Larsen T, Søvik O. The impact of continuous subcutaneous insulin infusion on health-related quality of life in children and adolescents with type 1 diabetes. Acta Paediatrica, International Journal of Paediatrics 2006;95(11):1481-1487.

(11) McMahon SK, Airey FL, Marangou DA, McElwee KJ, Carne CL, Clarey AJ, et al. Insulin pump therapy in children and adolescents: Improvements in key parameters of diabetes management including quality of life. Diabetic Med. 2005;22(1):92-96.

(12) Nimri R, Weintrob N, Benzaquen H, Ofan R, Fayman G, Phillip M. Insulin pump therapy in youth with type 1 diabetes: A retrospective paired study. Pediatrics 2006;117(6):2126-2131.

(13) Sulli N, Shashaj B. Long-term benefits of continuous subcutaneous insulin infusion in children with Type 1 diabetes: A 4-year follow-up. Diabetic Med. 2006;23(8):900-906.

(14) Sulli N, Shashaj B. Continuous subcutaneous insulin infusion in children and adolescents with diabetes mellitus: Decreased HbA1c with low risk of hypoglycemia. Journal of Pediatric Endocrinology and Metabolism 2003;16(3):393-399.

(15) Weintrob N, Schechter A, Benzaquen H, Shalitin S, Lilos P, Galatzer A, et al. Glycemic patterns detected by continuous subcutaneous glucose sensing in children and adolescents with type 1 diabetes mellitus treated by multiple daily injections vs continuous subcutaneous insulin infusion. Archives of Pediatrics and Adolescent Medicine 2004;158(7):677-684.

(16) Raile K, Noelle V, Landgraf R, Schwarz H. Weight in adolescents with type 1 diabetes mellitus during continuous subcutaneous insulin infusion (CSII) therapy. Journal of pediatric endocrinology & metabolism 2002;15(5):607-612.

(17) Hathout EH, McClintock T, Sharkey J, Kytsenko D, Hartwick N, Hadley-Scofield M, et al. Glycemic, auxologic, and seasonal aspects of continuous subcutaneous insulin infusion therapy in children and young adults with type 1 diabetes. Diabetes Technology and Therapeutics 2003;5(2):175-181.

(18) Alemzadeh R, Palma-Sisto P, Parton EA, Holzum MK. Continuous subcutaneous insulin infusion and multiple dose of insulin regimen display similar patterns of blood glucose excursions in pediatric type 1 diabetes. Diabetes Technology and Therapeutics 2005;7(4):587-596.

(19) Doyle EA, Weinzimer SA, Steffen AT, Ahern JAH, Vincent M, Tamborlane WV. A randomized, prospective trial comparing the efficacy of continuous subcutaneous insulin infusion with multiple daily injections using insulin glargine. Diabetes Care 2004;27(7):1554-1558.

(20) Valenzuela JM, Patino AM, McCullough J, Ring C, Sanchez J, Eidson M, et al. Insulin pump therapy and health-related quality of life in children and adolescents with type 1 diabetes. J.Pediatr.Psychol. 2006;31(6):650-660.

(21) Pańkowska E, Błazik M, Dziechciarz P, Szypowska A, Szajewska H. Continuous subcutaneous insulin infusion vs. multiple daily injections in children with type 1 diabetes: A systematic review and meta-analysis of randomized control trials. Pediatric Diabetes 2009;10(1):52-58.

(22) Weintrob N, Benzaquen H, Galatzer A, Shalitin S, Lazar L, Fayman G, et al. Comparison of continuous subcutaneous insulin infusion and multiple daily injection regimens in children with type 1 diabetes: A randomized open crossover trial. Pediatrics 2003;112(3 I):559-564.

(23) Nuboer R, Borsboom GJJM, Zoethout JA, Koot HM, Bruining J. Effects of insulin pump vs. injection treatment on quality of life and impact of disease in children with type 1 diabetes mellitus in a randomized, prospective comparison. Pediatric Diabetes 2008;9(4 PART 1):291-296.

(24) Rewers A, Peter Chase H, Mackenzie T, Walravens P, Roback M, Rewers M, et al. Predictors of acute complications in children with type 1 diabetes. J.Am.Med.Assoc. 2002;287(19):2511-2518.

(25) Greene S. on behalf of the Scottish Study Group for the Care of the Young Diabetic. Factors influencing glycaemic control in young people with Type 1 diabetes in Scotland: a population based study (Diabaud2). Diabetes Care 2001;24:239-244.

(26) McKinney PA, Feltbower RG, Stephenson CR, Reynolds C. Children and young people with diabetes in Yorkshire: A population-based clinical audit of patient data 2005/2006. Diabetic Med. 2008;25(11):1276-1282.

(27) Definition W. diagnosis of diabetes mellitus and intermediate hyperglycemia: report of a WHO/IDF consultation. Geneva: World Health Organization 2006.

(28) Colino E, Lopez-Capape M, Golmayo L, Alvarez MA, Alonso M, Barrio R. Therapy with insulin glargine (Lantus (R)) in toddlers, children and adolescents with type 1 diabetes. Diabetes Res.Clin.Pract. 2005;70(1):1-7.

(29) Swift PGF. ISPAD clinical practice consensus guidelines 2006-2007: Diabetes education. Pediatric Diabetes 2007;8(2):103-109.

(30) Walsh J, Roberts R. Pumping insulin: everything you need for success with an insulin Pump. : Torrey Pines Press; 2000.

(31) Cole TJ, Freeman JV, Preece MA. Body mass index reference curves for the UK, 1990. Arch.Dis.Child. 1995;73(1):25-29.

(32) Varni JW, Burwinkle TM, Jacobs JR, Gottschalk M, Kaufman F, Jones KL. The PedsQL™ in type 1 and type 2 diabetes: Reliability and validity of the Pediatric Quality of Life Inventory™ Generic Core Scales and Type 1 Diabetes Module. Diabetes Care 2003;26(3):631-637.

(33) De Wit M, Delemarre-van De Waal HA, Pouwer F, Gemke RJBJ, Snoek FJ. Monitoring health related quality of life in adolescents with diabetes: A review of measures. Arch.Dis.Child. 2007;92(5):434-439.

(34) Curtis L. Unit costs of health and social care 2007. 2007.

(35) Jones S, Rantell K, Stevens K, Colwell B, Ratcliffe JR, Holland P, et al. Outcome at 6 months after admission for pediatric intensive care: a report of a national study of pediatric intensive care units in the United kingdom. Pediatrics 2006;118(5):2101.

(36) National Institute of Health and Clinical Excellence.

(37) McCabe CJ, Stevens KJ, Brazier JE. Utility scores for the Health Utilities Index Mark 2: an empirical assessment of alternative mapping functions. Med.Care 2005;43(6):627.

(38) Glick H, Doshi JA, Sonnad SS, Polsky D. Economic evaluation in clinical trials. : Oxford University Press, USA; 2007.

(39) Manca A, Hawkins N, Sculpher MJ. Estimating mean QALYs in trial-based cost-effectiveness analysis: the importance of controlling for baseline utility. Health Econ. 2005;14(5):487-496.

(40) Palmer AJ, Roze S, Valentine WJ, Minshall ME, Foos V, Lurati FM, et al. The CORE Diabetes Model: projecting long-term clinical outcomes, costs and costeffectiveness of interventions in diabetes mellitus (types 1 and 2) to support clinical and reimbursement decision-making. Current Medical Research and Opinion® 2004;20(S1):5-26.
