# Supplementary material for: Continuous subcutaneous insulin infusion versus multiple daily injection regimens in children and young people at diagnosis of type 1 diabetes: pragmatic randomised controlled trial and economic evaluation
Source: BMJ. 2019 Apr 3;365:l1226. doi: 10.1136/bmj.l1226 (PMC6446076; doi:10.1136/bmj.l1226)
Supplement: Supplementary file 2 — Web appendix 2: Supplementary material [file blaj046029.ww2.pdf]

Table S1: Demographic characteristics of screened patients

		Consent obtained	Patient declined			
			MDI preference	CSII preference	Other reason	Total
**Age (years)**	N	293	259	36	100	395
	Mean	9.1	10	8.1	8.85	9.54
	SD	4.1	3.76	4.27	4.09	3.94
	Median	9.8	10.48	8.075	9.08	9.94
	Min	0.7	0.41	1.28	0.98	0.41
	Max	16	16	15.15	15.7	16
	Missing	1	0	0	0	0
**Age N(%)**	N	293	259	36	100	395
	Birth to 6 months	0 (0%)	1 (0.4%)	0 (0%)	0 (0%)	1 (0.3%)
	7 months to 4 years	65 (22.2%)	32 (12.4%)	9 (25%)	23 (23%)	64 (16.2%)
	5 to 11 years	147 (50.2%)	135 (52.1%)	18 (50%)	52 (52%)	205 (51.9%)
	12 to 15 years	81 (27.6%)	90 (34.7%)	9 (25%)	25 (25%)	124 (31.4%)
	16+ years	0 (0%)	1 (0.4%)	0 (0%)	0 (0%)	1 (0.3%)
	Missing	1	0	0	0	0
**Gender N(%)**	N	293	259	36	100	395
	Female	140 (47.8%)	121 (46.7%)	17 (47.2%)	46 (46%)	184 (46.6%)
	Male	153 (52.2%)	138 (53.3%)	19 (52.8%)	54 (54%)	211 (53.4%)
	Missing	1	0	0	0	0

**Ethnicity N(%)**	N	292	259	36	100	395
	Asian or Asian British	6 (2.1%)	4 (1.5%)	1 (2.8%)	4 (4%)	9 (2.3%)
	Black or British Black	3 (1%)	2 (0.8%)	2 (5.6%)	1 (1%)	5 (1.3%)
	British White	242 (82.9%)	228 (88%)	25 (69.4%)	81 (81%)	334 (84.6%)
	Chinese	0 (0%)	0 (0%)	0 (0%)	0 (0%)	0 (0%)
	Indian	4 (1.4%)	1 (0.4%)	0 (0%)	2 (2%)	3 (0.8%)
	Mixed	10 (3.4%)	6 (2.3%)	2 (5.6%)	1 (1%)	9 (2.3%)
	Not stated	3 (1%)	12 (4.6%)	1 (2.8%)	4 (4%)	17 (4.3%)
	Other	5 (1.7%)	1 (0.4%)	0 (0%)	1 (1%)	2 (0.5%)
	Other White	14 (4.8%)	3 (1.2%)	1 (2.8%)	5 (5%)	9 (2.3%)
	Pakistani	5 (1.7%)	2 (0.8%)	4 (11.1%)	1 (1%)	7 (1.8%)
	Missing	2	0	0	0	0
**Deprivation score* N(%)**	N	280	241	33	94	368
	Mean	23.26	24.01	28.51	21.65	23.81
	SD	18.53	18.19	16.57	16.17	17.6
	Median	17.045	17.96	27.65	17.06	18.225
	Min	1.62	1.18	3.9	2.95	1.18
	Max	77.23	74.35	63.86	71.91	74.35
	Missing	14	18	3	6	27

Table S2: Adherence to the study protocol

Protocol deviations	CSII (n = 144)	MDI (n = 149)	Overall (n = 293)
Deviations relating to inclusion and exclusion criteria	**0 (0%)**	**0 (0%)**	**0 (0%)**
Deviations relating to treatment and follow-up visits:			
**At least one major:**	57 (39.6%)	77 (51.7%)	134 (45.7%)
Start of study treatment from diagnosis being > 10 days (protocol v3)	1 (0.7%)	1 (0.7%)	2 (0.7%)
Start of study treatment from diagnosis being > 14 days (protocol v4)	1 (0.7%)	3 (2%)	4 (1.4%)
Scheduled 12 month follow-up visit falling outside the +/-15 day window	36 (25%)	49 (32.9%)	85 (29%)
Permanent change of insulin delivery	22 (15.3%)	31* (20.8%)	53* (18.1%)
Usage of non-protocol specified insulin**	10 (6.9%)	13 (8.7%)	23 (7.8%)
**At least one minor:**	**70 (48.6%)**	**87 (58.4%)**	**157 (53.6%)**
**At least three minor:**	**0 (0%)**	**0 (0%)**	**0 (0%)**
Scheduled 3 month follow-up visit falling outside the +/-15 day window	34 (23.6%)	44 (29.5%)	78 (26.6%)
Scheduled 6 month follow-up visit falling outside the +/-15 day window	43 (29.9%)	48 (32.2%)	91 (31.1%)
Scheduled 9 month follow-up visit falling outside the +/-15 day window	44 (30.6%)	39 (26.2%)	83 (28.3%)
**At least one major and/or at least three minor**:	**57 (39.6%)**	**77 (51.7%)**	**134 (45.7%)**

* One permanent switch of insulin delivery method was from MDI to 'Injections TDS regime'

** Does not include Levemir/Detemir.

NB Participants with at least one major protocol deviation or ≥3 minor deviations were excluded from the per protocol analysis.

Figure S1: Retention and adherence to the study protocol

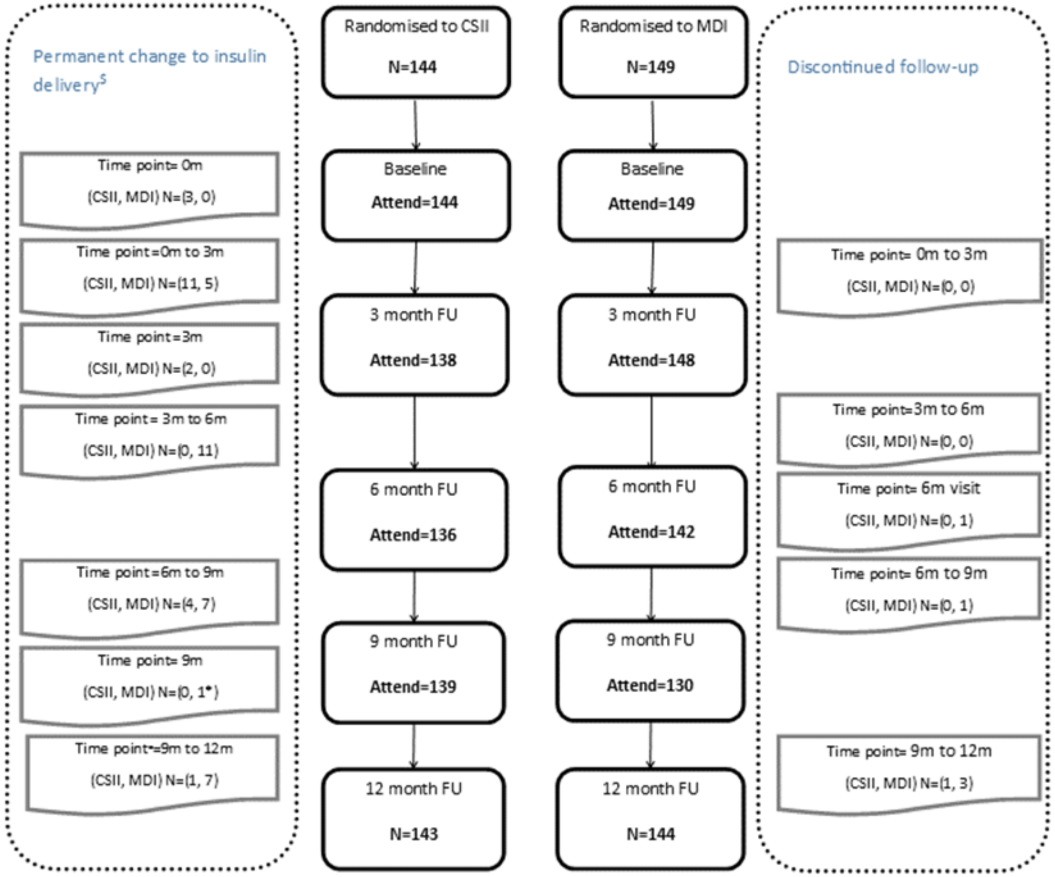

$ Permanent change of insulin delivery from randomised treatment but continuing follow-up.

* Patient changed from MDI to 'Injections TDS regime

Table S3: HbA1c measured at 12 months by age group: Intention to treat and per protocol analyses

Age-group	HbA1c (mmol/mol) at 12 months	CSII	MDI	Total	Adjusted[a] mean[d] CSII (95% CI)	Adjusted[a] mean[d] MDI (95% CI)	Adjusted[a] mean Difference (CSII-MDI) (95% CI)	P-value
**Primary analysis: Intention to treat analysis**								
**7mths - < 5yrs**	N (missing)[b]	33 (0)	31 (1)	64 (1)	60.9 (58.5,63.3)	58.5 (56.1,60.9)	2.4 (-0.4, 5.3)	0.09
	Mean (SD)	63.9 (12.1)	58.4 (9.9)	61.2 (11.3)				
**5yrs - <12yrs**	N(missing)[b]	70 (1)	72 (4)	142 (5)				
	Mean (SD)	58 (11.4)	59.3 (11.4)	58.7 (11.4)				
**12yrs - <16yrs**	N (missing)[b]	40 (0)	39 (2)	79 (2)				
	Mean (SD)	61.3 (13.3)	54.7 (14.7)	58.1 (14.3)				
**Overall**	N (missing)[b]	143 (1)	142 (7)	285 (8)				
	Mean (SD)	60.3 (12.3)	57.9 (12.2)	59.1 (12.3)				
**Per protocol analysis**								
**7mths - < 5yrs**	N (missing)[c]	23 (10)	11 (21)	34 (31)	60.2 (56.4,63.9)	59.3 (55.3,63.3)	0.9 (-3.2, 5)	0.67
	Mean (SD)	62.6 (13.1)	56.2 (11)	60.5 (12.6)				
**5yrs - <12yrs**	N (missing)[c]	41(30)	32 (44)	73 (74)				
	Mean (SD)	57.9 (11.8)	59.7 (10.8)	58.7 (11.3)				
**12yrs - <16yrs**	N (missing)[c]	23 (17)	23 (18)	46 (35)				
	Mean (SD)	57.6 (14.2)	57.8 (16.8)	57.7 (15.4)				
**Overall**	N (missing)[b]	87 (57)	66 (83)	153 (140)				
	Mean (SD)	59 (12.8)	58.4 (13.1)	58.8 (12.9)				

a=Adjusted for randomisation strata (age category – fixed effects; centre – random effects); b=missing values in ITT due to 5 withdrawals prior to 12 months and 1 missing value; c=missing values in per protocol analysis due to 6 withdrawals prior to 12 months, 2 missing value and 132 protocol deviations; d=Not pre-specified in the Statistical Analysis Plan but has been added to aid interpretation

Table S4: Post-hoc sensitivity analyses of primary outcome

	Adjusted* Mean CSII (95% CI)	Adjusted* Mean MDI (95% CI)	Adjusted* Mean Difference between treatment groups (CSII-MDI) across all age-groups (95% CI)
**ITT i.e. central in preference to POC (if available)**	n=143	n=142	n=285
	60.9 (58.5,63.3)	58.5 (56.1,60.9)	2.4 (-0.4, 5.3)
**Sensitivity analyses**			
**POC in preference to central (if available)**	n=143	n=142	n=285
	61.1 (58.3,63.8)	59 (56.3,61.8)	2 (-0.7, 4.8)
**Central only**	n=114	n=118	n=232
	60.7 (58.3,63.1)	57.9 (55.5,60.2)	2.9 (-0.3, 6)
**POC only**	n=141	n=137	n=278
	61.1 (58.3,63.8)	59 (56.3,61.8)	2 (-0.7, 4.8)

* Adjusted for randomisation strata (age category – fixed effects; centre – random effects).

Table S5: Post-hoc analysis of primary outcome adjusting for baseline HbA1c

	Effect estimate	Standard error	95% CI	p-value	Adjusted* mean CSII (95% CI)	Adjusted* mean MDI (95% CI)	Adjusted* mean Difference (CSII-MDI) (95% CI)	P-value
**Intercept**	53.84	3.38	(46.60, 61.09)	<0.001	61.29 (58.77, 63.81)	58.36 (55.76, 60.96)	2.93 (-0.02, 5.87)	0.0517
**HbA1c at baseline**	0.07	0.03	(0.01, 0.13)	0.0264				
**Treatment**								
MDI	Reference							
CSII	2.93	1.50	(-0.02, 5.87)	0.0517				
**Age strata**								
7mths - < 5yrs	Reference							
5yrs - <12yrs	-4.09	2.02	(-8.08, -0.11)	0.0440				
12yrs - <16yrs	-4.29	2.28	(-8.79, 0.20)	0.0612				

* Adjusted for randomisation strata (age category – fixed effects; centre – random effects) and baseline HbA1c.

Figure S2: Post-hoc HbA1c mean profile plots by age strata A: Participants age < 5 years; B: Participants age 5-11 years and C: Participants aged > 12 years

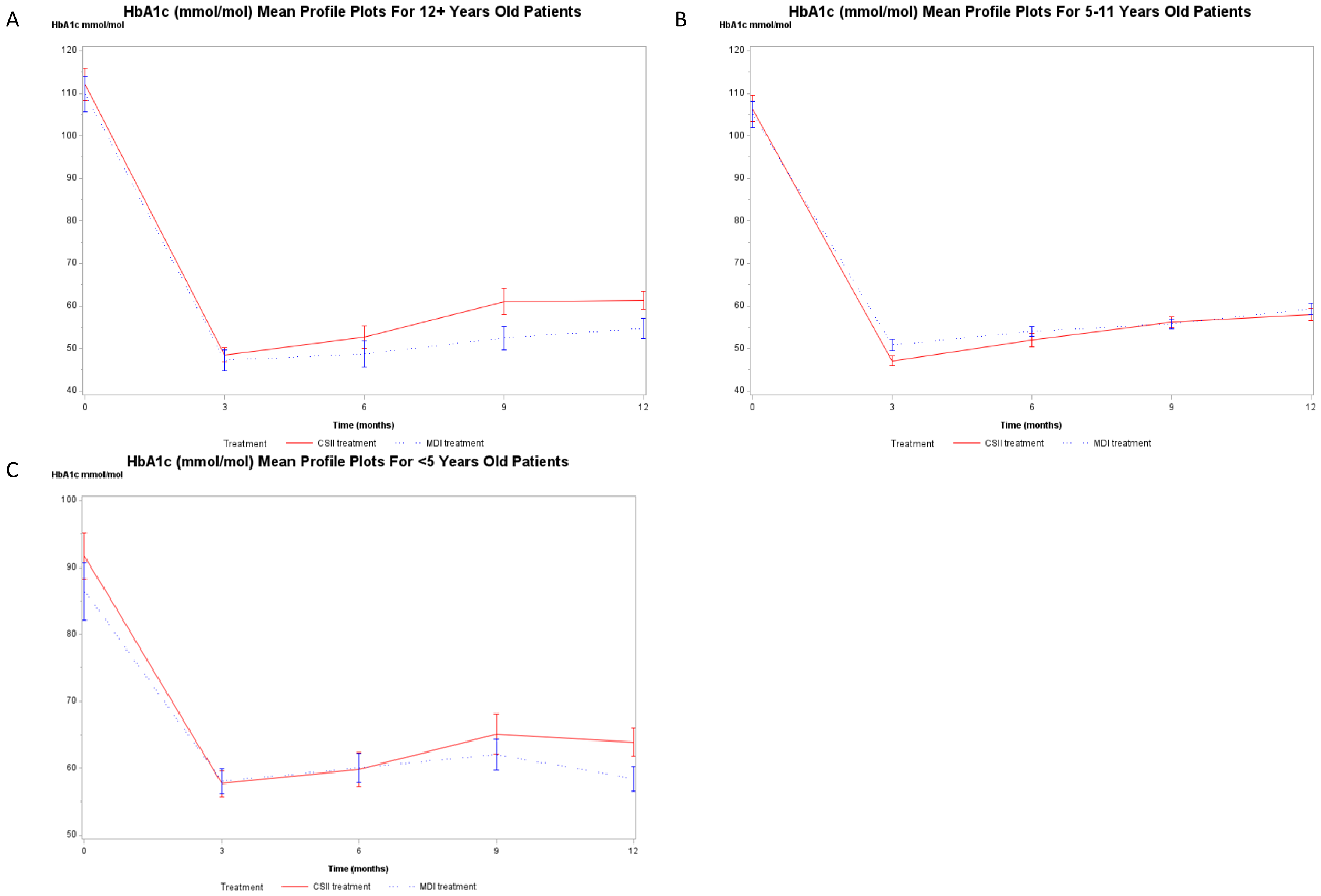

Figure S3: Post-hoc Forest plot to consider treatment effect learning curve based on how long each site had been open to recruitment

	CSII			MDI				Mean Difference
Study or Subgroup	Mean	SD	Total	Mean	SD	Total	Weight	IV, Fixed, 95% CI
**1.2.1 Site**								
01: 0-0.5 years	59.83125	11.24944264	32	58.17142857	12.02078033	35	12.3%	1.66 [-3.91, 7.23]
02: 0.5-1 year	61	9.6695398	25	57.23809524	11.28230811	21	10.2%	3.76 [-2.37, 9.90]
03: 1-1.5 years	59.80686724	14.08933527	29	64.39130435	13.56043419	23	6.7%	-4.58 [-12.13, 2.97]
04: 1.5-2 years	61.09090909	12.89115271	22	52.82142857	8.80678851	28	9.7%	8.27 [1.97, 14.57]
05: 2-2.5 years	56.38461538	11.14703594	13	55	11.54700538	16	5.6%	1.38 [-6.91, 9.67]
06: 2.5-3 years	61.625	16.69281282	16	60.61538462	14.76221789	13	2.9%	1.01 [-10.45, 12.47]
07: 3-3.5 years	64	4.24264069	2	56.5	6.8556546	4	4.8%	7.50 [-1.43, 16.43]
08: 3.5-4 years	63.75	6.39661369	4	61.5	26.1629509	2	0.3%	2.25 [-34.55, 39.05]
**Subtotal (95% CI)**			**143**			**142**	**52.5%**	**2.96 [0.26, 5.66]**
Heterogeneity: Chi² = 8.09, df = 7 (P = 0.33); I² = 13%								
Test for overall effect: Z = 2.15 (P = 0.03)								
**1.2.2 Overall**								
Overall	60.27971434	12.2673033	143	57.85211268	12.20565337	142	47.5%	2.43 [-0.41, 5.27]
**Subtotal (95% CI)**			**143**			**142**	**47.5%**	**2.43 [-0.41, 5.27]**
Heterogeneity: Not applicable								
Test for overall effect: Z = 1.67 (P = 0.09)								
**Total (95% CI)**			**286**			**284**	**100.0%**	**2.71 [0.75, 4.66]**

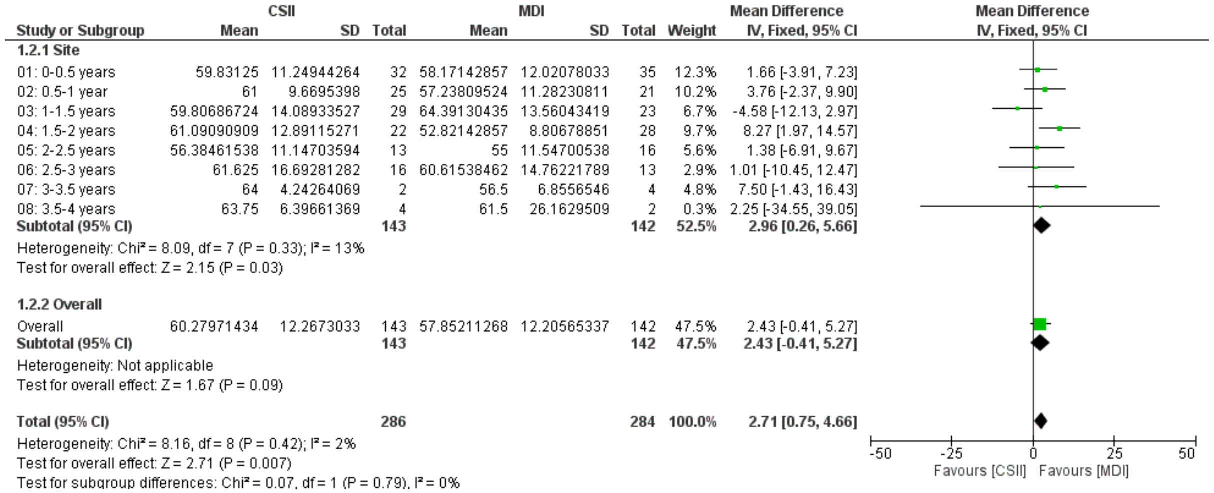

Heterogeneity: Chi² = 8.16, df = 8 (P = 0.42); I² = 2%
Test for overall effect: Z = 2.71 (P = 0.007)
Test for subgroup differences: Chi² = 0.07, df = 1 (P = 0.79), I² = 0%

Figure S4: Primary outcome Forest plots split by subgroup

Primary outcome Forest plot split by age strata (post-hoc analysis):

Study or Subgroup	CSII Mean	CSII SD	CSII Total	MDI Mean	MDI SD	MDI Total	Weight	Mean Difference IV, Fixed, 95% CI
**1.5.1 Site**								
01: 7mths to less than 5yrs	63.8787621	12.0828207	33	58.4193548	9.9457669	31	13.4%	5.46 [0.05, 10.87]
02: 5yrs to less than 12yrs	57.9857143	11.3972955	70	59.3055556	11.4493851	72	27.8%	-1.32 [-5.08, 2.44]
03: 12yrs to less than 16yrs	61.325	13.2962063	40	54.7179487	14.6771371	39	10.3%	6.61 [0.43, 12.79]
**Subtotal (95% CI)**			**143**			**142**	**51.4%**	**2.03 [-0.73, 4.79]**
Heterogeneity: Chi² = 6.70, df = 2 (P = 0.04); I² = 70%								
Test for overall effect: Z = 1.44 (P = 0.15)								
**1.5.2 Overall**								
Overall	60.27971434	12.2673033	143	57.85211268	12.20565337	142	48.6%	2.43 [-0.41, 5.27]
**Subtotal (95% CI)**			**143**			**142**	**48.6%**	**2.43 [-0.41, 5.27]**
Heterogeneity: Not applicable								
Test for overall effect: Z = 1.67 (P = 0.09)								
**Total (95% CI)**			**286**			**284**	**100.0%**	**2.22 [0.24, 4.20]**
Heterogeneity: Chi² = 6.74, df = 3 (P = 0.08); I² = 56%								
Test for overall effect: Z = 2.20 (P = 0.03)								
Test for subgroup differences: Chi² = 0.04, df = 1 (P = 0.84), I² = 0%								

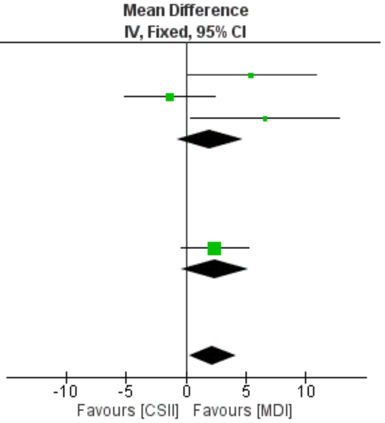

Primary outcome Forest plot split by sites:

Study or Subgroup	CSII Mean	CSII SD	CSII Total	MDI Mean	MDI SD	MDI Total	Weight	Mean Difference IV, Fixed, 95% CI
**1.4.1 Site**								
01	60.7555	11.9268604	4	49.25	8.0156098	4	1.9%	11.51 [-2.58, 25.59]
02	55.25	10.9642275	8	59.6666667	11.6404467	9	3.3%	-4.42 [-15.17, 6.33]
03	0	0	0	57	4.2426407	2		Not estimable
04	64.2142857	15.5324899	14	61.1333333	13.0376743	15	3.5%	3.08 [-7.39, 13.56]
05	61.5	13.541183	12	59.5555556	11.7910041	9	3.2%	1.94 [-8.92, 12.81]
06	56.6666667	8.3864971	3	48.3333333	4.7258156	3	3.2%	8.33 [-2.56, 19.23]
07	0	0	0	67.5	7.7781746	2		Not estimable
08	57.7741935	11.1376529	31	55.8666667	13.6879845	30	9.7%	1.91 [-4.37, 8.18]
09	58.6923077	11.3517196	26	55.1666667	12.7370281	24	8.5%	3.53 [-3.18, 10.23]
10	64	13.2916014	4	52.6666667	10.7857931	3	1.2%	11.33 [-6.52, 29.18]
11	61.2	4.5077711	3	69.25	10.8435849	4	2.8%	-8.05 [-19.84, 3.74]
12	61.07983	12.6936387	5	61.6666667	11.2368442	6	1.9%	-0.59 [-14.89, 13.72]
13	59.3333333	9.8361578	9	61.5555556	9.0015431	9	5.0%	-2.22 [-10.93, 6.49]
14	62.3333333	15.8336945	18	54.7647059	10.9231944	17	4.8%	7.57 [-1.40, 16.54]
15	68.3333333	8.2623645	6	68.8	9.2032603	5	3.5%	-0.47 [-10.90, 9.96]
**Subtotal (95% CI)**			**143**			**142**	**52.6%**	**2.16 [-0.54, 4.86]**
Heterogeneity: Chi² = 11.21, df = 12 (P = 0.51); I² = 0%								
Test for overall effect: Z = 1.57 (P = 0.12)								
**1.4.2 Overall**								
Overall	60.27971434	12.2673033	143	57.85211268	12.20565337	142	47.4%	2.43 [-0.41, 5.27]
**Subtotal (95% CI)**			**143**			**142**	**47.4%**	**2.43 [-0.41, 5.27]**
Heterogeneity: Not applicable								
Test for overall effect: Z = 1.67 (P = 0.09)								
**Total (95% CI)**			**286**			**284**	**100.0%**	**2.29 [0.33, 4.24]**
Heterogeneity: Chi² = 11.23, df = 13 (P = 0.59); I² = 0%								
Test for overall effect: Z = 2.29 (P = 0.02)								
Test for subgroup differences: Chi² = 0.02, df = 1 (P = 0.89), I² = 0%								

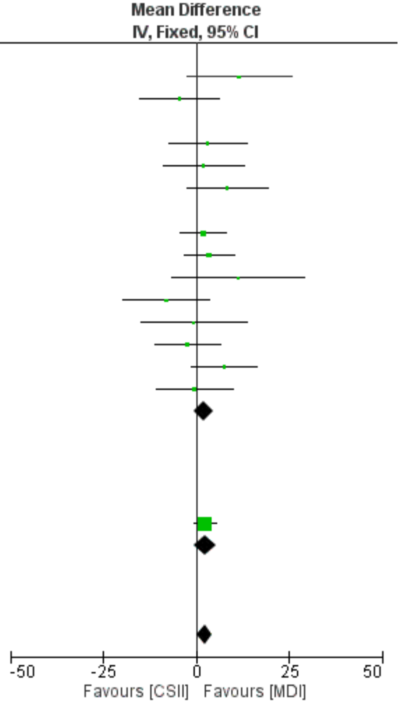

Figure S5: Post-hoc mean profile plot of average monthly basal/bolus ratio across the 12 months for CSII patients

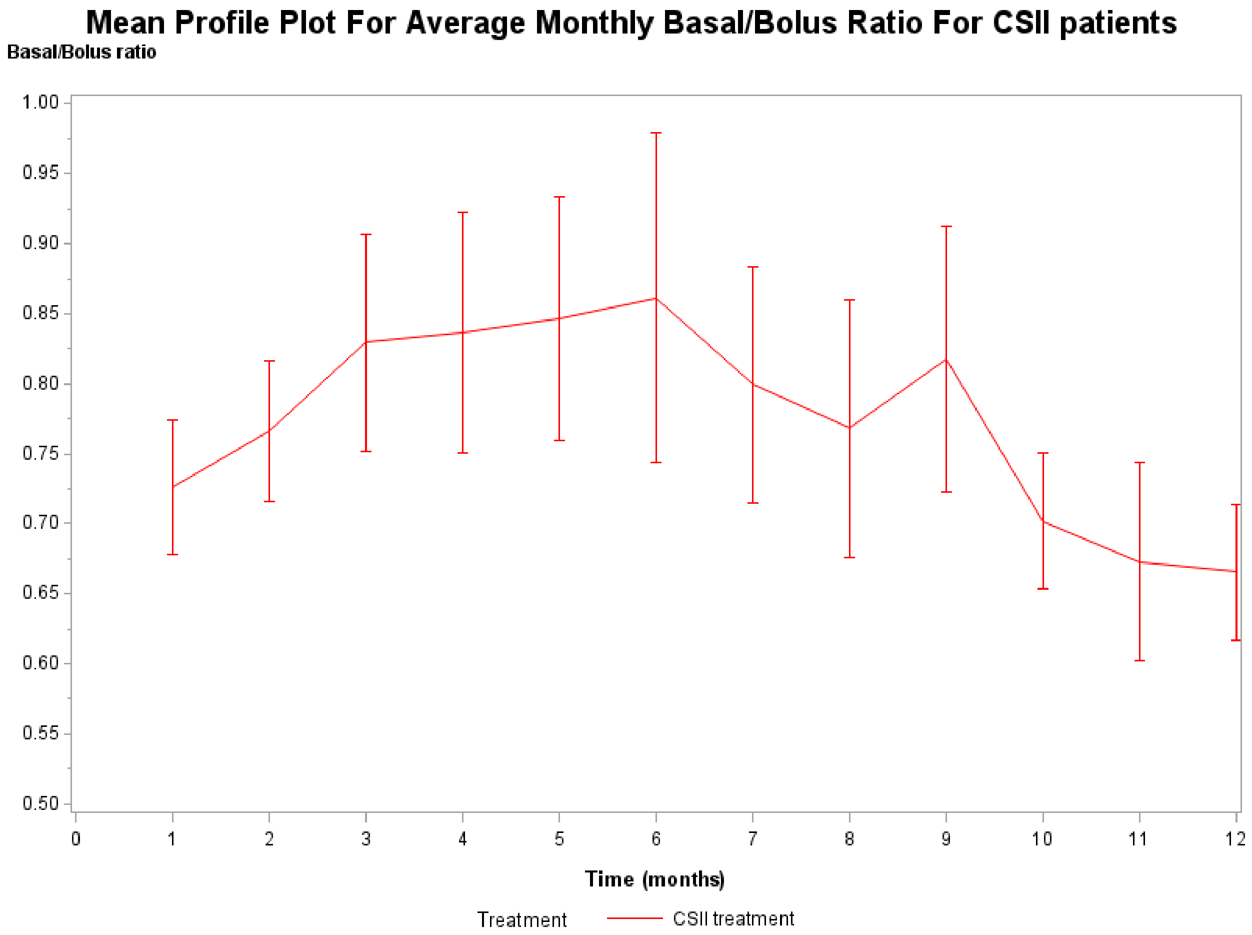

Table S6: Post-hoc summary statistics to show distribution of average monthly basal/bolus ratio across the 12 months for CSII patients

	Month											
	**1**	**2**	**3**	**4**	**5**	**6**	**7**	**8**	**9**	**10**	**11**	**12**
**N**	51	72	80	71	66	67	63	63	74	64	63	69
**Mean±SD**	0.73±0.34	0.77±0.43	0.83±0.7	0.84±0.73	0.85±0.71	0.86±0.97	0.8±0.67	0.77±0.73	0.82±0.82	0.7±0.39	0.67±0.56	0.67±0.41
**Median (Q1-Q3)**	0.65 (0.51-0.92)	0.67 (0.49-1.01)	0.64 (0.44-0.96)	0.62 (0.46-0.99)	0.66 (0.44-0.9)	0.61 (0.41-0.87)	0.67 (0.46-0.94)	0.53 (0.43-0.91)	0.6 (0.46-0.84)	0.61 (0.47-0.87)	0.54 (0.42-0.76)	0.6 (0.43-0.77)
**Min-Max**	0.2-1.59	0.14-2.13	0-4.78	0.11-5.34	0.14-4.07	0.14-6.27	0.08-4.39	0.08-4.36	0.08-5.83	0.23-2.12	0-3.76	0.15-2.45

Table S7: Additional Analyses – Primary efficacy assessment – HbA1c measured 12 months after randomisation adjusted for deprivation score

Deprivation score (continuous) as a covariate								
	Effect estimate	Standard error	95% CI	p-value	Adjusted[a] mean CSII (95% CI)	Adjusted[a] mean MDI (95% CI)	Adjusted[a] mean Difference (CSII-MDI) (95% CI)	P-value
**Intercept**	59.26	2.05	(54.86, 63.65)	<.0001	60.57 (57.97, 63.17)	58.41 (55.83, 60.99)	2.16 (-0.72, 5.04)	0.1414
**Deprivation score**	0.03	0.04	(-0.05, 0.12)	0.4273				
**Treatment**								
MDI	Reference							
CSII	2.16	1.46	(-0.72, 5.04)	0.1414				
**Age strata**								
7mths - < 5yrs	Reference							
5yrs - <12yrs	-2.16	1.87	(-5.85, 1.53)	0.2495				
12yrs - <16yrs	-2.74	2.07	(-6.83, 1.34)	0.1868				
Deprivation score (quintile) as a covariate								

	Effect estimate	Standard error	95% CI	p-value	Adjusted[b] mean CSII (95% CI)	Adjusted[b] mean MDI (95% CI)	Adjusted[b] mean Difference (CSII-MDI) (95% CI)	P-value
**Intercept**	61.18	2.33	(56.19, 66.17)	<.0001	60.97 (58.32 63.63)	58.80 (56.16, 61.44)	2.17 (-0.70, 5.04)	0.1377
**Deprivation score**								
1 (<=8.49)	-1.86	2.18	(-6.15, 2.43)	0.3935				
2 (8.5 – 13.79)	-3.32	2.28	(-7.81, 1.16)	0.1454				
3 (13.8 – 21.35)	3.21	2.42	(-1.57, 7.98)	0.1870				
4 (21.36 – 34.17)	-0.10	2.42	(-4.86, 4.66)	0.9662				
5 (>=34.18)	Reference							
**Treatment**								
MDI	Reference							
CSII	2.17	1.46	(-0.70, 5.04)	0.1377				
**Age strata**								
7mths - < 5yrs	Reference							
5yrs - <12yrs	-2.70	1.88	(-6.40, 0.99)	0.1510				
12yrs - <16yrs	-3.18	2.07	(-7.26, 0.89)	0.1249				

a Adjusted for randomisation strata (age category – fixed effects; centre – random effects) and baseline deprivation score (continuous).
b Adjusted for randomisation strata (age category – fixed effects; centre – random effects) and baseline deprivation score (quintile).

The SCIPI Investigators

Birmingham Children’s Hospital: Professor Tim Barrett

Royal Blackburn Hospital: Dr Chris Gardener

University Hospital of Wales, Cardiff: Professor John Gregory

Doncaster Royal Infirmary: Dr Anuja Natarajan

Ipswich Hospital: Dr Jackie Buck

Alder Hey Children’s Hospital, Liverpool: Dr Atrayee Ghatak

Mid Staffordshire NHS Foundation Trust: Dr Olumuyiwa Oso

Great North Children’s Hospital, Newcastle: Dr Tim Cheetham

Norfolk and Norwich University NHS Foundation Trust: Dr Nandu Thalange

Nottingham Children’s Hospital: Dr Tabitha Randell

Oxford Children’s Hospital: Dr Julie Edge

Royal Preston Hospital: Dr Omolola Ayoola,

Sheffield Children’s Hospital: Dr Neil Wright

Southampton Children’s Hospital: Dr Nicola Trevelyan

East Surrey Hospital: Dr. Neemisha Jain
